# A Network and Pathway Analysis of Genes Associated With Atrial Fibrillation

**DOI:** 10.1155/2024/7054039

**Published:** 2024-10-05

**Authors:** Mengying Zeng, Xian Yang, Yunhao Chen, Jinqi Fan, Li Cao, Menghao Wang, Peilin Xiao, Zhiyu Ling, Yuehui Yin, Yunlin Chen

**Affiliations:** ^1^Department of Cardiology, The Second Affiliated Hospital of Chongqing Medical University, Chongqing, China; ^2^Chongqing Key Laboratory of Cardiac Electrophysiology, Chongqing, China; ^3^Cardiac Arrhythmia Intervention Center of Chongqing Medical Quality Control Center, Chongqing, China; ^4^Chongqing Atrial Fibrillation Center Alliance, Chongqing, China; ^5^Chengdu Medical College, Chengdu, China; ^6^Department of Hepatobiliary Surgery, The Second Affiliated Hospital of Chongqing Medical University, Chongqing, China

**Keywords:** atrial fibrillation, genetic association study, microarray meta-analysis, network and pathway analysis

## Abstract

**Background:** Atrial fibrillation (AF) is affected by both environmental and genetic factors. Previous genetic association studies, especially genome-wide association studies, revealed a large group of AF-associated genes. However, little is known about the functions and interactions of these genes. Moreover, established genetic variants of AF contribute modestly to AF variance, implying that numerous additional AF-associated genetic variations need to be identified. Hence, a systematic network and pathway analysis is needed.

**Methods:** We retrieved all AF-associated genes from genetic association studies in various databases and performed integrative analyses including pathway enrichment analysis, pathway crosstalk analysis, network analysis, and microarray meta-analysis.

**Results:** We collected 254 AF-associated genes from genetic association studies in various databases. Pathway enrichment analysis revealed the top biological pathways that were enriched in the AF-associated genes related to cardiac electromechanical activity. Pathway crosstalk analysis showed that numerous neuro-endocrine-immune pathways connected AF with various diseases including cancers, inflammatory diseases, and cardiovascular diseases. Furthermore, an AF-specific subnetwork was constructed with the prize-collecting Steiner forest algorithm based on the AF-associated genes, and 24 novel genes that were potentially associated with AF were inferred by the subnetwork. In the microarray meta-analysis, six of the 24 novel genes (*APLP1*, *CREB1*, *CREBBP*, *PRMT1*, *IRAK1*, and *PLXND1*) were expressed differentially in patients with AF and sinus rhythm.

**Conclusions:** AF is not only an isolated disease with abnormal electrophysiological activity but might also share a common genetic basis and biological process with tumors and inflammatory diseases as well as cardiovascular diseases. Moreover, the six novel genes inferred from network analysis might help detect the missing AF risk loci.

## 1. Introduction

Atrial fibrillation (AF), the most common arrhythmia, is a major cause of stroke, heart failure, and cardiovascular death worldwide [1]. Although the mechanisms of AF are not fully understood, genetic factors are clearly involved in the occurrence of AF [2].

Over the past few decades, genetic association studies have reported a large group of AF-associated genes. Classical Mendelian genetics and candidate gene approaches have identified some mutations in ion channel genes including *KCNQ1*, *SCN5A*, and *SCN2B* [3–5]. Genome-wide association studies (GWASs) have also identified independent susceptibility signals for AF at various genomic regions. These include *PITX2*, *KCNN3*, *CAV1*, and *HCN4*, among others [6, 7]. Traditional experiments have already succeeded in elucidating the roles of several AF-associated genes, such as *PITX2*, in the occurrence of AF [8]. Mutations in transcription factors, such as PITX2, may result in modifications to the expression of target genes implicated in ion channel regulation, calcium handling, or myocardial fibrosis. These changes could potentially trigger AF or establish a substrate supporting its perpetuation [9]. Nevertheless, summarizing the underlying biological information of AF from the extensive genotype data is still a great challenge. In addition, common genetic variants can explain 20.4% of AF heritability, but only 5% of AF heritability can be attributed to the currently known risk loci [10]. This finding implies that efforts should be made in the future to identify the missing risk loci and to understand the causal genetic basis of AF by the established genetic variants. Hence, a comprehensive analysis of AF-associated genes within a pathway and/or network framework is urgently needed.

Pathway and network-assisted analyses have been applied to numerous complex diseases, such as nervous system disease, coronary artery disease, and infectious disease; however, their application in AF has been limited [11–13]. This systems biology approach is based on the concept that instead of working in isolation, disease genes interact through complex molecular networks and cellular pathways to control disease susceptibility and progression [14]. By taking into account prior biological knowledge about genes and pathways, we may obtain important and unique insights into the pathogenic mechanisms underlying disease-associated genes. Moreover, the disease-specific subnetwork can be constructed with the prize-collecting Steiner forest (PCSF) algorithm [15]. The subnetwork includes as many known disease genes as possible with the most reliable associated interactions and, at the same time, may include additional genes. These additional genes that are detected based on the principles of “guilt by association” in the biological process have strong associations with the known disease genes in the disease-specific subnetwork. The additional genes may help identify the missing risk loci.

In this study, a comprehensive compilation of genes associated with AF was performed. Our methodology adhered to strategies employed in previous studies, which included searches in reputable phenotype–genotype databases and manual retrieval of relevant literature from PubMed [13, 16]. Each publication underwent a systematic review to ensure a thorough collection of genes associated with AF. Next, by using pathway analyses, we sought to elucidate the biological functions of these genes and the interactions between them and further shed light on the disorders and pathways associated with AF. The AF-specific molecular network was constructed in the background of the human interactome with the known AF-associated genes. Furthermore, additional genes that were potentially associated with AF were also inferred by the subnetwork. The stronger the association between the gene and AF was, the more likely the perturbation of its expression level in patients with AF would be [11]. Hence, we conducted a microarray meta-analysis to evaluate these additional genes at the transcriptome level and thus identify the potential AF-associated genes deserving further genetic association studies. Ultimately, our work provides a practical framework for understanding the molecular mechanisms of AF at the functional level and explores the future research direction.

## 2. Materials and Methods

### 2.1. Identification of the AF-Related Gene

A literature search in PubMed (October 28, 2017) of genes related to AF was performed by two independent investigators (Yunlin Chen and Mengying Zeng). The following search terms were used to identify the genetic association studies related to AF as reported [12, 16, 17]: AF, gene, genetic variants, polymorphism, and genotype, as well as alleles. By careful review of the abstracts, we only selected studies that reported one or more genes having significant relationships with AF. The full text of each study was further evaluated, and the significant genes were included for subsequent analyses. To minimize the number of false-positive findings, studies reporting negative or insignificant associations between the genes and AF were excluded, acknowledging that some genes might still be related to AF. Two other public data sources were also employed to collect and curate robust data on AF-associated genes: (1) the Human Genome Epidemiology (HuGE) Navigator, a database of gene-phenotype associations extracted from published studies by automated text-mining [18], and (2) the Phenotype–Genotype Integrator (PheGenI), which provides the NHGRI GWAS catalog data [19]. Considering that insignificant genes were collected in the HuGE Navigator, the search results of this database were further reviewed to exclude the insignificant genes. The GWAS data in PheGenI with *p* values < 10^−5^ were retrieved [11, 12, 20]. After removing repeated genes, we compiled a list of AF candidate genes (AFgenes) for subsequent analyses.

### 2.2. Pathway Enrichment Analysis

The biochemical pathways enriched among the AFgenes were analyzed by ClueGO (a Cytoscape plug-in) to uncover the underlying biological meanings [21]. Specifically, AFgenes were compared with the genes included in each canonical pathway deposited in the Kyoto Encyclopedia of Genes and Genomes (KEGG; http://www.genome.jp/kegg) and Reactome (http://www.reactome.org) pathway databases. All of the pathways with more than three genes overlapping the AFgenes were extracted, each of which was assigned a *p* value to represent the overlap significance between the pathway and the AFgenes via the hypergeometric test. Each *p* value was corrected with the Benjamini–Hochberg method, and only the pathways with adjusted *p* values<0.05 were considered to be significantly enriched pathways.

### 2.3. Pathway Crosstalk Analysis

The relationship between pathways is based on their shared genes in a similar way [22]. To reveal the interactions between the significantly enriched pathways, we performed the pathway crosstalk analysis by ClueGO [21]. Basically, a gene-pathway matrix was constructed with the significant pathways and the associated genes; a pathway–pathway similarity matrix was calculated using chance-corrected kappa statistics based on the first matrix; the cutoff level of the kappa score was set at 0.4 to restrict the network connectivity; the pathway–pathway network was constructed based on the second matrix and the kappa score threshold; and the network was finally laid out with the organic layout algorithm.

### 2.4. Construction of the Human Interactome

With the goal of unraveling the interactions among the AFgenes, a relatively comprehensive and reliable human protein interaction network (human interactome) was constructed. The human interactome data were downloaded from the interaction reference index (iRefIndex), which is a consolidated protein interaction database. This database includes data available in nine interaction databases, namely, BIND, BioGRID, DIP, HPRD, IntAct, MINT, MPact, MPPI, and OPHI [23]. Additionally, the interactions in the interactome were scored based on their experimental data using the MIScore function [24].

### 2.5. Construction of AF-Specific Network by the PCSF Algorithm

Integrating the human interactome and disease-associated genes generates a meaningful biological context in terms of functional associations for these genes, which provides important insight into the molecular basis of the disease [25]. Based on the principles of “guilt by association” and “parsimony” in biological processes, the disease-specific subnetwork should include all of the most confident paths between all or most genes of interest, which can be expressed as a PCSF problem [26]. Over recent decades, several researchers have employed different approaches to solve this problem and have successfully identified robust and reliable disease-specific subnetworks for different diseases [27]. In this study, we applied the Forest module of the Omics Integrator Software, a well-received Python package, to tackle this problem [15]. Different parameters greatly affect the final subnetwork, and therefore, we first ran the Forest module over a range of parameters (w: 1, 5, 10; b: 1, 5, 10; D: 5) to find the best parameter set for our case. Parameter w influences the number of distinct trees identified. Parameter b governs the trade-off between the enhancement of terminal nodes and the incorporation of less reliable edges. Parameter D regulates the maximum path length from the root to the leaf nodes of the tree. In accordance with the software's guidelines, Parameters w and b are recommended to be set within the range of 1–10. Regarding Parameter D, the value of 5 is advised.

A good parameter set can maximize the fraction of input prize nodes (the AFgenes) in the final subnetwork, minimize the distance between the average degree of prize nodes and hidden nodes (genes inferred by the PCSF algorithm that are highly related to the AFgenes but not among the AFgenes), and construct a larger network that is robust in the context of noise [28]. Initially, the software Cytoscape was utilized to calculate the average degree of prize and hidden nodes across different subnetworks extracted using the parameter sets above [29]. Subsequently, we assessed the robustness and specificity of these subnetworks extracted by the advised parameter sets. We added Gaussian noise to the edge weight and reran the Forest module 10 times; we randomly redistributed our prizes within the interactome and reran Forest 10 times. The “FractionOfOptimalForestsContaining” is an output attribute representing the fraction of optimal forests containing each node. This attribute indicates how frequently a node appears across multiple forest runs with noise or random inputs. A robust and specific network had most nodes with high “FractionOfOptimalForestsContaining” values in the noisyEdges run, with low “FractionOfOptimalForestsContaining” values in the randomTerminals run. The importance of hidden nodes can also be assessed by the “FractionOfOptimalForestsContaining” values. Finally, Cytoscape was employed to visualize the final optimal subnetwork.

### 2.6. Microarray Meta-Analysis for the Assessment of the Hidden Genes

If genes have a strong association with AF, the expression levels of these genes are more likely to be perturbed in patients with AF [11]. With the increasing number of publicly available microarray datasets, merging them with a powerful in silico tool can generate a robust and reliable list of differentially expressed genes (DEGs). This method avoids the artifacts of individual studies and can offer an opportunity for us to determine which novel genes inferred by the network analysis merit further genetic association studies in the future [11, 30].

The Gene Expression Omnibus (http://www.ncbi.nlm.nih.gov/geo/) was used to search publicly available gene expression datasets for AF studies with at least 10 samples. The preparation of gene expression data matrixes for the meta-analysis followed the end-to-end workflow provided by Bernd Klaus [31]. The quality of the microarrays was assessed by the R package in Bioconductor—arrayQualityMetrics. The microarray data with good quality were normalized and preprocessed by the robust multiarray average (RMA) package and annotated by converting different probe IDs to ENTREZ IDs and gene symbols with the use of the annotation packages in the Bioconductor. “The highest interquartile range” method was performed when multiple probes matched with a unique official gene ID. Genes with low mean expression levels across most arrays were filtered out prior to the subsequent meta-analysis.

Meta-analysis of the expression datasets after the above preparation was performed by NetworkAnalyst [32]. In this case, three microarray datasets were from the same platform and of similar quality. Hence, we used Stouffer's method to combine the *p* values from individual studies. The combined *p* value cutoff level was set at 0.05.

## 3. Results

### 3.1. Research Strategy

**Figure 1:**
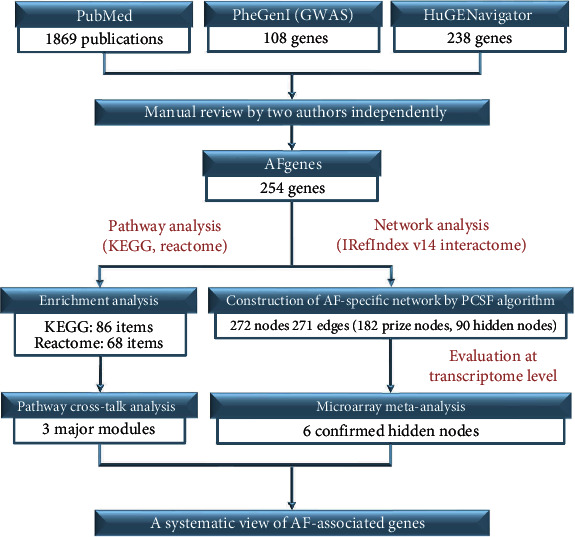
Flow diagram of the integrative analysis. We retrieved all genes associated with atrial fibrillation (AF) from various databases. These genes were reviewed by the authors to exclude insignificant genes. The filtered genes were used for pathway analysis (pathway enrichment analysis and pathway crosstalk analysis) and to construct the AF-specific subnetwork under the human interactome for the inference of potential AF-associated genes. These potential genes were evaluated by means of microarray meta-analysis (prize nodes, the known AF-associated genes. Hidden nodes, the potential AF-associated genes inferred by the known AF-associated genes in the AF-specific subnetwork. PCSF, prize-collecting Steiner forest).

We designed and implemented an integrative analysis based on pathway and network analysis to explore the genetic basis of AF-associated genes and detect novel AF-associated genes for further genetic association studies. Our analysis was divided into four steps (Figure 1). First, we carefully collected reliable genes associated with AF from various databases. Second, we performed pathway analyses for these genes to gain better insight into the potential biological mechanisms. Third, by use of the PCSF algorithm, we constructed an AF-specific subnetwork with the genes associated with AF while inferring some novel genes potentially associated with AF. Finally, we performed a microarray meta-analysis to determine which novel genes deserved further genetic association studies.

### 3.2. AF-Associated Gene Identification From Genetic Association Studies in Three Databases

There were 1869 publications reporting one or more genes that had a significant relationship with AF, and 131 significant genes were extracted. Moreover, 238 and 108 genes associated with AF were retrieved from the HuGE Navigation and the PheGenI, respectively. After reviewing and merging genes from the three databases, we compiled a list of 254 AF-associated genes (AFgenes) for subsequent analyses (Table S1). Among them, there were multiple genes encoding ion channels, such as sodium voltage-gated channel genes (e.g., *SCN3A*, *SCN5A*, *SCN10A*, *SCN1B*, *SCN2B*, *SCN3B*, and *SCN4B*), potassium voltage-gated channel genes (e.g., *KCNQ1*, *KCNE1*, *KCNE2*, *KCNE3*, *KCNE4*, and *KCNE5*), and transient receptor potential cation channel subfamily C genes (e.g., *TRPC1* and *TRPC3*). Additionally, there were genes encoding subunit A of glutamy-tRNA aminotransferase (e.g., *GATA4*, *GATA5*, and *GATA6*), solute carrier family genes (e.g., *SLC25A20* and *SLC22A25*), paired-like homeodomain transcription factor 2 (*PITX2*), and other genes involved in the immune response, such as *IL-18*, *IL-27*, *IL-6R*, and *IL-1RN*. These genes were diverse and in small clusters, which demonstrated the complexity of AF and the indispensable role of ion channels in the initiation and development of AF.

### 3.3. Immuno-Endocrine-Neuronal Pathways Connecting Various Diseases to AF Occurrence

The pathway enrichment analysis identified 154 significant overrepresentative pathways (adjusted *p* < 0.05, Table S2), 22 of which contained more than 10 genes overlapping the AFgenes. These top pathways were associated with cardiac electromechanical activity (e.g., cardiac conduction, Phase 0—rapid depolarization, the calcium signaling pathway, and cardiac muscle contraction) and cardiomyopathy (e.g., hypertrophic cardiomyopathy and dilated cardiomyopathy).

**Figure 2:**
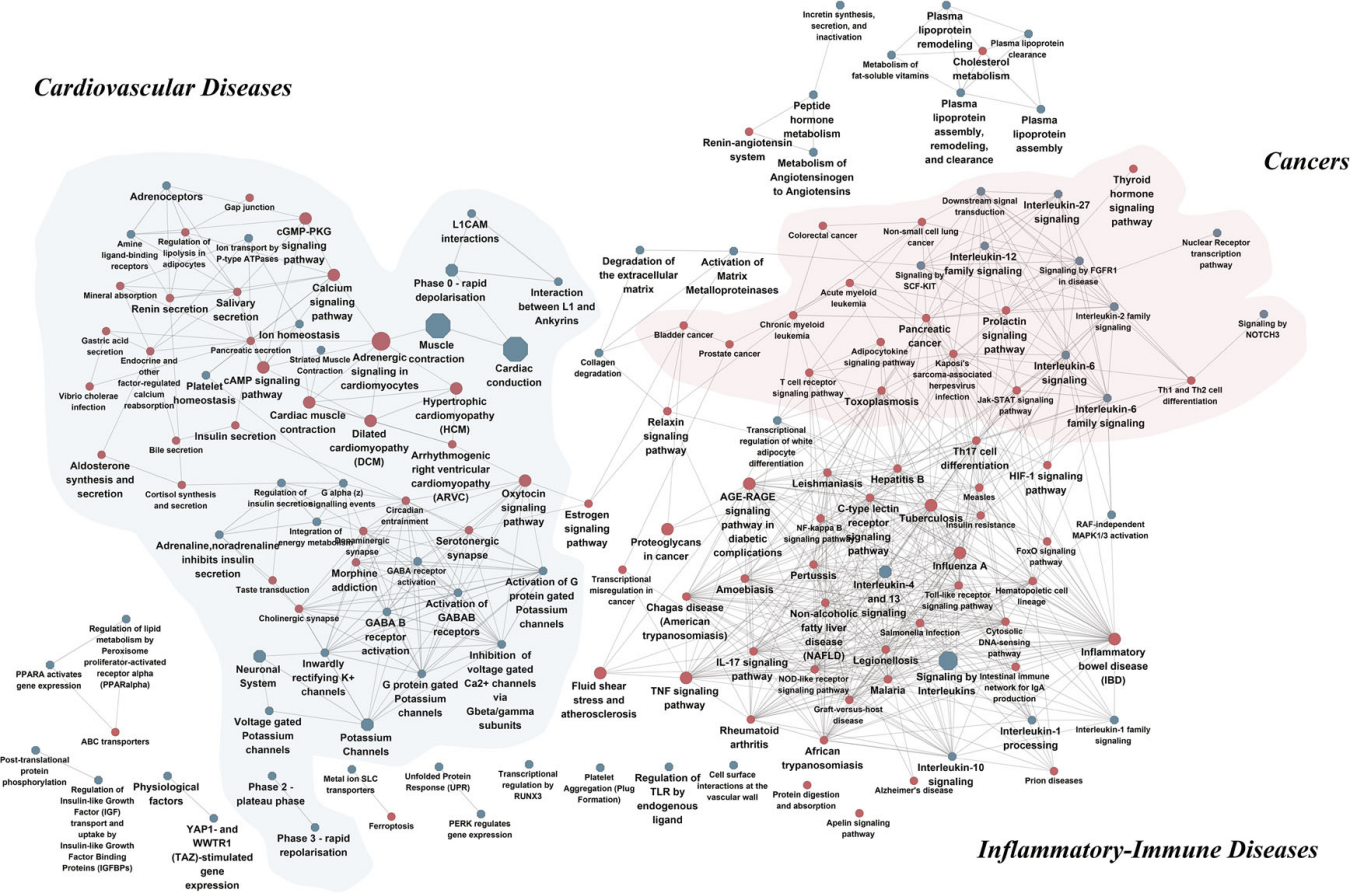
Pathway crosstalk of AFgenes. In this figure, each node represents a significant pathway, and each edge represents pathway crosstalk. The size of each node is approximately proportional to the number of AF-associated genes in the corresponding pathway. The width of each edge is approximately proportional to the kappa score.

As shown in Figure 2, significant pathways with kappa scores above 0.4 were roughly divided into three modules. The left side of the figure shows a large module dominated by cardiovascular diseases with related neural-endocrine pathways. These pathways (e.g., adrenergic signaling in cardiomyocytes, the cGMP-PKG signaling pathway, and aldosterone synthesis and secretion) play critical roles in regulating cardiac electromechanical activity, especially the activity of ion channels. The right side of the figure shows a module consisting of the pathways associated with various cancers (e.g., colorectal cancer, non–small cell lung cancer, and myeloid leukemia). The bottom of the figure shows a module including inflammatory-immune diseases (e.g., rheumatoid arthritis [RA], nonalcoholic fatty liver disease, and inflammatory bowel disease [IBD]) with the associated pathways, such as signaling by interleukins, the NOD-like receptor signaling pathway, and the intestinal immune network for IgA production.

Importantly, rather than being independent, the modules were connected with each other by some pathways, such as the estrogen signaling pathway.

### 3.4. Extraction of the AF-Specific Subnetwork

**Figure 3:**
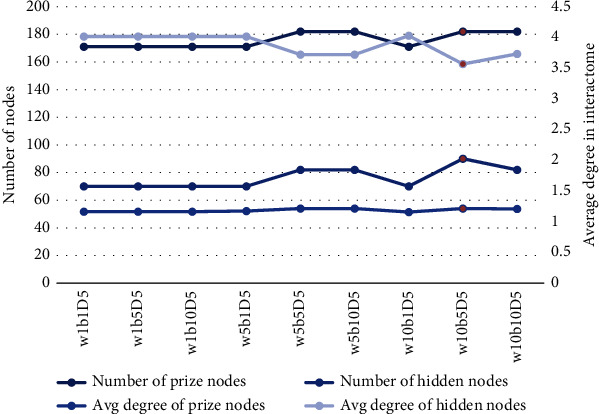
An analysis of various parameter sets when running Forest on the AFgenes. A good choice of parameter set is indicated by the red circle.

**Figure 4:**
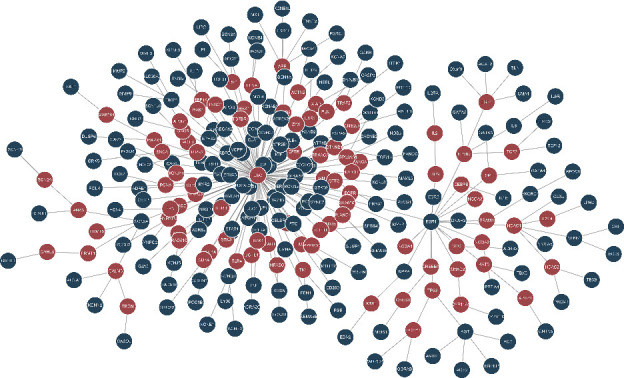
AF-specific protein–protein interaction subnetwork constructed by the prize-collecting Steiner forest algorithm, containing 272 nodes and 271 edges. Blue circular vertices, genes of AFgenes (the known AF-associated genes); red circular vertices, expanding genes (the potential AF-associated genes).

Among the 254 AFgenes, 183 were mapped to the human interactome. A set of parameters (*w* = 10, *b* = 5, and *D* = 5) was selected for the inference of the AF-specific subnetwork. As shown in Figure 3, this set of parameters minimized the difference between the average degree of prized and hidden nodes (1.21 and 3.57) and included the largest number of prize nodes (182). With the use of the PCSF algorithm, we successfully constructed an AF-specific subnetwork that contained 182 prized and 90 hidden nodes as well as 271 edges (highly reliable protein–protein interactions) (Figure 4). Most nodes (269/271, 99.3%) in the AF-specific subnetwork had relatively high “FractionOfOptimalForestsContaining” values (> 0.9) in the noisyEdges run, and 234 of 271 nodes had extremely low “FractionOfOptimalForestsContaining” values (< 0.1) in the randomTerminals run (Table S3). These results suggested that this subnetwork was a reliable and specific subnetwork for AF. For the hidden genes, 24 of the 90 genes (e.g., *APLP1*, *HUS1*, *CREB1*, and *PRMT1*) were extremely robust and specific for the AF-specific subnetwork and deserved the following assessment by microarray meta-analysis. In the “FractionOfOptimalForestsContaining” analysis, these 24 genes were given one point each in the noisyEdges run but assigned zero points each in the randomTerminals run (Table S3).

### 3.5. Assessment of Hidden Genes by Meta-Analysis of Microarray Data

Three datasets (accession numbers: GSE2240, GSE14975, and GSE41177) were downloaded from the GEO. They contained microarray data from 28 patients with sinus rhythm and 31 patients with AF. Notably, all of the datasets were generated with the use of the Affymetrix microarray platform. The quality evaluation of these microarray datasets suggested that they all were suitable for the subsequent meta-analysis (File S1). We conducted a meta-analysis across three datasets and identified a total of 1245 DEGs under the significance threshold of a combined *p* value < 0.05 (Table S4). When we restricted the analysis to the 24 robust and specific hidden genes (novel genes potentially associated with AF), six genes were demonstrated to be differentially expressed in the patients with AF and sinus rhythm, namely, *APLP1*, *CREB1*, *CREBBP*, *PRMT1*, *IRAK1*, and *PLXND1* (Table 1). These genes may play roles in AF susceptibility and be novel targets for further exploration.

## 4. Discussion

A spurt of progress in genetic technology has resulted in a better understanding of the role of genetic factors in the occurrence of AF since GWAS was first applied to AF in 2007. However, translating gene variations into knowledge of AF mechanisms remains difficult. Moreover, the established genetic variants contribute modestly to AF variance, implying that substantial additional genetic variations do not reach the genome-wide significance thresholds. Believing that more and larger studies should be performed to detect additional variations is not difficult. However, is bigger always better? Moreover, is there a way to narrow down the candidate risk locus search based on the known AF-associated genes [33]? Under such circumstances, inspired by recent studies on Alzheimer's disease and coronary artery disease [11, 13], we performed a comprehensive pathway and network-based analysis of AF-associated genes. Incorporating previous knowledge about gene and protein function with the known AF-associated genes, our study explored the interconnection of these genes and the underlying biological meanings by pathway analyses, and by means of the PCSF algorithm, this study further inferred novel potential AF-associated genes that have strong functional associations with the known AF-associated genes. After evaluating these novel genes with a microarray meta-analysis at the transcriptome level, we observed that six novel genes (*APLP1*, *CREB1*, *CREBBP*, *PRMT1*, *IRAK1*, and *PLXND1*) that were potentially associated with AF deserved further genetic association studies in the future.

**Figure 5:**
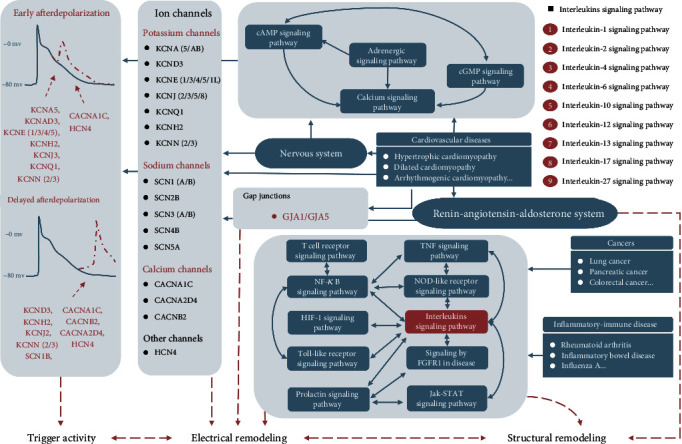
Main biological pathways associated with the AFgenes. AF might share a common genetic basis and biological process with tumors and inflammatory diseases in addition to cardiovascular diseases. Numerous neuro-endocrine-immune pathways connect these diseases with AF.

When the results from the pathway analyses and the prior biological knowledge base were integrated, the major pathways associated with the AFgenes could be summarized in a diagram (Figure 5). Previous cell and/or animal experiments have proved that alterations in ion channels can promote the initiation, maintenance, and progression of AF via triggered activity and a reentry mechanism [34, 35]. Similarly, our pathway enrichment analysis revealed that the top enrichment pathways were associated with ion channels, such as the calcium signaling pathway. This result highlights that AF, especially genetic AF, is more likely to be an ion channel disease. More importantly, the pathway crosstalk analysis suggests that AF is not only an isolated disease with abnormal electrophysiological activity but may also be a manifestation of various pathologies in addition to cardiac diseases. To our surprise, numerous pathway items that were associated with cancers (e.g., breast cancers, colorectal cancer, and small cell lung cancer) were enriched among the AFgenes. This implies that AF and cancer may have a tight relationship. Cancer can increase the risk of AF by approximately 30%, a fact that has been largely ignored by many clinicians [36]. Although the potential mechanisms of AF induction in cancers are still far from being fully understood, there are some potential pathogenetic links between these diseases. The most common shared genes (NFKB1, TCF7L2, TMPRSS2, CASP3, STAT3, TGFB1, MMP9, POLK, and RAF1) could become the most likely candidate genes for future research into the mechanisms of cardiooncology. Moreover, as shown in the crosstalk map, AF and cancers share various interlinked inflammatory and immune pathways (e.g., interleukin-2 family signaling, interleukin-6 signaling, interleukin-12 family signaling, and Th1 and Th2 cell differentiation), which is consistent with previous studies indicating that AF might represent an inflammatory complication of malignancies [37–39]. Inflammation may be a potent trigger of AF, whereas AF can create and sustain the inflammatory environment [40, 41]. In the pathway analysis, inflammatory-immune disease (e.g., IBD, influenza, and RA) and the related pathways (e.g., the IL-17 signaling pathway, the NOD-like receptor signaling pathway, interleukin-4 and interleukin-13 signaling, and signaling by interleukins) were enriched among the AFgenes. This result implied that inflammatory-immune disease could increase the risk of occurrence of AF. Coincidentally, a large-scale case-control study revealed that influenza was significantly associated with the development of AF, with an 18% increase in risk [41]. A recent meta-analysis and animal experiments demonstrated that RA was associated with an increased risk of AF due to chronic inflammation [42, 43]. A nationwide study including 24,499 patients with new-onset IBD and 236,275 age- and sex-matched controls established that active IBD was associated with the increased risk of AF [44]. The association of nonalcoholic fatty liver disease with AF also has been demonstrated by recent studies [45]. Moreover, previous studies have demonstrated that various cardiovascular disorders can increase the risk of AF via multiple pathophysiological processes [46, 47]. Based on the common pathway theory, the shared AFgenes with influenza (CCL2, IL18, IL1B, IL6, MX1, NFKB1, RAF1, STAT1, TMPRSS2, and TNF), RA (CCL2, IL18, IL1B, IL6, MMP3, TGFB1, and TNF), and IBD (IL10, IL18, IL1B, IL6, NFKB1, NOD2, STAT1, STAT3, TGFB1, and TNF) may serve as entry points for future research on disease comorbidity mechanisms.

However, we may be confused as to whether AF is only influenced by cardiomyopathy when glancing at the map. Several factors might account for the cluster of cardiomyopathy pathways: (1) our study collected data primarily from genetic studies; (2) many cardiomyopathies are regarded as monogenic disorders and are more largely influenced by genetic factors than other cardiovascular disorders, such as hypertension and coronary artery disease [48]; and (3) AF is more common in patients with cardiomyopathy than in the general population [49–52]. Although cardiovascular disorders other than cardiomyopathy did not seem to be prominently represented in the pathway crosstalk, the shared pathways of cardiovascular disorders and AF have been represented in the crosstalk (e.g., renin–angiotensin system, neuronal system, aldosterone synthesis and secretion, and insulin secretion). These pathways are primarily involved in three neuroendocrine systems, namely, the autonomic nervous system (ANS), renin–angiotensin–aldosterone system (RAAS), and endocrine system, which is consistent with the findings of previous studies [53–58]. The pathway analysis of the AFgenes might provide reliable clues about the clinical risk factors of AF. Additionally, the analysis might provide causal hints for AF.

Furthermore, we inferred six novel genes that were potentially associated with AF from the network analysis and microarray meta-analysis. Recent studies have identified the functional data for the link between these genes and AF. *APL1*, amyloid-like protein 1, appears in the AF-specific subnetwork. *APL1* participates in the perturbation of cyclic nucleotide metabolism during the left atrial endocardial remodeling induced by AF [59]. *CREB1* encodes cAMP-responsive element-binding protein (CREB). Experimental studies showed that Angiotensin II exerts a fibrillatory effect on HL-1 cells via PKC and the CREB-dependent calcium signaling pathway [60]. Similarly, the inhibition of CREB activity by thyroid hormone also results in AF via altered intracellular calcium signaling [61]. *CREBBP* is the encoding gene of the CREB-binding protein. A previous study shows that *CREBBP* is associated with the left atrial diameter and arrhythmia recurrence after AF catheter ablation via the calcium signaling pathway [62]. In addition, *CREBBP* is essential for angiogenesis and cardiac development. Its mutations can cause Rubinstein–Taybi syndrome, which is characterized by congenital cardiac defects [63]. *PLXND1* (PlexinD1) and semaphorin signaling are essential for cardiovascular development, and their interruption in mice can result in CHD and vascular patterning defects [64]. A recent genome-wide study of AF highlighted that cardiac development acts as a critical factor in the occurrence of AF [65]. Hence, the harmful influence of *CREBBP* and *PLXND1* on cardiac development might lead to AF pathogenesis. The application of the canine AF model reveals that the *DDAH–PRMT–ADMA* system likely plays a pivotal role in regulating endothelial function in AF [66]. Although no functional evidence has suggested that *IRAK1* is directly associated with AF, *IRAK1* interacts with IL-10 in the AF-specific subnetwork. Recent animal studies have shown that the genetic deletion of *IL-10* exacerbates obesity-induced atrial inflammation, fibrosis, and fibrillation and that *IL-10* therapy can inhibit this pathology [67]. Therefore, *IRAK1* might be involved in the occurrence of AF via inflammatory signaling pathways. Taken together, these six novel genes inferred by our network analysis based on the AFgenes might help in discovering novel risk loci of AF. Although these genes have been less understood in previous genetic association studies of AF, the functional and transcriptomic data have implied their roles in the biological processes of AF, which makes these genes worth validating in further genetic association studies and experimental studies.

### 4.1. Limitations

Undoubtedly, there will be something that we can still improve. Our results largely depend on the established AF-associated genes and public databases. The bias in the original studies also appears in our study. Databases are constantly being updated. The insignificant genes were not recruited in our study to avoid false-positive errors. However, with the expansion of the sample size, some of the genes might be significant. Moreover, our findings need further validation in the future.

## 5. Conclusion

In this study, a comprehensive biological framework was applied to explore the molecular networks and signaling pathways underlying AF based on the genes associated with AF. Our analysis suggested that AF was not only an ion channel disease but also associated with tumors and inflammatory-immune disorders in addition to cardiovascular diseases. These associations may be investigated in future studies through the shared neuroendocrine-immune pathways, such as signaling mediated by interleukins. From the network analysis, we also inferred six potential AF-associated genes that merited further genetic association studies. Taken together, this integrative analysis might provide valuable clues toward discovering the clinical risk factors of AF and having a better understanding of the molecular mechanisms underlying AF. However, these findings from comprehensive in silico analysis necessitate additional experimental and prospective studies in the future.

## Figures and Tables

**Table 1:** Potential AF-associated genes meriting further genetic association studies.

**Gene name**	**Protein name**	**Meta-analysis ** **p** ** value**	**Network analysis**			
			**Prize node**	**Hidden node**	**FractionOfOptimalForestsContaining (noisyEdges)**	**FractionOfOptimalForestsContaining (randomTerminals)**
APLP1	Amyloid-like protein 1	0.017	×	✓	1	0
CREB1	Cyclic AMP-responsive element-binding protein 1	0.002	×	✓	1	0
CREBBP	CREB-binding protein	0.024	×	✓	1	0
PRMT1	Protein arginine N-methyltransferase 1	0.001	×	✓	1	0
IRAK1	Interleukin-1 receptor–associated kinase 1	0.017	×	✓	1	0
PLXND1	Plexin-D1	0.035	×	✓	1	0

## Data Availability

We have provided the data in the Supporting Information.

## Supporting Information

Supporting Information Additional supporting information can be found online in the Supporting Information section. Table S1. A list of reliable AF-associated genes from genetic association studies (AFgenes). Table S2. Pathways enriched in AFgenes. Table S3. Assessment of AF-specific subnetwork constructed by the prize-collecting Steiner forest algorithm. Table S4. Significant genes in the microarray meta-analysis of AF. File S1. Quality assessment of the microarray of AF downloaded from the Gene Expression Omnibus.
