# Supplementary material for: A Network and Pathway Analysis of Genes Associated With Atrial Fibrillation
Source: Cardiovasc Ther. 2024 Oct 5;2024:7054039. doi: 10.1155/2024/7054039 (PMC11470814; doi:10.1155/2024/7054039)
Supplement: Supporting Information — Additional supporting information can be found online in the Supporting Information section. Table S1. A list of reliable AF-associated genes from genetic association studies (AFgenes). Table S2. Pathways enriched in AFgenes. Table S3. Assessment of AF-specific subnetwork constructed by the prize-collecting Steiner forest algorithm. Table S4. Significant genes in the microarray meta-analysis of AF. File S1. Quality assessment of the microarray of AF downloaded from the Gene Expression Omnibus. [file 7054039.f1.zip › Supplement material.pdf]

SUPPLEMENTAL MATERIAL

Supplement Table 1 A list of reliable AF-associated genes from genetic association studies (AFgenes)

IL27	IL1RN	KCNE2	APOB	RNA5SP215	DUSP6	ACE	SLN	CDKN2B
IL18	GATA6	KCNJ2	TDRD15	FERD3L	POC1B	IL6	MEIS1	ADRA2C
PPFIA4	GJA1	NPPA	RNA5SP87	TWISTNB	ATP2B1	AGTR1	ZC3HC1	IL2RA
SLC1A4	TIMP2	CAV2	SLC30A3	BAZ1B	ZCCHC8	FGB	TIMP1	RAF1
HAND2	KCNJ5	IL6R	FTH1P3	SEMA3E	LRRC63	CYP2D6	KCNN2	CHI3L1
NEBL	AT1R	ESR1	NRBP1	RAD23BP2	RAD51B	MYH7	HSPA5	STK38
SH3PXD2A	NOS1	GATA5	GCKR	DNAJB9	ITPK1	MMP9	STAT1	OR9K1P
GJA5	KCNE1	ACE2	WDPCP	C7orf66	TCF12	KCNE1L	SCN1A	CMA1
AGXT2	KCND3	KCNE4	RPL6P5	LPL	LIPC	SCN3B	LOC100128922	LOC729065
CACNA1C	KCNA5	GATA4	RNU7-2P	RPL30P9	LIPC-AS1	IL1B	WNT8A	FSD1L
SCN2B	SHOX2	CYP11B2	ABCA12	LY96	RPLP1	LMNA	ADRB3	MTHFR
M235T	TBX5	KCNJ8	CD200	TRIB1	GEMIN8P1	COX8A	MYL4	SYNPO2L
NOS3	ADH1B	CRP	SEMA5B	LINC00861	RBFOX1	IL10	KCNJ3	CNTN5
LKB1	TNNI3	AGT	SPSB4	LSM1P1	FTO	ADRB2	MIR146A	ESR2
ATP2A2	VKORC1	SOX5	PCDH7	RPL31P42	HERPUD1	GRK5	KCNAB2	
ENPEP	UTS2	ADRB1	MAPRE1P2	UBA52P6	DNAH2	TNNT2	SCN3A	
CACNB2	KCNQ1	ABCA1	USO1	DMRTA1	THEMIS3P	SCN4B	MIR92B	
CACNA2D4	FANCC	GNB3	SLC39A8	CHCHD2P9	AKR1B1P6	TGFB1	TBX3	
PITX2	MYOZ1	ZFHX3	NFKB1	TLE4	SUGP1	HMOX1	PSRC1	
KCNE5	ABCB4	CAV1	MIR297	ARL5B	BCL3	KCNE3	EDN2	
SCN1B	CASP3	KCNN3	MAML3	AIFM1P1	APOC1	CCL2	MIF	
N588K	KCNH2	SYNE2	SUMO2P5	AGAP5	PLCG1	TNF	C6orf204	
L532P	PDE4D	C9orf3	HMGCR	TCF7L2	RPL23AP81	CASQ2	OR4Q3	
MINK	SCN10A	HCN4	COL4A3BP	FADS1	LPIN3	MMP2	STAT3	
HO1	SCN5A	PRRX1	POLK	ZPR1	SULF2	MMP3	CORIN	
B1B1	NKX2-6	CETP	POC5	SIK3	SRMP1	RYR2	TSEN15	
GREM2	NEURL	CELSR2	CYP21A2	BSX	MX1	EPHX2	COL1A1	
AT1	GJA1	GORAB	RPL35P3	RPL34P23	TMPRSS2	CP	VOPP1	
GNAS1	CAND2	HMCN1	PRDM1	TEAD4	VENTXP1	FBN1	ATP1B1	
NKX2-5	CUX2	GRHL1	MIR3144	RPL13AP24	RPL7P58	JPH2	NOD2	

Supplement Table 2 Pathways enriched in AFgenes

	Term	% Associated Genes	Nr. Genes	Term PValue	Term PValue Corrected with Benjamini-Hochberg	Associated Genes Found
KEGG	Intestinal immune network for IgA production	6.12	3	3.35E-02	3.87E-02	[IL10, IL6, TGFB1]
	Protein digestion and absorption	5.56	5	9.66E-03	1.43E-02	[ACE2, ATP1B1, COL1A1, KCNE3, KCNQ1]
	Circadian entrainment	6.25	6	2.62E-03	5.14E-03	[CACNA1C, GNAS, GNB3, KCNJ3, KCNJ5, RYR2]
	Inflammatory bowel disease (IBD)	15.38	10	2.70E-08	3.26E-07	[IL10, IL18, IL1B, IL6, NFKB1, NOD2, STAT1, STAT3, TGFB1, TNF]
	NF-kappa B signaling pathway	5.26	5	1.20E-02	1.70E-02	[IL1B, LY96, NFKB1, PLCG1, TNF]
	Bile secretion	7.04	5	3.56E-03	6.50E-03	[ABCB4, ATP1B1, GNAS, HMGCR, KCNN2]
	Cholinergic synapse	4.46	5	2.29E-02	2.77E-02	[CACNA1C, GNB3, KCNJ2, KCNJ3, KCNQ1]
	Tuberculosis	6.15	11	5.56E-05	2.36E-04	[CASP3, IL10, IL18, IL1B, IL6, NFKB1, NOD2, RAF1, STAT1, TGFB1, TNF]
	HIF-1 signaling pathway	9.00	9	1.35E-05	6.84E-05	[HMOX1, IL6, IL6R, NFKB1, NOS3, NPPA, PLCG1, STAT3, TIMP1]
	Mineral absorption	5.88	3	3.71E-02	4.19E-02	[ATP1B1, ATP2B1, HMOX1]
	Serotonergic synapse	7.96	9	3.61E-05	1.58E-04	[CACNA1C, CASP3, CYP2D6, GNAS, GNB3, KCNJ3, KCNJ5, KCNN2, RAF1]
	Cholesterol metabolism	12.00	6	7.74E-05	2.83E-04	[ABCA1, APOB, APOC1, CETP, LIPC, LPL]
	FoxO signaling pathway	4.55	6	1.21E-02	1.70E-02	[IL10, IL6, RAF1, STAT3, STK11, TGFB1]
	Dopaminergic synapse	4.58	6	1.17E-02	1.69E-02	[CACNA1C, GNAS, GNB3, KCNJ3, KCNJ5, SCN1A]
	Alzheimer's disease	4.09	7	1.19E-02	1.71E-02	[ATP2A2, CACNA1C, CASP3, COX8A, IL1B, LPL, TNF]
	Ferroptosis	7.50	3	1.97E-02	2.44E-02	[CP, HMOX1, SLC39A8]
	Rheumatoid arthritis	7.78	7	3.14E-04	8.64E-04	[CCL2, IL18, IL1B, IL6, MMP3, TGFB1, TNF]
	Taste transduction	4.82	4	3.19E-02	3.70E-02	[CACNA1C, GNB3, HCN4, SCN3A]
	Prion diseases	8.57	3	1.38E-02	1.85E-02	[HSPA5, IL1B, IL6]
	Hepatitis B	6.25	9	2.35E-04	6.59E-04	[CASP3, IL6, MMP9, NFKB1, RAF1, STAT1, STAT3, TGFB1, TNF]
	Cardiac muscle contraction	14.10	11	1.37E-08	1.79E-07	[ATP1B1, ATP2A2, CACNA1C, CACNA2D4, CACNB2, COX8A, MYH7, MYL4, RYR2, TNNI3, TNNT2]
	Insulin secretion	7.06	6	1.41E-03	3.20E-03	[ATP1B1, CACNA1C, GNAS, KCNN2, KCNN3, RYR2]
	Morphine addiction	6.59	6	2.00E-03	4.18E-03	[GNAS, GNB3, GRK5, KCNJ3, KCNJ5, PDE4D]
	Estrogen signaling pathway	6.57	9	1.62E-04	4.78E-04	[ESR1, ESR2, GNAS, KCNJ3, KCNJ5, MMP2, MMP9, NOS3, RAF1]
	Adrenergic signaling in cardiomyocytes	13.89	20	1.60E-14	8.37E-13	[ADRB1, ADRB2, AGTR1, ATP1B1, ATP2A2, ATP2B1, CACNA1C, CACNA2D4, CACNB2, GNAS, KCNE1, KCNQ1, MYH7, MYL4, RYR2, SCN1B, SCN4B, SCN5A, TNNI3, TNNT2]
	Vibrio cholerae infection	6.00	3	3.53E-02	4.04E-02	[GNAS, KCNQ1, PLCG1]
	Graft-versus-host disease	7.32	3	2.11E-02	2.56E-02	[IL1B, IL6, TNF]
	Salmonella infection	4.65	4	3.56E-02	4.05E-02	[IL18, IL1B, IL6, NFKB1]
	Prolactin signaling pathway	8.57	6	5.04E-04	1.26E-03	[ESR1, ESR2, NFKB1, RAF1, STAT1, STAT3]
	Measles	5.22	7	3.25E-03	6.08E-03	[IL1B, IL2RA, IL6, MX1, NFKB1, STAT1, STAT3]
	Thyroid hormone signaling pathway	6.03	7	1.43E-03	3.17E-03	[ATP1B1, ATP2A2, ESR1, GATA4, PLCG1, RAF1, STAT1]
	Pertussis	9.21	7	1.09E-04	3.56E-04	[CASP3, IL10, IL1B, IL6, LY96, NFKB1, TNF]
	Adipocytokine signaling pathway	5.80	4	1.75E-02	2.21E-02	[NFKB1, STAT3, STK11, TNF]
	Apelin signaling pathway	4.38	6	1.44E-02	1.91E-02	[AGTR1, GNB3, MYL4, NOS3, RAF1, RYR2]
	Hypertrophic cardiomyopathy (HCM)	15.66	13	1.63E-10	3.19E-09	[ACE, ATP2A2, CACNA1C, CACNA2D4, CACNB2, IL6, LMNA, MYH7, RYR2, TGFB1, TNF, TNNI3, TNNT2]
	Oxytocin signaling pathway	6.54	10	7.28E-05	2.79E-04	[CACNA1C, CACNA2D4, CACNB2, GNAS, KCNJ2, KCNJ3, KCNJ5, NOS3, RAF1, RYR2]
	Gap junction	4.55	4	3.83E-02	4.29E-02	[ADRB1, GJA1, GNAS, RAF1]
	Influenza A	5.78	10	2.01E-04	5.85E-04	[CCL2, IL18, IL1B, IL6, MX1, NFKB1, RAF1, STAT1, TMPRSS2, TNF]
	Renin-angiotensin system	26.09	6	6.79E-07	5.08E-06	[ACE, ACE2, AGT, AGTR1, CMA1, ENPEP]
	Legionellosis	10.91	6	1.33E-04	4.18E-04	[CASP3, IL18, IL1B, IL6, NFKB1, TNF]
	Arrhythmogenic right ventricular cardiomyopathy (ARVC)	11.11	8	8.67E-06	4.86E-05	[ATP2A2, CACNA1C, CACNA2D4, CACNB2, GJA1, LMNA, RYR2, TCF7L2]
	Regulation of lipolysis in adipocytes	7.41	4	7.54E-03	1.20E-02	[ADRB1, ADRB2, ADRB3, GNAS]
	Toll-like receptor signaling pathway	5.77	6	3.90E-03	6.96E-03	[IL1B, IL6, LY96, NFKB1, STAT1, TNF]
	Renin secretion	13.85	9	3.50E-07	2.89E-06	[ACE, ADRB1, ADRB2, ADRB3, AGT, AGTR1, CACNA1C, GNAS, KCNJ2]
	Dilated cardiomyopathy (DCM)	14.44	13	4.63E-10	8.07E-09	[ADRB1, ATP2A2, CACNA1C, CACNA2D4, CACNB2, GNAS, LMNA, MYH7, RYR2, TGFB1, TNF, TNNI3, TNNT2]
	NOD-like receptor signaling pathway	4.76	8	3.00E-03	5.74E-03	[CCL2, IL18, IL1B, IL6, NFKB1, NOD2, STAT1, TNF]
	Leishmaniasis	8.11	6	6.79E-04	1.64E-03	[IL10, IL1B, NFKB1, STAT1, TGFB1, TNF]
	Aldosterone synthesis and secretion	8.33	8	7.17E-05	2.81E-04	[AGTR1, ATP1B1, ATP2B1, CACNA1C, CYP11B2, CYP21A2, GNAS, KCNJ5]
	Kaposi's sarcoma-associated herpesvirus infection	4.30	8	5.53E-03	9.34E-03	[CASP3, GNB3, IL6, NFKB1, PLCG1, RAF1, STAT1, STAT3]
	Cytosolic DNA-sensing pathway	6.25	4	1.36E-02	1.85E-02	[IL18, IL1B, IL6, NFKB1]
	Relaxin signaling pathway	6.92	9	1.08E-04	3.61E-04	[COL1A1, GNAS, GNB3, MMP2, MMP9, NFKB1, NOS3, RAF1, TGFB1]
	Fluid shear stress and atherosclerosis	7.19	10	3.21E-05	1.44E-04	[CAV1, CAV2, CCL2, HMOX1, IL1B, MMP2, MMP9, NFKB1, NOS3, TNF]
	Chagas disease (American trypanosomiasis)	8.82	9	1.59E-05	7.55E-05	[ACE, CCL2, GNAS, IL10, IL1B, IL6, NFKB1, TGFB1, TNF]
	C-type lectin receptor signaling pathway	7.69	8	1.26E-04	4.05E-04	[BCL3, IL10, IL1B, IL6, NFKB1, RAF1, STAT1, TNF]
	Cortisol synthesis and secretion	6.35	4	1.29E-02	1.79E-02	[AGTR1, CACNA1C, CYP21A2, GNAS]
	Transcriptional misregulation in cancer	4.30	8	5.53E-03	9.34E-03	[DUSP6, IL6, MEIS1, MMP3, MMP9, NFKB1, POLK, TMPRSS2]
	Insulin resistance	4.67	5	1.92E-02	2.40E-02	[IL6, NFKB1, NOS3, STAT3, TNF]
	Jak-STAT signaling pathway	4.32	7	9.04E-03	1.38E-02	[IL10, IL2RA, IL6, IL6R, RAF1, STAT1, STAT3]
	Proteoglycans in cancer	5.47	11	1.57E-04	4.74E-04	[CASP3, CAV1, CAV2, ESR1, MMP2, MMP9, PLCG1, RAF1, STAT3, TGFB1, TNF]
	African trypanosomiasis	14.29	5	1.35E-04	4.14E-04	[IL10, IL18, IL1B, IL6, TNF]
	Non-alcoholic fatty liver disease (NAFLD)	5.37	8	1.41E-03	3.17E-03	[CASP3, COX8A, IL1B, IL6, IL6R, NFKB1, TGFB1, TNF]
	Hematopoietic cell lineage	5.15	5	1.31E-02	1.80E-02	[IL1B, IL2RA, IL6, IL6R, TNF]
	Colorectal cancer	5.81	5	8.01E-03	1.26E-02	[CASP3, POLK, RAF1, TCF7L2, TGFB1]
	IL-17 signaling pathway	8.60	8	5.71E-05	2.36E-04	[CASP3, CCL2, IL1B, IL6, MMP3, MMP9, NFKB1, TNF]
	ABC transporters	6.67	3	2.69E-02	3.17E-02	[ABCA1, ABCA12, ABCB4]
	Pancreatic cancer	8.00	6	7.29E-04	1.73E-03	[NFKB1, POLK, RAF1, STAT1, STAT3, TGFB1]
	Malaria	14.29	7	5.92E-06	3.44E-05	[CCL2, IL10, IL18, IL1B, IL6, TGFB1, TNF]
	AGE-RAGE signaling pathway in diabetic complications	14.14	14	1.29E-10	2.90E-09	[AGTR1, CASP3, CCL2, COL1A1, IL1B, IL6, MMP2, NFKB1, NOS3, PLCG1, STAT1, STAT3, TGFB1, TNF]
	Calcium signaling pathway	6.04	11	6.47E-05	2.60E-04	[ADRB1, ADRB2, ADRB3, AGTR1, ATP2A2, ATP2B1, CACNA1C, GNAS, NOS3, PLCG1, RYR2]
	Th1 and Th2 cell differentiation	5.43	5	1.06E-02	1.55E-02	[IL2RA, MAML3, NFKB1, PLCG1, STAT1]
	Prostate cancer	6.19	6	2.76E-03	5.35E-03	[MMP3, MMP9, NFKB1, RAF1, TCF7L2, TMPRSS2]
	Th17 cell differentiation	8.41	9	2.34E-05	1.08E-04	[IL1B, IL2RA, IL6, IL6R, NFKB1, PLCG1, STAT1, STAT3, TGFB1]
	Endocrine and other factor-regulated calcium reabsorption	8.51	4	4.60E-03	7.93E-03	[ATP1B1, ATP2B1, ESR1, GNAS]
	Toxoplasmosis	7.08	8	2.25E-04	6.42E-04	[CASP3, IL10, LY96, NFKB1, STAT1, STAT3, TGFB1, TNF]
	Bladder cancer	7.32	3	2.11E-02	2.56E-02	[MMP2, MMP9, RAF1]
	cGMP-PKG signaling pathway	8.59	14	9.25E-08	9.07E-07	[ADRA2C, ADRB1, ADRB2, ADRB3, AGTR1, ATP1B1, ATP2A2, ATP2B1, CACNA1C, GATA4, KCNJ8, MYH7, NOS3, RAF1]
	Salivary secretion	6.67	6	1.89E-03	4.01E-03	[ADRB1, ADRB2, ADRB3, ATP1B1, ATP2B1, GNAS]
	Chronic myeloid leukemia	5.26	4	2.40E-02	2.88E-02	[NFKB1, POLK, RAF1, TGFB1]
	T cell receptor signaling pathway	4.95	5	1.54E-02	1.99E-02	[IL10, NFKB1, PLCG1, RAF1, TNF]
	Gastric acid secretion	6.67	5	4.50E-03	7.86E-03	[ATP1B1, GNAS, KCNE2, KCNJ2, KCNQ1]
	Acute myeloid leukemia	7.58	5	2.59E-03	5.14E-03	[DUSP6, NFKB1, RAF1, STAT3, TCF7L2]
	TNF signaling pathway	9.26	10	3.43E-06	2.34E-05	[BCL3, CASP3, CCL2, IL1B, IL6, MMP3, MMP9, NFKB1, NOD2, TNF]
	Amoebiasis	9.38	9	9.68E-06	5.24E-05	[CASP3, COL1A1, GNAS, IL10, IL1B, IL6, NFKB1, TGFB1, TNF]
	cAMP signaling pathway	6.57	13	5.65E-06	3.41E-05	[ADRB1, ADRB2, ATP1B1, ATP2A2, ATP2B1, CACNA1C, GNAS, HCN4, NFKB1, PDE4D, RAF1, RYR2, TNNI3]
	Pancreatic secretion	6.25	6	2.62E-03	5.14E-03	[ATP1B1, ATP2A2, ATP2B1, GNAS, KCNQ1, RYR2]
	Non-small cell lung cancer	6.06	4	1.51E-02	1.97E-02	[PLCG1, POLK, RAF1, STAT3]
	Adrenaline,noradrenaline inhibits insulin secretion	14.29	4	6.53E-04	1.60E-03	[ADRA2C, CACNA1C, CACNB2, GNB3]
	Signaling by FGFR1 in disease	7.89	3	1.72E-02	2.19E-02	[PLCG1, STAT1, STAT3]
	Regulation of lipid metabolism by Peroxisome proliferator-activated receptor alpha (PPARalpha)	5.04	6	7.49E-03	1.20E-02	[ABCA1, ABCB4, AGT, FADS1, GRHL1, HMGCR]
	Voltage gated Potassium channels	11.63	5	3.64E-04	9.68E-04	[KCNA5, KCNAB2, KCND3, KCNH2, KCNQ1]
	Incretin synthesis, secretion, and inactivation	13.04	3	4.22E-03	7.45E-03	[GATA4, GNB3, TCF7L2]
	Regulation of TLR by endogenous ligand	15.79	3	2.41E-03	4.85E-03	[APOB, FGB, LY96]

Reactome	Signaling by SCF-KIT	9.30	4	3.33E-03	6.14E-03	[CMA1, MMP9, STAT1, STAT3]
	Interleukin-6 family signaling	16.67	4	3.54E-04	9.59E-04	[IL6, IL6R, STAT1, STAT3]
	Platelet homeostasis	6.67	6	1.89E-03	4.01E-03	[APOB, ATP2A2, ATP2B1, GNAS, GNB3, NOS3]
	Collagen degradation	6.25	4	1.36E-02	1.85E-02	[COL1A1, MMP2, MMP3, MMP9]
	G alpha (z) signalling events	6.38	3	3.01E-02	3.52E-02	[ADRA2C, GNAS, GNB3]
	Degradation of the extracellular matrix	5.76	8	9.02E-04	2.08E-03	[CASP3, CMA1, COL1A1, FBN1, MMP2, MMP3, MMP9, TIMP1]
	Regulation of insulin secretion	6.25	5	5.92E-03	9.89E-03	[ADRA2C, CACNA1C, CACNB2, GNAS, GNB3]
	Activation of Matrix Metalloproteinases	15.15	5	1.01E-04	3.44E-04	[CMA1, MMP2, MMP3, MMP9, TIMP1]
	Interleukin-10 signaling	18.37	9	2.74E-08	3.07E-07	[CCL2, IL10, IL18, IL1B, IL1RN, IL6, STAT3, TIMP1, TNF]
	Integration of energy metabolism	5.45	6	5.13E-03	8.76E-03	[ADRA2C, CACNA1C, CACNB2, GNAS, GNB3, STK11]
	Metal ion SLC transporters	11.54	3	6.01E-03	9.93E-03	[CP, SLC30A3, SLC39A8]
	Interaction between L1 and Ankyrins	22.58	7	2.25E-07	1.97E-06	[SCN10A, SCN1A, SCN1B, SCN3A, SCN3B, SCN4B, SCN5A]
	Plasma lipoprotein assembly, remodeling, and clearance	8.70	6	4.66E-04	1.18E-03	[ABCA1, APOB, APOC1, CETP, LIPC, LPL]
	Interleukin-1 family signaling	4.29	6	1.58E-02	2.04E-02	[IL18, IL1B, IL1RN, NFKB1, NOD2, STAT3]
	Downstream signal transduction	10.34	3	8.18E-03	1.26E-02	[PLCG1, STAT1, STAT3]
	Interleukin-4 and 13 signaling	13.89	15	3.58E-11	1.12E-09	[CCL2, HMOX1, IL10, IL18, IL1B, IL6, IL6R, MMP2, MMP3, MMP9, STAT1, STAT3, TGFB1, TIMP1, TNF]
	PPARA activates gene expression	5.13	6	6.91E-03	1.13E-02	[ABCA1, ABCB4, AGT, FADS1, GRHL1, HMGCR]
	Interleukin-12 family signaling	8.47	5	1.57E-03	3.43E-03	[IL10, IL27, MIF, STAT1, STAT3]
	Metabolism of Angiotensinogen to Angiotensins	35.29	6	8.95E-08	9.36E-07	[ACE, ACE2, AGT, C9orf3, CMA1, ENPEP]
	Interleukin-1 processing	42.86	3	9.89E-05	3.45E-04	[IL18, IL1B, NFKB1]
	Cell surface interactions at the vascular wall	5.07	7	3.83E-03	6.91E-03	[APOB, ATP1B1, CAV1, COL1A1, MIF, PLCG1, TGFB1]
	Metabolism of fat-soluble vitamins	6.38	3	3.01E-02	3.52E-02	[APOB, LPL, VKORC1]
	YAP1- and WWTR1 (TAZ)-stimulated gene expression	33.33	5	1.57E-06	1.12E-05	[GATA4, NKX2-5, NPPA, TBX5, TEAD4]
	Platelet Aggregation (Plug Formation)	7.69	3	1.84E-02	2.31E-02	[ADRA2C, COL1A1, FGB]
	Signaling by Interleukins	4.96	23	2.14E-07	1.98E-06	[CASP3, CCL2, DUSP6, HMOX1, IL10, IL18, IL1B, IL1RN, IL27, IL2RA, IL6, IL6R, MIF, MMP2, MMP3, MMP9, NFKB1, NOD2, STAT1, STAT3, TGFB1, TIMP1, TNF]
	Transcriptional regulation by RUNX3	5.15	5	1.31E-02	1.80E-02	[MAML3, TCF7L2, TEAD4, TGFB1, ZFHX3]
	Peptide hormone metabolism	10.11	9	5.17E-06	3.25E-05	[ACE, ACE2, AGT, C9orf3, CMA1, ENPEP, GATA4, GNB3, TCF7L2]
	Post-translational protein phosphorylation	4.63	5	2.00E-02	2.45E-02	[APOB, CP, FBN1, IL6, TIMP1]
	Plasma lipoprotein assembly	16.67	3	2.05E-03	4.24E-03	[ABCA1, APOB, APOC1]
	L1CAM interactions	5.88	7	1.66E-03	3.57E-03	[SCN10A, SCN1A, SCN1B, SCN3A, SCN3B, SCN4B, SCN5A]
	Plasma lipoprotein remodeling	13.33	4	8.54E-04	2.00E-03	[APOB, CETP, LIPC, LPL]
	Amine ligand-binding receptors	9.52	4	3.05E-03	5.77E-03	[ADRA2C, ADRB1, ADRB2, ADRB3]
	Plasma lipoprotein clearance	9.09	3	1.17E-02	1.70E-02	[APOB, APOC1, LIPC]
	Interleukin-2 family signaling	6.82	3	2.53E-02	3.02E-02	[IL2RA, STAT1, STAT3]
	PERK regulates gene expression	10.71	3	7.41E-03	1.20E-02	[CCL2, HERPUD1, HSPA5]
	Unfolded Protein Response (UPR)	5.10	5	1.36E-02	1.84E-02	[CCL2, DNAJB9, HERPUD1, HSPA5, LMNA]
	Signaling by NOTCH3	6.12	3	3.35E-02	3.87E-02	[MAML3, NEURL1, STAT1]
	Interleukin-6 signaling	36.36	4	1.27E-05	6.67E-05	[IL6, IL6R, STAT1, STAT3]
	Interleukin-27 signaling	27.27	3	4.47E-04	1.15E-03	[IL27, STAT1, STAT3]
	Phase 3 - rapid repolarisation	87.50	7	9.07E-13	3.56E-11	[KCNE1, KCNE2, KCNE3, KCNE4, KCNE5, KCNH2, KCNQ1]
	Transcriptional regulation of white adipocyte differentiation	4.55	4	3.83E-02	4.29E-02	[LPL, NFKB1, TGFB1, TNF]
	Cardiac conduction	21.99	31	1.96E-28	3.08E-26	[ATP1B1, ATP2A2, ATP2B1, CACNA1C, CACNA2D4, CACNB2, CASQ2, CORIN, GATA4, KCND3, KCNE1, KCNE2, KCNE3, KCNE4, KCNE5, KCNH2, KCNJ2, KCNQ1, NKX2-5, NPPA, RYR2, SCN10A, SCN1A, SCN1B, SCN3A, SCN3B, SCN4B, SCN5A, SLN, TBX5, TNNI3]
	Neuronal System	4.34	16	8.57E-05	3.06E-04	[CACNB2, GNB3, HCN4, KCNA5, KCNAB2, KCND3, KCNH2, KCNJ2, KCNJ3, KCNJ5, KCNJ8, KCNN2, KCNN3, KCNQ1, PPFIA4, RAF1]
	Ion transport by P-type ATPases	7.02	4	9.11E-03	1.38E-02	[ATP1B1, ATP2A2, ATP2B1, SLN]
	Regulation of Insulin-like Growth Factor (IGF) transport and uptake by Insulin-like Growth Factor Binding Proteins (IGFBPs)	4.80	6	9.43E-03	1.41E-02	[APOB, CP, FBN1, IL6, MMP2, TIMP1]
	GABA receptor activation	7.27	4	8.05E-03	1.25E-02	[GNB3, KCNJ2, KCNJ3, KCNJ5]
	Nuclear Receptor transcription pathway	6.00	3	3.53E-02	4.04E-02	[ESR1, ESR2, NRBP1]
	Striated Muscle Contraction	8.33	3	1.49E-02	1.96E-02	[MYL4, TNNI3, TNNT2]
	RAF-independent MAPK1/3 activation	13.04	3	4.22E-03	7.45E-03	[DUSP6, IL6, IL6R]
	Adrenoceptors	44.44	4	4.98E-06	3.26E-05	[ADRA2C, ADRB1, ADRB2, ADRB3]
	GABA B receptor activation	10.26	4	2.32E-03	4.73E-03	[GNB3, KCNJ2, KCNJ3, KCNJ5]
	Activation of G protein gated Potassium channels	16.00	4	4.17E-04	1.09E-03	[GNB3, KCNJ2, KCNJ3, KCNJ5]
	Phase 0 - rapid depolarisation	21.74	10	7.79E-10	1.22E-08	[CACNA1C, CACNA2D4, CACNB2, SCN10A, SCN1A, SCN1B, SCN3A, SCN3B, SCN4B, SCN5A]
	Muscle contraction	16.02	33	1.61E-25	1.26E-23	[ATP1B1, ATP2A2, ATP2B1, CACNA1C, CACNA2D4, CACNB2, CASQ2, CORIN, GATA4, KCND3, KCNE1, KCNE2, KCNE3, KCNE4, KCNE5, KCNH2, KCNJ2, KCNQ1, MYL4, NKX2-5, NPPA, RYR2, SCN10A, SCN1A, SCN1B, SCN3A, SCN3B, SCN4B, SCN5A, SLN, TBX5, TNNI3, TNNT2]
	G protein gated Potassium channels	16.00	4	4.17E-04	1.09E-03	[GNB3, KCNJ2, KCNJ3, KCNJ5]
	Activation of GABAB receptors	10.26	4	2.32E-03	4.73E-03	[GNB3, KCNJ2, KCNJ3, KCNJ5]
	Phase 2 - plateau phase	33.33	9	8.20E-11	2.14E-09	[CACNA1C, CACNA2D4, CACNB2, KCNE1, KCNE2, KCNE3, KCNE4, KCNE5, KCNQ1]
	Inwardly rectifying K+ channels	16.13	5	7.38E-05	2.76E-04	[GNB3, KCNJ2, KCNJ3, KCNJ5, KCNJ8]
	Physiological factors	41.67	5	4.30E-07	3.37E-06	[CORIN, GATA4, NKX2-5, NPPA, TBX5]
	Inhibition of voltage gated Ca2+ channels via Gbeta/gamma subunits	16.00	4	4.17E-04	1.09E-03	[GNB3, KCNJ2, KCNJ3, KCNJ5]
	Potassium Channels	13.13	13	1.56E-09	2.22E-08	[GNB3, HCN4, KCNA5, KCNAB2, KCND3, KCNH2, KCNJ2, KCNJ3, KCNJ5, KCNJ8, KCNN2, KCNN3, KCNQ1]
	Ion homeostasis	12.50	7	1.47E-05	7.21E-05	[ATP1B1, ATP2A2, ATP2B1, CASQ2, RYR2, SLN, TNNI3]

Supplement Table 3 Assessment of AF-specific subnetwork constructed by the prize-collecting Steiner forest algorithm

	Meta-analysis	Network Analysis													
name	p value	Prize node	Hiddennode	FractionOfOptimal ForestsContaining (noisyEdges)	FractionOfOptimal ForestsContaining (randomTerminals)	Degree	Average Shortest Path Length	Betweenness Centrality	ClosenessCentrality	Eccentricity	Neighborhood Connectivity	Number Of DirectedEdges	Radiality	Stress	Topological Coefficient
**GJA5**	NA	√	×	1	0	1	3.87	0.00	0.26	6	2.00	1	0.64	0	0.00
ABCA1	NA	√	×	1	0.1	1	2.88	0.00	0.35	5	115.00	1	0.76	0	0.00
**ABCA12**	NA	√	×	1	0	1	2.88	0.00	0.35	5	115.00	1	0.76	0	0.00
**ABCB4**	NA	√	×	1	0	1	3.87	0.00	0.26	6	2.00	1	0.64	0	0.00
**ACE**	0.008742	√	×	1	0	1	5.31	0.00	0.19	8	5.00	1	0.46	0	0.00
**AGT**	NA	√	×	1	0	5	4.31	0.03	0.23	7	1.40	5	0.59	2148	0.20
ACE2	NA	√	×	1	0.1	1	5.31	0.00	0.19	8	5.00	1	0.46	0	0.00
**SCN10A**	NA	√	×	1	0	1	3.87	0.00	0.26	6	2.00	1	0.64	0	0.00
**KCNA5**	NA	√	×	1	0	1	3.86	0.00	0.26	6	3.00	1	0.64	0	0.00
**MYOZ1**	NA	√	×	1	0	1	3.86	0.00	0.26	6	3.00	1	0.64	0	0.00
**ADH1B**	NA	√	×	1	0	1	3.86	0.00	0.26	6	3.00	1	0.64	0	0.00
**ADRA2C**	NA	√	×	1	0	1	3.87	0.00	0.26	6	2.00	1	0.64	0	0.00
**ADRB1**	NA	√	×	1	0	1	3.87	0.00	0.26	6	2.00	1	0.64	0	0.00
**ADRB2**	NA	√	×	1	0	2	2.87	0.01	0.35	5	58.00	2	0.77	540	0.50
**KCNJ5**	NA	√	×	1	0	1	3.87	0.00	0.26	6	2.00	1	0.64	0	0.00
ADRB3	NA	√	×	1	0.1	1	4.39	0.00	0.23	7	2.00	1	0.58	0	0.00
**AGTR1**	NA	√	×	1	0	1	5.31	0.00	0.19	8	5.00	1	0.46	0	0.00
**ENPEP**	NA	√	×	1	0	1	5.31	0.00	0.19	8	5.00	1	0.46	0	0.00
AGXT2	NA	√	×	1	0.1	1	5.29	0.00	0.19	8	5.00	1	0.46	0	0.00
**SCN1B**	NA	√	×	1	0	1	4.76	0.00	0.21	7	3.00	1	0.53	0	0.00
**SCN5A**	NA	√	×	1	0	5	2.79	0.09	0.36	5	25.00	5	0.78	6826	0.20
**SLC30A3**	NA	√	×	1	0	1	3.87	0.00	0.26	6	2.00	1	0.64	0	0.00
**CNTN5**	NA	√	×	1	0	1	5.37	0.00	0.19	8	2.00	1	0.45	0	0.00
**APOB**	NA	√	×	1	0	1	2.88	0.00	0.35	5	115.00	1	0.76	0	0.00
**APOC1**	NA	√	×	1	0	1	5.38	0.00	0.19	8	2.00	1	0.45	0	0.00
**VKORC1**	NA	√	×	1	0	2	4.38	0.01	0.23	7	1.50	2	0.58	540	0.50
**FSD1L**	NA	√	×	1	0	1	3.84	0.00	0.26	6	6.00	1	0.64	0	0.00
**KCNE1L**	NA	√	×	1	0	1	3.84	0.00	0.26	6	6.00	1	0.64	0	0.00
**MX1**	NA	√	×	1	0	1	3.84	0.00	0.26	6	6.00	1	0.64	0	0.00
PSRC1	NA	√	×	1	0.1	1	3.84	0.00	0.26	6	6.00	1	0.64	0	0.00
**TNNT2**	NA	√	×	1	0	1	3.84	0.00	0.26	6	6.00	1	0.64	0	0.00
**ARL5B**	NA	√	×	1	0	1	2.88	0.00	0.35	5	115.00	1	0.76	0	0.00
**CETP**	NA	√	×	1	0	1	3.87	0.00	0.26	6	2.00	1	0.64	0	0.00
**ATP1B1**	NA	√	×	1	0	1	2.88	0.00	0.35	5	115.00	1	0.76	0	0.00
**ATP2A2**	NA	√	×	1	0	1	2.88	0.00	0.35	5	115.00	1	0.76	0	0.00
**ATP2B1**	NA	√	×	1	0	1	2.88	0.00	0.35	5	115.00	1	0.76	0	0.00
BAZ1B	NA	√	×	1	0.1	1	2.88	0.00	0.35	5	115.00	1	0.76	0	0.00
**BCL3**	NA	√	×	1	0	1	5.31	0.00	0.19	8	3.00	1	0.46	0	0.00
NFKB1	NA	√	×	1	0.2	3	4.31	0.01	0.23	7	2.67	3	0.59	1078	0.33
**C9orf3**	NA	√	×	1	0	1	5.29	0.00	0.19	8	5.00	1	0.46	0	0.00
**CACNA1C**	NA	√	×	1	0	1	4.75	0.00	0.21	7	4.00	1	0.53	0	0.00
**CACNB2**	0.004139	√	×	1	0	1	3.87	0.00	0.26	6	2.00	1	0.64	0	0.00
**KCNN2**	0.00025937	√	×	1	0	1	4.75	0.00	0.21	7	4.00	1	0.53	0	0.00
**CAND2**	NA	√	×	1	0	1	2.88	0.00	0.35	5	115.00	1	0.76	0	0.00
CASP3	NA	√	×	1	0.1	1	3.87	0.00	0.26	6	2.00	1	0.64	0	0.00
**CASQ2**	NA	√	×	1	0	1	5.74	0.00	0.17	8	2.00	1	0.41	0	0.00
CAV1	NA	√	×	1	0.1	2	2.87	0.01	0.35	5	58.00	2	0.77	540	0.50
**CAV2**	NA	√	×	1	0	1	3.87	0.00	0.26	6	2.00	1	0.64	0	0.00
**CCL2**	NA	√	×	1	0	1	3.86	0.00	0.26	6	3.00	1	0.64	0	0.00
**CD200**	NA	√	×	1	0	1	3.87	0.00	0.26	6	2.00	1	0.64	0	0.00
SHOX2	NA	√	×	1	0.1	1	3.87	0.00	0.26	6	2.00	1	0.64	0	0.00
ESR1	0.02808	√	×	1	0.7	18	2.41	0.41	0.42	5	8.61	18	0.82	30160	0.06
IL1B	NA	√	×	1	0.1	1	4.39	0.00	0.23	7	2.00	1	0.58	0	0.00
**CELSR2**	0.00040243	√	×	1	0	1	2.88	0.00	0.35	5	115.00	1	0.76	0	0.00
**CMA1**	NA	√	×	1	0	1	5.29	0.00	0.19	8	5.00	1	0.46	0	0.00
**COL1A1**	0.0082643	√	×	1	0	1	2.88	0.00	0.35	5	115.00	1	0.76	0	0.00
**COL4A3BP**	NA	√	×	1	0	1	2.88	0.00	0.35	5	115.00	1	0.76	0	0.00
**COX8A**	0.047638	√	×	1	0	1	2.88	0.00	0.35	5	115.00	1	0.76	0	0.00
**CP**	NA	√	×	1	0	1	2.88	0.00	0.35	5	115.00	1	0.76	0	0.00
**MEIS1**	NA	√	×	1	0	1	5.38	0.00	0.19	8	2.00	1	0.45	0	0.00
**CRP**	NA	√	×	1	0	1	5.31	0.00	0.19	8	3.00	1	0.46	0	0.00
**TCF7L2**	0.0084114	√	×	1	0	1	3.87	0.00	0.26	6	2.00	1	0.64	0	0.00
**POC1B**	NA	√	×	1	0	1	3.87	0.00	0.26	6	2.00	1	0.64	0	0.00
**CUX2**	NA	√	×	1	0	1	4.33	0.00	0.23	7	6.00	1	0.58	0	0.00
**CYP11B2**	NA	√	×	1	0	1	5.38	0.00	0.19	8	2.00	1	0.45	0	0.00
SUGP1	NA	√	×	1	0.1	1	3.87	0.00	0.26	6	2.00	1	0.64	0	0.00
**DMRTA1**	NA	√	×	1	0	1	3.86	0.00	0.26	6	3.00	1	0.64	0	0.00
**DNAH2**	NA	√	×	1	0	1	3.41	0.00	0.29	6	18.00	1	0.70	0	0.00
DNAJB9	NA	√	×	1	0.1	1	3.87	0.00	0.26	6	2.00	1	0.64	0	0.00
DUSP6	0.036567	√	×	1	0.1	1	3.86	0.00	0.26	6	3.00	1	0.64	0	0.00
**JPH2**	NA	√	×	1	0	1	5.32	0.00	0.19	8	2.00	1	0.46	0	0.00
**EDN2**	NA	√	×	1	0	1	5.38	0.00	0.19	8	2.00	1	0.45	0	0.00
KCNE3	NA	√	×	1	0.2	1	3.87	0.00	0.26	6	2.00	1	0.64	0	0.00
PLCG1	NA	√	×	1	0.2	1	3.87	0.00	0.26	6	2.00	1	0.64	0	0.00
**SLN**	0.025159	√	×	1	0	1	5.29	0.00	0.19	8	5.00	1	0.46	0	0.00
**GATA5**	NA	√	×	1	0	1	4.32	0.00	0.23	7	5.00	1	0.58	0	0.00
**IL6**	NA	√	×	1	0	2	4.31	0.01	0.23	7	3.00	2	0.59	540	0.50
**EPHX2**	NA	√	×	1	0	1	2.88	0.00	0.35	5	115.00	1	0.76	0	0.00
**ESR2**	NA	√	×	1	0	1	3.41	0.00	0.29	6	18.00	1	0.70	0	0.00
**NPPA**	0.017654	√	×	1	0	2	3.39	0.01	0.29	6	10.00	2	0.70	1076	0.50
**FADS1**	NA	√	×	1	0	1	2.88	0.00	0.35	5	115.00	1	0.76	0	0.00
**FANCC**	NA	√	×	1	0	1	3.87	0.00	0.26	6	2.00	1	0.64	0	0.00
**FBN1**	0.0044747	√	×	1	0	1	3.87	0.00	0.26	6	2.00	1	0.64	0	0.00
LMNA	NA	√	×	1	0.2	2	2.87	0.01	0.35	5	58.00	2	0.77	540	0.50
**FERD3L**	NA	√	×	1	0	1	5.30	0.00	0.19	8	3.00	1	0.46	0	0.00
**FGB**	NA	√	×	1	0	1	3.86	0.00	0.26	6	3.00	1	0.64	0	0.00
**KCNE4**	NA	√	×	1	0	1	3.86	0.00	0.26	6	3.00	1	0.64	0	0.00
**KCNJ2**	0.02387	√	×	1	0	1	3.86	0.00	0.26	6	3.00	1	0.64	0	0.00
**FTO**	NA	√	×	1	0	1	2.88	0.00	0.35	5	115.00	1	0.76	0	0.00
**HCN4**	0.013774	√	×	1	0	1	3.87	0.00	0.26	6	2.00	1	0.64	0	0.00
GATA4	NA	√	×	1	0.1	1	4.39	0.00	0.23	7	3.00	1	0.58	0	0.00
GATA6	NA	√	×	1	0.1	1	4.39	0.00	0.23	7	3.00	1	0.58	0	0.00
**GCKR**	NA	√	×	1	0	1	3.86	0.00	0.26	6	3.00	1	0.64	0	0.00
GJA1	NA	√	×	1	0.1	1	2.88	0.00	0.35	5	115.00	1	0.76	0	0.00
**KCNJ3**	0.00058957	√	×	1	0	1	3.87	0.00	0.26	6	2.00	1	0.64	0	0.00
PDE4D	NA	√	×	1	0.1	1	3.86	0.00	0.26	6	3.00	1	0.64	0	0.00
**GNB3**	NA	√	×	1	0	1	3.87	0.00	0.26	6	2.00	1	0.64	0	0.00
**GORAB**	NA	√	×	1	0	1	5.32	0.00	0.19	8	3.00	1	0.46	0	0.00
**GREM2**	NA	√	×	1	0	1	3.87	0.00	0.26	6	2.00	1	0.64	0	0.00
**GRHL1**	NA	√	×	1	0	1	5.75	0.00	0.17	8	2.00	1	0.41	0	0.00
GRK5	NA	√	×	1	0.1	1	3.87	0.00	0.26	6	2.00	1	0.64	0	0.00
**PRRX1**	NA	√	×	1	0	1	3.87	0.00	0.26	6	2.00	1	0.64	0	0.00
HAND2	NA	√	×	1	0.1	1	2.88	0.00	0.35	5	115.00	1	0.76	0	0.00
**NKX2-5**	NA	√	×	1	0	1	4.33	0.00	0.23	7	6.00	1	0.58	0	0.00
**PRDM1**	NA	√	×	1	0	1	5.31	0.00	0.19	8	3.00	1	0.46	0	0.00
**TBX3**	NA	√	×	1	0	1	5.31	0.00	0.19	8	3.00	1	0.46	0	0.00
**HERPUD1**	NA	√	×	1	0	1	2.88	0.00	0.35	5	115.00	1	0.76	0	0.00
**HMCN1**	NA	√	×	1	0	1	2.88	0.00	0.35	5	115.00	1	0.76	0	0.00
**HMGCR**	NA	√	×	1	0	1	2.88	0.00	0.35	5	115.00	1	0.76	0	0.00
**HMOX1**	0.018655	√	×	1	0	1	2.88	0.00	0.35	5	115.00	1	0.76	0	0.00
**PCDH7**	NA	√	×	1	0	1	3.87	0.00	0.26	6	2.00	1	0.64	0	0.00
**NOS3**	NA	√	×	1	0	1	3.86	0.00	0.26	6	3.00	1	0.64	0	0.00
**HSPA5**	0.0080822	√	×	1	0	2	2.87	0.01	0.35	5	58.00	2	0.77	540	0.50
**KCND3**	0.0054	√	×	1	0	1	3.87	0.00	0.26	6	2.00	1	0.64	0	0.00
**IL10**	NA	√	×	1	0	1	3.87	0.00	0.26	6	2.00	1	0.64	0	0.00
IL18	NA	√	×	1	0.1	1	2.88	0.00	0.35	5	115.00	1	0.76	0	0.00
**IL1RN**	NA	√	×	1	0	1	3.87	0.00	0.26	6	2.00	1	0.64	0	0.00
IL2RA	NA	√	×	1	0.1	1	5.38	0.00	0.19	8	2.00	1	0.45	0	0.00
**IL6R**	NA	√	×	1	0	1	5.31	0.00	0.19	8	2.00	1	0.46	0	0.00
**ITPK1**	NA	√	×	1	0	1	3.86	0.00	0.26	6	3.00	1	0.64	0	0.00
**TBX5**	NA	√	×	1	0	1	4.38	0.00	0.23	7	3.00	1	0.58	0	0.00
KCNAB2	NA	√	×	1	0.1	1	2.88	0.00	0.35	5	115.00	1	0.76	0	0.00
**KCNE1**	NA	√	×	1	0	1	3.86	0.00	0.26	6	3.00	1	0.64	0	0.00
**KCNQ1**	NA	√	×	1	0	3	2.87	0.01	0.35	5	39.00	3	0.77	1078	0.33
**KCNE2**	NA	√	×	1	0	1	3.86	0.00	0.26	6	3.00	1	0.64	0	0.00
**KCNH2**	NA	√	×	1	0	1	2.88	0.00	0.35	5	115.00	1	0.76	0	0.00
KCNJ8	NA	√	×	1	0.1	1	3.87	0.00	0.26	6	2.00	1	0.64	0	0.00
**KCNN3**	NA	√	×	1	0	1	2.88	0.00	0.35	5	115.00	1	0.76	0	0.00
**MYH7**	NA	√	×	1	0	1	3.87	0.00	0.26	6	2.00	1	0.64	0	0.00
**LIPC**	NA	√	×	1	0	1	3.86	0.00	0.26	6	4.00	1	0.64	0	0.00
**LPL**	NA	√	×	1	0	1	3.86	0.00	0.26	6	4.00	1	0.64	0	0.00
**SCN3A**	NA	√	×	1	0	1	3.86	0.00	0.26	6	4.00	1	0.64	0	0.00
**LY96**	NA	√	×	1	0	1	3.87	0.00	0.26	6	2.00	1	0.64	0	0.00
**MAML3**	NA	√	×	1	0	1	3.87	0.00	0.26	6	2.00	1	0.64	0	0.00

RAF1	NA	√	×	1	0.1	1	4.85	0.00	0.21	7	2.00	1	0.52	0	0.00
**MIF**	9.8484E-06	√	×	1	0	1	2.88	0.00	0.35	5	115.00	1	0.76	0	0.00
**MMP2**	NA	√	×	1	0	1	3.86	0.00	0.26	6	4.00	1	0.64	0	0.00
TIMP1	0.030687	√	×	1	0.1	4	2.86	0.02	0.35	5	29.50	4	0.77	1614	0.25
**MMP3**	NA	√	×	1	0	1	3.86	0.00	0.26	6	4.00	1	0.64	0	0.00
MMP9	0.01915	√	×	1	0.1	1	3.86	0.00	0.26	6	4.00	1	0.64	0	0.00
**MTHFR**	NA	√	×	1	0	1	3.87	0.00	0.26	6	2.00	1	0.64	0	0.00
**MYL4**	0.034349	√	×	1	0	1	2.88	0.00	0.35	5	115.00	1	0.76	0	0.00
**PPFIA4**	NA	√	×	1	0	1	4.39	0.00	0.23	7	2.00	1	0.58	0	0.00
**NEBL**	NA	√	×	1	0	1	3.87	0.00	0.26	6	2.00	1	0.64	0	0.00
**SCN1A**	NA	√	×	1	0	1	3.87	0.00	0.26	6	2.00	1	0.64	0	0.00
**SCN3B**	NA	√	×	1	0	1	3.87	0.00	0.26	6	2.00	1	0.64	0	0.00
**NOD2**	NA	√	×	1	0	1	3.87	0.00	0.26	6	2.00	1	0.64	0	0.00
**NRBP1**	NA	√	×	1	0	1	2.88	0.00	0.35	5	115.00	1	0.76	0	0.00
**POLK**	NA	√	×	1	0	1	3.86	0.00	0.26	6	3.00	1	0.64	0	0.00
**SEMA3E**	NA	√	×	1	0	1	3.87	0.00	0.26	6	2.00	1	0.64	0	0.00
**POC5**	NA	√	×	1	0	1	2.88	0.00	0.35	5	115.00	1	0.76	0	0.00
**TWISTNB**	NA	√	×	1	0	1	3.87	0.00	0.26	6	2.00	1	0.64	0	0.00
**SYNPO2L**	NA	√	×	1	0	1	3.87	0.00	0.26	6	2.00	1	0.64	0	0.00
**RAD51B**	NA	√	×	1	0	1	3.87	0.00	0.26	6	2.00	1	0.64	0	0.00
RBFOX1	0.0010792	√	×	1	0.1	1	2.88	0.00	0.35	5	115.00	1	0.76	0	0.00
**WDPCP**	NA	√	×	1	0	1	5.32	0.00	0.19	8	3.00	1	0.46	0	0.00
TNF	NA	√	×	1	0.1	1	3.87	0.00	0.26	6	2.00	1	0.64	0	0.00
**RPLP1**	NA	√	×	1	0	1	2.88	0.00	0.35	5	115.00	1	0.76	0	0.00
**RYR2**	0.0059292	√	×	1	0	1	2.88	0.00	0.35	5	115.00	1	0.76	0	0.00
SCN4B	NA	√	×	0.9	0	1	5.75	0.00	0.17	8	2.00	1	0.41	0	0.00
**SEMA5B**	NA	√	×	1	0	1	3.86	0.00	0.26	6	3.00	1	0.64	0	0.00
**SH3PXD2A**	0.044418	√	×	1	0	1	2.88	0.00	0.35	5	115.00	1	0.76	0	0.00
**SIK3**	NA	√	×	1	0	1	2.88	0.00	0.35	5	115.00	1	0.76	0	0.00
**SOX5**	NA	√	×	1	0	1	3.87	0.00	0.26	6	2.00	1	0.64	0	0.00
**SPSB4**	NA	√	×	1	0	1	3.87	0.00	0.26	6	2.00	1	0.64	0	0.00
**STAT1**	NA	√	×	1	0	1	2.88	0.00	0.35	5	115.00	1	0.76	0	0.00
STAT3	NA	√	×	1	0.1	1	2.88	0.00	0.35	5	115.00	1	0.76	0	0.00
STK38	0.017012	√	×	1	0.1	1	2.88	0.00	0.35	5	115.00	1	0.76	0	0.00
**SULF2**	NA	√	×	1	0	1	2.88	0.00	0.35	5	115.00	1	0.76	0	0.00
**SYNE2**	0.0014391	√	×	1	0	1	2.88	0.00	0.35	5	115.00	1	0.76	0	0.00
TCF12	NA	√	×	1	0.1	1	5.30	0.00	0.19	8	3.00	1	0.46	0	0.00
**TEAD4**	NA	√	×	1	0	1	2.88	0.00	0.35	5	115.00	1	0.76	0	0.00
TGFB1	0.00016919	√	×	1	0.1	1	3.87	0.00	0.26	6	2.00	1	0.64	0	0.00
**TLE4**	NA	√	×	1	0	1	2.88	0.00	0.35	5	115.00	1	0.76	0	0.00
**TNNI3**	0.0016815	√	×	1	0	1	3.87	0.00	0.26	6	2.00	1	0.64	0	0.00
**TRIB1**	NA	√	×	1	0	1	2.88	0.00	0.35	5	115.00	1	0.76	0	0.00
**TSEN15**	NA	√	×	1	0	1	2.88	0.00	0.35	5	115.00	1	0.76	0	0.00
USO1	NA	√	×	1	0.1	1	2.88	0.00	0.35	5	115.00	1	0.76	0	0.00
**VOPP1**	0.014964	√	×	1	0	1	2.88	0.00	0.35	5	115.00	1	0.76	0	0.00
**ZC3HC1**	NA	√	×	1	0	1	2.88	0.00	0.35	5	115.00	1	0.76	0	0.00
ZCCHC8	NA	√	×	1	0.1	1	2.88	0.00	0.35	5	115.00	1	0.76	0	0.00
**ZFHX3**	NA	√	×	1	0	1	2.88	0.00	0.35	5	115.00	1	0.76	0	0.00
AAR2	NA	×	√	0.7	0	2	2.87	0.01	0.35	5	58.00	2	0.77	540	0.50
UBC	NA	×	√	1	1	115	1.89	0.94	0.53	4	1.86	115	0.89	68616	0.01
HAX1	NA	×	√	0.8	0	2	2.87	0.01	0.35	5	58.00	2	0.77	540	0.50
ACTB	NA	×	√	1	0.1	2	2.87	0.01	0.35	5	58.00	2	0.77	540	0.50
ACTN2	NA	×	√	1	0.1	3	2.87	0.01	0.35	5	39.00	3	0.77	1078	0.33
GNB2L1	NA	×	√	1	0.3	3	2.87	0.01	0.35	5	39.00	3	0.77	1078	0.33
UCHL1	NA	×	√	1	0.1	2	2.87	0.01	0.35	5	58.00	2	0.77	540	0.50
**GIPC1**	NA	×	√	1	0	2	2.87	0.01	0.35	5	58.00	2	0.77	540	0.50
SRC	NA	×	√	1	0.4	2	3.40	0.01	0.29	6	9.50	2	0.70	540	0.50
TP53	0.02385	×	√	1	0.6	3	3.35	0.06	0.30	6	8.67	3	0.71	4238	0.33
EGR1	NA	×	√	1	0.3	5	4.29	0.03	0.23	7	1.80	5	0.59	2148	0.20
ANK3	NA	×	√	0.8	0	3	3.76	0.02	0.27	6	2.67	3	0.65	1612	0.33
SCN2A	NA	×	√	0.9	0	2	4.75	0.01	0.21	7	2.00	2	0.53	540	0.50
AP3B1	NA	×	√	0.9	0	2	2.87	0.01	0.35	5	58.00	2	0.77	540	0.50
**APLP1**	0.017241	×	√	1	0	2	4.37	0.01	0.23	7	2.00	2	0.58	540	0.50
KAT5	NA	×	√	1	0.1	3	3.38	0.02	0.30	6	7.00	3	0.70	1612	0.33
APP	NA	×	√	1	1	6	2.85	0.04	0.35	5	20.00	6	0.77	2680	0.17
ARPC3	0.014423	×	√	0.9	0.1	2	2.87	0.01	0.35	5	58.00	2	0.77	540	0.50
CALM3	NA	×	√	1	0.4	4	3.75	0.03	0.27	6	2.25	4	0.66	2146	0.25
PRKACA	NA	×	√	1	0.2	2	2.87	0.01	0.35	5	58.00	2	0.77	540	0.50
TRDN	0.019353	×	√	0.8	0	2	4.74	0.01	0.21	7	2.50	2	0.53	540	0.50
XIAP	NA	×	√	1	0.2	2	2.87	0.01	0.35	5	58.00	2	0.77	540	0.50
PCNA	NA	×	√	1	0.4	3	2.87	0.01	0.35	5	39.00	3	0.77	1078	0.33
**HUS1**	NA	×	√	1	0	2	2.87	0.01	0.35	5	58.00	2	0.77	540	0.50
CDK4	0.017629	×	√	1	0.1	2	2.87	0.01	0.35	5	58.00	2	0.77	540	0.50
CEBPB	NA	×	√	1	0.2	2	3.40	0.01	0.29	6	9.50	2	0.70	540	0.50
CFTR	NA	×	√	1	0.5	1	2.88	0.00	0.35	5	115.00	1	0.76	0	0.00
**CREB1**	0.0018508	×	√	1	0	2	4.38	0.01	0.23	7	1.50	2	0.58	540	0.50
**CREBBP**	0.02387	×	√	1	0	2	3.39	0.01	0.29	6	10.00	2	0.70	1076	0.50
CTNNB1	0.016471	×	√	1	0.8	2	2.87	0.01	0.35	5	58.00	2	0.77	540	0.50
**CUL4A**	NA	×	√	1	0	2	2.87	0.01	0.35	5	58.00	2	0.77	540	0.50
HDAC1	NA	×	√	1	0.7	6	3.33	0.07	0.30	6	4.67	6	0.71	5296	0.17
CYP11A1	NA	×	√	0.6	0.1	2	4.38	0.01	0.23	7	1.50	2	0.58	540	0.50
SMAD2	NA	×	√	1	0.2	2	3.39	0.01	0.29	6	10.00	2	0.70	1076	0.50
DHX15	NA	×	√	1	0.2	2	2.87	0.01	0.35	5	58.00	2	0.77	540	0.50
HSP90AA1	NA	×	√	1	0.8	3	2.87	0.01	0.35	5	39.00	3	0.77	1078	0.33
PLK1	0.024312	×	√	1	0.2	2	2.87	0.01	0.35	5	58.00	2	0.77	540	0.50
MAPK1	NA	×	√	1	0.1	3	2.86	0.02	0.35	5	39.33	3	0.77	1612	0.33
**E2F4**	NA	×	√	1	0	2	4.32	0.01	0.23	7	3.50	2	0.58	540	0.50
MME	NA	×	√	0.9	0	2	4.38	0.01	0.23	7	1.50	2	0.58	540	0.50
EEF1A1	NA	×	√	1	0.2	2	2.87	0.01	0.35	5	58.00	2	0.77	540	0.50
EGFR	NA	×	√	1	0.7	2	2.87	0.01	0.35	5	58.00	2	0.77	540	0.50
EP300	NA	×	√	1	0.6	5	3.32	0.08	0.30	6	5.80	5	0.71	5802	0.20
**TCF3**	NA	×	√	1	0	3	4.31	0.01	0.23	7	2.33	3	0.59	1078	0.33
JUN	0.012215	×	√	1	0.7	2	3.39	0.01	0.29	6	10.00	2	0.70	1076	0.50
NCOA1	0.048905	×	√	1	0.2	1	3.41	0.00	0.29	6	18.00	1	0.70	0	0.00
NCOA2	NA	×	√	1	0.1	2	3.40	0.01	0.29	6	9.50	2	0.70	540	0.50
**NCOA3**	NA	×	√	1	0	1	3.41	0.00	0.29	6	18.00	1	0.70	0	0.00
SP1	NA	×	√	1	0.2	3	3.39	0.01	0.29	6	6.67	3	0.70	1078	0.33
TRAM1	NA	×	√	0.5	0	2	3.39	0.01	0.29	6	10.00	2	0.70	1076	0.50
FANCA	NA	×	√	1	0.1	2	2.87	0.01	0.35	5	58.00	2	0.77	540	0.50
**TK1**	NA	×	√	1	0	3	2.87	0.01	0.35	5	39.00	3	0.77	1078	0.33
FLNA	0.040517	×	√	1	0.1	3	2.87	0.01	0.35	5	39.00	3	0.77	1078	0.33
FYN	0.017629	×	√	1	0.2	2	2.87	0.01	0.35	5	58.00	2	0.77	540	0.50
TRAF2	NA	×	√	1	0.5	3	2.87	0.01	0.35	5	39.00	3	0.77	1078	0.33
GNB1	NA	×	√	1	0.2	2	2.87	0.01	0.35	5	58.00	2	0.77	540	0.50
GNG5	NA	×	√	0.8	0	2	2.87	0.01	0.35	5	58.00	2	0.77	540	0.50
**RCHY1**	NA	×	√	1	0	3	4.33	0.01	0.23	7	1.67	3	0.58	1078	0.33
**STK3**	NA	×	√	1	0	2	2.87	0.01	0.35	5	58.00	2	0.77	540	0.50
**GRHL3**	NA	×	√	1	0	2	4.76	0.01	0.21	7	1.50	2	0.53	540	0.50
**PRMT1**	0.0010142	×	√	1	0	2	3.77	0.01	0.27	6	3.50	2	0.65	1076	0.50
SNCA	NA	×	√	1	0.3	2	2.87	0.01	0.35	5	58.00	2	0.77	540	0.50
**GTF2I**	NA	×	√	1	0	2	2.87	0.01	0.35	5	58.00	2	0.77	540	0.50
HDAC2	NA	×	√	1	0.1	3	4.31	0.01	0.23	7	2.67	3	0.59	1078	0.33
**HNRNPU**	NA	×	√	1	0	2	2.87	0.01	0.35	5	58.00	2	0.77	540	0.50
**IRAK1**	0.016736	×	√	1	0	2	2.87	0.01	0.35	5	58.00	2	0.77	540	0.50
**IL1R1**	NA	×	√	1	0	2	2.87	0.01	0.35	5	58.00	2	0.77	540	0.50
IL2	NA	×	√	1	0.1	2	4.38	0.01	0.23	7	1.50	2	0.58	540	0.50
IL2RB	NA	×	√	0.8	0.2	1	2.88	0.00	0.35	5	115.00	1	0.76	0	0.00
KCNJ11	NA	×	√	0.9	0.1	2	2.87	0.01	0.35	5	58.00	2	0.77	540	0.50
KIAA0368	NA	×	√	0.9	0	2	2.87	0.01	0.35	5	58.00	2	0.77	540	0.50
LRP1	NA	×	√	0.9	0.1	4	2.86	0.02	0.35	5	29.50	4	0.77	1614	0.25
**TLR4**	NA	×	√	1	0	2	2.87	0.01	0.35	5	58.00	2	0.77	540	0.50
NOTCH1	NA	×	√	1	0.2	2	2.87	0.01	0.35	5	58.00	2	0.77	540	0.50
MAP2K1	0.049167	×	√	1	0.1	2	3.85	0.01	0.26	6	2.00	2	0.64	540	0.50
PSMA1	NA	×	√	1	0.1	2	2.87	0.01	0.35	5	58.00	2	0.77	540	0.50
ZYX	0.02877	×	√	0.9	0	2	2.87	0.01	0.35	5	58.00	2	0.77	540	0.50
**NEDD8**	NA	×	√	1	0	2	2.87	0.01	0.35	5	58.00	2	0.77	540	0.50
NFASC	NA	×	√	1	0.1	2	2.87	0.01	0.35	5	58.00	2	0.77	540	0.50
RIPK2	NA	×	√	0.9	0	2	2.87	0.01	0.35	5	58.00	2	0.77	540	0.50
**PLXND1**	0.034709	×	√	1	0	2	2.87	0.01	0.35	5	58.00	2	0.77	540	0.50
PPP1CC	0.0019609	×	√	1	0.3	2	2.87	0.01	0.35	5	58.00	2	0.77	540	0.50
**PRKCD**	NA	×	√	1	0	2	2.87	0.01	0.35	5	58.00	2	0.77	540	0.50
PRMT5	NA	×	√	1	0.2	1	3.78	0.00	0.26	6	5.00	1	0.65	0	0.00
RAD51C	NA	×	√	0.7	0	2	2.87	0.01	0.35	5	58.00	2	0.77	540	0.50
**RIPK1**	NA	×	√	1	0	2	2.87	0.01	0.35	5	58.00	2	0.77	540	0.50
SMAD1	NA	×	√	1	0.1	2	2.87	0.01	0.35	5	58.00	2	0.77	540	0.50
**TCEB1**	NA	×	√	1	0	2	2.87	0.01	0.35	5	58.00	2	0.77	540	0.50
TGFBR1	NA	×	√	1	0.2	2	2.87	0.01	0.35	5	58.00	2	0.77	540	0.50
TNNC1	0.046057	×	√	0.8	0	2	2.87	0.01	0.35	5	58.00	2	0.77	540	0.50

Supplement Table 4 Significant genes in the microarray meta-analysis of AF

EntrezID	Name	CombinedTstat	CombinedPval
3485	IGFBP2	7.078	1.45E-08
23245	ASTN2	-6.8552	3.54E-08
10170	DHRS9	6.7577	4.64E-08
2845	GPR22	-6.5137	1.82E-07
6123	RPL3L	6.4264	2.60E-07
79095	C9orf16	6.323	4.25E-07
10466	COG5	-6.2127	7.39E-07
23657	SLC7A11	-6.1763	8.15E-07
23452	ANGPTL2	6.1254	9.99E-07
22846	VASH1	5.8933	3.76E-06
10204	NUTF2	5.8704	3.93E-06
9470	EIF4E2	5.833	4.51E-06
1114	CHGB	5.8102	4.77E-06
4358	MPV17	5.7688	5.67E-06
64398	MPP5	-5.7541	5.77E-06
9252	RPS6KA5	-5.6416	9.85E-06
4282	MIF	5.6471	9.85E-06
112	ADCY6	5.6108	1.11E-05
1827	RCAN1	5.4554	2.56E-05
54476	RNF216	5.4439	2.59E-05
292	SLC25A5	5.4148	2.82E-05
2775	GNAO1	-5.4104	2.82E-05
9244	CRLF1	5.4038	2.82E-05
2098	ESD	-5.3314	3.87E-05
9145	SYNGR1	5.3314	3.87E-05
10160	FARP1	5.2929	4.33E-05
10570	DPYSL4	5.292	4.33E-05
25928	SOSTDC1	-5.2906	4.33E-05
3704	ITPA	5.2304	5.45E-05
1284	COL4A2	5.24	5.45E-05
116496	FAM129A	-5.2294	5.45E-05
64411	ARAP3	5.2151	5.70E-05
2687	GGT5	5.1965	6.12E-05
79630	C1orf54	5.1733	6.72E-05
5054	SERPINE1	5.1438	7.64E-05
1289	COL5A1	5.1303	7.99E-05
1056	CEL	-5.1104	8.63E-05
1282	COL4A1	5.0923	9.25E-05
367	AR	-5.0856	9.34E-05
5621	PRNP	-5.075	9.63E-05
2802	GOLGA3	5.0183	0.00011521
5998	RGS3	5.0271	0.00011521
11007	CCDC85B	5.0139	0.00011521
6425	SFRP5	-5.0146	0.00011521
23576	DDAH1	5.0173	0.00011521
655	BMP7	-5.016	0.00011521
23499	MACF1	-5.006	0.00011748
9446	GSTO1	4.9925	0.00012107
4855	NOTCH4	4.9922	0.00012107
10752	CHL1	-4.9808	0.00012249
5026	P2RX5	4.9784	0.00012249
2876	GPX1	4.9784	0.00012249
9158	FIBP	4.9629	0.00012777
8395	PIP5K1B	-4.9652	0.00012777
3418	IDH2	4.9506	0.00013299
55062	WIPI1	4.9481	0.00013299
533	ATP6V0B	4.9152	0.00015195
9317	PTER	-4.9169	0.00015195
55283	MCOLN3	-4.9014	0.00016027
2252	FGF7	-4.8977	0.0001606
2701	GJA4	4.8891	0.00016504
7040	TGFB1	4.8763	0.00016919
55061	SUSD4	-4.8747	0.00016919
23462	HEY1	4.8772	0.00016919

9628	RGS6	-4.8697	0.0001709
23024	PDZRN3	-4.8665	0.000171
64208	POPDC3	4.8607	0.00017352
6711	SPTBN1	-4.8486	0.00018165
11076	TPPP	-4.8427	0.00018185
23281	MTUS2	-4.843	0.00018185
9254	CACNA2D2	-4.8319	0.00018929
6196	RPS6KA2	4.8241	0.00019414
56901	NDUFA4L2	4.818	0.00019735
3925	STMN1	4.8007	0.00021118
2017	CTTN	4.7991	0.00021118
9260	PDLIM7	4.7939	0.00021391
9378	NRXN1	-4.7884	0.0002142
55654	TMEM127	4.7909	0.0002142
23195	MDN1	-4.7801	0.00022037
10019	SH2B3	4.7746	0.00022364
54436	SH3TC1	4.7671	0.00022929
7045	TGFBI	4.7608	0.00023371
3781	KCNN2	-4.7372	0.00025937
50509	COL5A3	4.7297	0.00026603
9232	PTTG1	4.7166	0.00027959
57146	TMEM159	4.7143	0.00027959
55076	TMEM45A	4.7124	0.00027959
5354	PLP1	-4.7001	0.00029358
26469	PTPN18	4.681	0.00031097
4900	NRGN	4.6793	0.00031097
1497	CTNS	4.6846	0.00031097
51282	SCAND1	4.6817	0.00031097
7450	VWF	4.6732	0.00031693
6453	ITSN1	-4.6688	0.00032021
50507	NOX4	4.6578	0.00033426
5699	PSMB10	4.6549	0.00033553
26578	OSTF1	4.6476	0.00034404
9445	ITM2B	-4.6392	0.0003511
5223	PGAM1	4.6395	0.0003511
3958	LGALS3	-4.6342	0.00035603
8292	COLQ	4.6191	0.00037544
1112	FOXN3	-4.6195	0.00037544
7474	WNT5A	-4.6148	0.00037957
7837	PXDN	4.603	0.00039786
1802	DPH2	4.5988	0.00040223
51706	CYB5R1	4.5962	0.00040243
1952	CELSR2	-4.5939	0.00040243
3306	HSPA2	4.5928	0.00040243
54480	CHPF2	4.5909	0.00040243
7078	TIMP3	-4.5762	0.00042
3131	HLF	-4.5794	0.00042
55790	CSGALNACT1	-4.5765	0.00042
6279	S100A8	4.571	0.00042691
23221	RHOBTB2	4.5663	0.00042899
392	ARHGAP1	4.5675	0.00042899
57645	POGK	-4.5528	0.00045352
8728	ADAM19	4.5436	0.00046978
419	ART3	-4.5366	0.00047924
6472	SHMT2	4.5358	0.00047924
7167	TPI1	4.5291	0.00048978
1729	DIAPH1	4.5271	0.00048978
1906	EDN1	4.5259	0.00048978
664	BNIP3	4.5209	0.00049748
9898	UBAP2L	4.5163	0.00050441
7293	TNFRSF4	4.511	0.00051292
2170	FABP3	4.5037	0.00052655
5214	PFKP	4.4959	0.00054195
3760	KCNJ3	-4.4763	0.00058957
54674	LRRN3	-4.4658	0.00060991
28984	RGCC	4.4657	0.00060991
6356	CCL11	4.4556	0.00063447

27089	UQCRQ	4.4464	0.00065722
51069	MRPL2	4.441	0.00066893
55729	ATF7IP	-4.4289	0.00070224
81606	LBH	4.427	0.00070336
6442	SGCA	4.4232	0.00071046
2119	ETV5	4.4163	0.00071279
2487	FRZB	4.4186	0.00071279
25854	FAM149A	-4.4164	0.00071279
10897	YIF1A	4.4186	0.00071279
6009	RHEB	-4.4088	0.00073262
262	AMD1	-4.4032	0.00073652
7041	TGFB1I1	4.406	0.00073652
79064	TMEM223	4.4031	0.00073652
79810	PTCD2	-4.4012	0.00073774
3839	KPNA3	-4.3968	0.00074758
5090	PBX3	-4.3943	0.00075127
5862	RAB2A	-4.392	0.000754
22820	COPG1	4.39	0.00075592
64837	KLC2	4.3817	0.00078008
4059	BCAM	4.3772	0.00079121
6747	SSR3	4.3696	0.00081391
256364	EML3	4.3653	0.00081503
2186	BPTF	-4.365	0.00081503
8824	CES2	4.3659	0.00081503
1278	COL1A2	4.3567	0.00084089
54187	NANS	4.3533	0.00084884
8543	LMO4	-4.3511	0.00085178
79633	FAT4	-4.3484	0.00085699
51304	ZDHHC3	4.3452	0.0008587
51450	PRRX2	4.3453	0.0008587
2280	FKBP1A	4.3417	0.00086211
79050	NOC4L	4.3425	0.00086211
3939	LDHA	4.3249	0.00091905
5437	POLR2H	4.3252	0.00091905
23144	ZC3H3	4.321	0.00092708
4542	MYO1F	4.3203	0.00092708
5265	SERPINA1	4.3182	0.00093025
5126	PCSK2	-4.314	0.00094263
4957	ODF2	4.3075	0.00095956
23423	TMED3	4.3081	0.00095956
163486	DENND1B	-4.3022	0.00097715
22885	ABLIM3	-4.2964	0.00099696
3276	PRMT1	4.2914	0.0010142
79874	RABEP2	4.2885	0.0010213
81533	ITFG1	-4.2872	0.0010214
11015	KDELR3	4.2852	0.0010249
22841	RAB11FIP2	-4.2807	0.00104
11237	RNF24	4.2767	0.0010528
9520	NPEPPS	-4.2705	0.0010668
598	BCL2L1	4.2701	0.0010668
57496	MKL2	-4.2704	0.0010668
120	ADD3	-4.2675	0.0010733
54715	RBFOX1	-4.2651	0.0010792
2691	GHRH	-4.2623	0.0010866
54855	FAM46C	-4.2581	0.0011013
80319	CXXC4	-4.2556	0.0011081
10402	ST3GAL6	4.2536	0.0011119
23633	KPNA6	4.2419	0.0011623
50515	CHST11	4.2413	0.0011623
3298	HSF2	-4.2369	0.0011728
4240	MFGE8	4.2375	0.0011728
1311	COMP	4.2186	0.0012654
55151	TMEM38B	-4.2169	0.0012684
9168	TMSB10	4.2055	0.0013277
1014	CDH16	4.2006	0.0013495
5337	PLD1	-4.1973	0.0013624
862	RUNX1T1	-4.195	0.0013692

23224	SYNE2	-4.1747	0.0014391
7058	THBS2	4.1757	0.0014391
9600	PITPNM1	4.1821	0.0014391
2268	FGR	4.1779	0.0014391
55107	ANO1	4.1762	0.0014391
54839	LRRC49	-4.1807	0.0014391
2357	FPR1	4.1764	0.0014391
10899	JTB	4.1755	0.0014391
4017	LOXL2	4.1713	0.0014541
23287	AGTPBP1	-4.1701	0.0014547
217	ALDH2	-4.1638	0.0014882
81578	COL21A1	4.153	0.0015528
8048	CSRP3	4.1414	0.0016255
64122	FN3K	4.14	0.0016276
10311	DSCR3	4.1333	0.0016533
55193	PBRM1	-4.1349	0.0016533
5494	PPM1A	-4.1332	0.0016533
51382	ATP6V1D	4.1305	0.0016655
6624	FSCN1	4.128	0.0016755
4854	NOTCH3	4.1251	0.0016815
7137	TNNI3	4.1253	0.0016815
1281	COL3A1	4.1223	0.0016943
7126	TNFAIP1	4.1168	0.001698
5567	PRKACB	-4.1164	0.001698
6182	MRPL12	4.1146	0.001698
22899	ARHGEF15	4.1198	0.001698
199	AIF1	4.1161	0.001698
54867	TMEM214	4.1162	0.001698
29100	TMEM208	4.1149	0.001698
64129	TINAGL1	4.1073	0.001745
1385	CREB1	-4.0926	0.0018508
54209	TREM2	4.0891	0.0018707
23304	UBR2	-4.0856	0.0018909
6396	SEC13	4.0809	0.0019211
8914	TIMELESS	4.0776	0.001924
1003	CDH5	4.0776	0.001924
1822	ATN1	4.0796	0.001924
5305	PIP4K2A	4.074	0.0019459
5501	PPP1CC	-4.0683	0.0019609
8277	TKTL1	-4.0694	0.0019609
25897	RNF19A	-4.0691	0.0019609
57763	ANKRA2	-4.0683	0.0019609
8624	PSMG1	4.0647	0.0019667
57142	RTN4	-4.0649	0.0019667
10971	YWHAQ	4.0666	0.0019667
2191	FAP	4.0625	0.0019773
2982	GUCY1A3	-4.0512	0.0020667
3714	JAG2	4.0399	0.0021518
54676	GTPBP2	4.0408	0.0021518
4920	ROR2	-4.0258	0.0022756
4879	NPPB	4.0248	0.0022756
28992	MACROD1	4.0201	0.0023119
5476	CTSA	4.0185	0.0023184
6652	SORD	-4.0157	0.0023373
5139	PDE3A	-4.0049	0.0024364
54922	RASIP1	4.0038	0.0024386
1622	DBI	3.9957	0.0025132
2026	ENO2	3.9936	0.0025206
90410	IFT20	3.9932	0.0025206
55244	SLC47A1	3.9896	0.0025493
51285	RASL12	-3.9886	0.0025504
2828	GPR4	3.9839	0.0025817
92140	MTDH	-3.9838	0.0025817
10567	RABAC1	3.9826	0.0025857
65979	PHACTR4	3.9802	0.0025943
2859	GPR35	3.98	0.0025943
54210	TREM1	3.9641	0.0027624

4915	NTRK2	-3.9621	0.0027759
6886	TAL1	3.9594	0.0027964
9454	HOMER3	3.9572	0.0027975
29091	STXBP6	-3.9577	0.0027975
10797	MTHFD2	3.9566	0.0027975
2	A2M	3.9525	0.002836
57185	NIPAL3	-3.9503	0.002845
79652	TMEM204	3.95	0.002845
158	ADSL	3.9486	0.0028503
25796	PGLS	3.9431	0.0028963
4674	NAP1L2	-3.9431	0.0028963
3454	IFNAR1	3.933	0.0029923
1140	CHRNB1	3.9332	0.0029923
7016	TESK1	3.9318	0.0029923
53838	C11orf24	3.9318	0.0029923
27430	MAT2B	-3.9272	0.0030394
6535	SLC6A8	3.9241	0.0030463
23451	SF3B1	-3.9241	0.0030463
6503	SLA	3.9247	0.0030463
5638	PRRG1	-3.922	0.0030511
27338	UBE2S	3.922	0.0030511
8409	UXT	3.9113	0.0031791
835	CASP2	3.9061	0.0032359
10460	TACC3	3.9032	0.0032636
26999	CYFIP2	-3.8978	0.0033266
1534	CYB561	-3.8947	0.0033452
4213	MEIS3P1	-3.8955	0.0033452
10519	CIB1	3.8919	0.0033632
51596	CUTA	3.891	0.0033632
57787	MARK4	3.891	0.0033632
6150	MRPL23	3.8894	0.0033732
22845	DOLK	3.8883	0.0033777
5701	PSMC2	-3.8842	0.0034004
8857	FCGBP	3.8849	0.0034004
79415	C17orf62	3.8858	0.0034004
537	ATP6AP1	3.8755	0.0035127
22918	CD93	3.8728	0.0035397
23002	DAAM1	-3.872	0.0035397
9728	SECISBP2L	-3.8609	0.0036932
4775	NFATC3	-3.8582	0.0037095
10467	ZNHIT1	3.8589	0.0037095
23185	LARP4B	-3.856	0.00373
59	ACTA2	-3.8535	0.0037491
1201	CLN3	3.8532	0.0037491
10970	CKAP4	3.8453	0.0038595
5902	RANBP1	3.8414	0.0039091
6696	SPP1	3.8353	0.0039806
5078	PAX4	3.8356	0.0039806
29095	ORMDL2	3.8346	0.0039806
5502	PPP1R1A	-3.8276	0.0040684
1454	CSNK1E	3.8279	0.0040684
3249	HPN	-3.8246	0.0041068
783	CACNB2	-3.8197	0.004139
29911	HOOK2	3.8195	0.004139
4015	LOX	3.8202	0.004139
211	ALAS1	3.82	0.004139
64761	PARP12	3.8113	0.0042667
9361	LONP1	3.8099	0.0042767
83480	PUS3	3.8086	0.0042859
9969	MED13	-3.8041	0.0043397
5029	P2RY2	3.804	0.0043397
56833	SLAMF8	3.8024	0.004352
92609	TIMM50	3.8018	0.004352
10763	NES	3.801	0.0043533
5530	PPP3CA	-3.7988	0.0043625
10072	DPP3	3.7995	0.0043625
26119	LDLRAP1	3.7982	0.0043625

5648	MASP1	3.7952	0.0044027
81876	RAB1B	3.7936	0.0044174
987	LRBA	-3.7927	0.0044202
64759	TNS3	-3.7906	0.0044438
2200	FBN1	-3.7874	0.0044747
8877	SPHK1	3.788	0.0044747
64710	NUCKS1	-3.7824	0.0045533
5837	PYGM	-3.7816	0.004554
23023	TMCC1	-3.7803	0.0045647
5166	PDK4	3.7778	0.0045975
1020	CDK5	3.7758	0.00462
79890	RIN3	3.7711	0.0046959
5523	PPP2R3A	-3.7685	0.0047313
8629	JRK	-3.7669	0.0047468
11098	PRSS23	3.7647	0.0047745
3678	ITGA5	3.758	0.0048808
6304	SATB1	-3.7563	0.0048808
1337	COX6A1	3.7556	0.0048808
7378	UPP1	3.7557	0.0048808
10362	HMG20B	3.7549	0.0048808
51177	PLEKHO1	3.7565	0.0048808
7138	TNNT1	3.7517	0.0048943
60493	FASTKD5	3.7524	0.0048943
10174	SORBS3	3.7526	0.0048943
5033	P4HA1	3.7514	0.0048943
51231	VRK3	3.7496	0.0049108
10287	RGS19	3.7492	0.0049108
9130	FAM50A	3.7464	0.0049266
4802	NFYC	-3.7456	0.0049266
4837	NNMT	3.746	0.0049266
9846	GAB2	3.7463	0.0049266
7060	THBS4	3.744	0.0049452
23263	MCF2L	3.7415	0.0049796
8668	EIF3I	3.7395	0.0050064
6793	STK10	3.7349	0.0050852
2149	F2R	3.7326	0.0051168
9791	PTDSS1	-3.7304	0.0051462
80852	GRIP2	3.7298	0.0051462
871	SERPINH1	3.7257	0.0052179
3779	KCNMB1	3.7199	0.0052805
79143	MBOAT7	3.7217	0.0052805
10252	SPRY1	3.72	0.0052805
91949	COG7	3.7205	0.0052805
23522	KAT6B	-3.7123	0.0054
5565	PRKAB2	-3.7135	0.0054
3752	KCND3	-3.7123	0.0054
4720	NDUFS2	3.7059	0.0054944
5261	PHKG2	3.7067	0.0054944
8270	LAGE3	3.7059	0.0054944
1288	COL4A6	-3.6999	0.0056106
29109	FHOD1	3.6929	0.0057533
81575	APOLD1	3.6885	0.0058385
9853	RUSC2	3.6878	0.0058388
4162	MCAM	3.6868	0.0058457
124583	CANT1	3.6859	0.0058511
6781	STC1	3.6824	0.005917
6262	RYR2	-3.6809	0.0059292
23405	DICER1	-3.6799	0.0059292
10312	TCIRG1	3.6806	0.0059292
2257	FGF12	-3.6773	0.0059762
55630	SLC39A4	3.6737	0.0060445
23043	TNIK	-3.6713	0.0060852
79180	EFHD2	3.6666	0.0061842
5087	PBX1	-3.6643	0.0062231
6676	SPAG4	3.6582	0.0063581
65108	MARCKSL1	3.6531	0.0064533
10396	ATP8A1	-3.6536	0.0064533

4430	MYO1B	3.6518	0.0064691
9013	TAF1C	3.6504	0.0064746
29775	CARD10	3.6503	0.0064746
83443	SF3B5	3.6492	0.0064867
427	ASAH1	3.6446	0.0065742
1293	COL6A3	3.6445	0.0065742
51226	COPZ2	3.6414	0.006576
967	CD63	3.6412	0.006576
24140	FTSJ1	3.6419	0.006576
92689	FAM114A1	3.6437	0.006576
55748	CNDP2	3.6412	0.006576
2201	FBN2	3.6385	0.006631
28965	SLC27A6	-3.6363	0.0066707
3985	LIMK2	3.6331	0.0067242
1464	CSPG4	3.633	0.0067242
637	BID	3.6317	0.0067428
6297	SALL2	-3.6282	0.0068008
53615	MBD3	3.6286	0.0068008
1912	PHC2	3.627	0.0068169
5355	PLP2	3.6258	0.0068315
8260	NAA10	3.6239	0.0068664
157697	ERICH1	3.6212	0.0069014
2219	FCN1	3.6207	0.0069014
5714	PSMD8	3.6209	0.0069014
4215	MAP3K3	3.6195	0.0069176
347902	AMIGO2	-3.6157	0.0069867
6697	SPR	3.6162	0.0069867
6523	SLC5A1	-3.6122	0.0070652
5154	PDGFA	3.6077	0.007172
55603	FAM46A	-3.6065	0.007188
4835	NQO2	3.6052	0.0072074
51020	HDDC2	-3.6045	0.0072109
23464	GCAT	3.6018	0.0072686
51585	PCF11	-3.6003	0.0072788
79913	ACTR5	3.6007	0.0072788
730	C7	-3.5957	0.0073757
10212	DDX39A	3.5951	0.0073757
5178	PEG3	-3.595	0.0073757
2009	EML1	-3.59	0.0075034
7707	ZNF148	-3.5829	0.0076903
5019	OXCT1	3.5814	0.0077014
10652	YKT6	3.582	0.0077014
23166	STAB1	3.5795	0.0077389
8895	CPNE3	-3.5784	0.0077549
26064	RAI14	3.5775	0.0077626
3265	HRAS	3.5757	0.0077841
51105	PHF20L1	-3.5756	0.0077841
63910	SLC17A9	3.575	0.0077858
284325	C19orf54	3.5713	0.0078796
1525	CXADR	-3.5672	0.0079556
253980	KCTD13	3.5675	0.0079556
5654	HTRA1	3.567	0.0079556
10513	APPBP2	-3.5655	0.0079845
23475	QPRT	-3.5635	0.0080264
2066	ERBB4	-3.5628	0.0080308
57099	AVEN	3.561	0.0080671
51294	PCDH12	3.5601	0.0080785
123803	NTAN1	3.5595	0.0080785
3309	HSPA5	-3.5588	0.0080822
128	ADH5	-3.5557	0.0081426
64091	POPDC2	3.5561	0.0081426
11245	GPR176	3.5549	0.0081506
1277	COL1A1	3.5501	0.0082643
50863	NTM	-3.5505	0.0082643
2617	GARS	3.5481	0.008311
27130	INVS	3.547	0.008326
6934	TCF7L2	-3.5438	0.0084114

212	ALAS2	3.5431	0.0084145
823	CAPN1	-3.5421	0.0084298
6484	ST3GAL4	3.5411	0.0084427
81848	SPRY4	3.5364	0.0085759
10488	CREB3	3.5343	0.0086268
26156	RSL1D1	-3.5323	0.0086733
334	APLP2	-3.5305	0.008715
80303	EFHD1	3.5286	0.008742
1636	ACE	3.528	0.008742
1347	COX7A2	3.5282	0.008742
23203	PMPCA	3.5248	0.0088272
1285	COL4A3	-3.5234	0.0088557
381	ARF5	3.5208	0.0089265
79177	ZNF576	3.5165	0.0090536
3140	MR1	3.5138	0.0091265
782	CACNB1	3.5127	0.0091437
51293	CD320	3.5119	0.0091536
23353	SUN1	3.5105	0.0091722
1777	DNASE2	3.5103	0.0091722
9792	SERTAD2	-3.508	0.0092273
91373	UAP1L1	3.5076	0.0092273
10390	CEPT1	3.5056	0.0092756
64943	NT5DC2	-3.5043	0.0093026
4521	NUDT1	3.5035	0.009311
8718	TNFRSF25	3.5023	0.0093353
9162	DGKI	3.4999	0.0093505
80781	COL18A1	3.4997	0.0093505
84061	MAGT1	-3.5005	0.0093505
64781	CERK	3.5004	0.0093505
6558	SLC12A2	3.4987	0.0093651
813	CALU	3.4952	0.0094388
2995	GYPC	3.495	0.0094388
1511	CTSG	3.4957	0.0094388
4016	LOXL1	3.494	0.0094573
23597	ACOT9	3.4917	0.00952
8027	STAM	-3.4899	0.0095662
64131	XYLT1	3.4892	0.0095718
57538	ALPK3	3.4886	0.0095748
5095	PCCA	-3.4864	0.0096324
55366	LGR4	-3.485	0.009666
63935	PCIF1	3.4834	0.0097022
633	BGN	3.4795	0.0098264
51409	HEMK1	3.478	0.0098418
5872	RAB13	3.4785	0.0098418
5805	PTS	3.4736	0.0099872
84705	GTPBP3	3.471	0.010064
6159	RPL29	3.4689	0.010123
7518	XRCC4	3.4656	0.01023
80230	RUFY1	3.4633	0.010296
4604	MYBPC1	-3.4593	0.010429
4752	NEK3	3.4587	0.010432
89797	NAV2	3.4582	0.010433
3852	KRT5	3.4554	0.010456
7019	TFAM	-3.4544	0.010456
23414	ZFPM2	-3.4558	0.010456
3020	H3F3A	-3.4553	0.010456
9414	TJP2	-3.4545	0.010456
83483	PLVAP	3.4539	0.010456
10025	MED16	3.4565	0.010456
399664	MEX3D	3.4534	0.010458
85236	HIST1H2BK	-3.4521	0.010476
51042	ZNF593	3.4519	0.010476
27147	DENND2A	-3.4499	0.010536
5376	PMP22	-3.4469	0.010633
978	CDA	3.443	0.010765
1950	EGF	-3.4423	0.010773
22864	R3HDM2	3.4348	0.011016

3818	KLKB1	-3.4348	0.011016
4830	NME1	3.4353	0.011016
114876	OSBPL1A	-3.4325	0.011087
51604	PIGT	3.4308	0.011137
58494	JAM2	-3.4297	0.011159
23131	GPATCH8	-3.429	0.01117
81609	SNX27	-3.4282	0.011182
2192	FBLN1	-3.4251	0.011254
7851	MALL	3.425	0.011254
6533	SLC6A6	3.4249	0.011254
9638	FEZ1	-3.4241	0.011262
2274	FHL2	3.4237	0.011262
580	BARD1	-3.4214	0.011277
22822	PHLDA1	3.4214	0.011277
10539	GLRX3	3.4224	0.011277
56965	PARP6	3.4218	0.011277
8301	PICALM	3.4181	0.011393
7372	UMPS	3.4145	0.011525
8509	NDST2	3.4139	0.01153
27235	COQ2	3.4128	0.011554
1302	COL11A2	3.4115	0.011586
372	ARCN1	3.4076	0.011735
9353	SLIT2	-3.4062	0.011775
112399	EGLN3	3.4023	0.011923
81628	TSC22D4	-3.4017	0.011925
29896	TRA2A	-3.401	0.011937
9322	TRIP10	3.3998	0.011945
25907	TMEM158	3.3999	0.011945
439	ASNA1	3.3972	0.012039
51203	NUSAP1	3.396	0.012068
9077	DIRAS3	3.3937	0.012143
55131	RBM28	3.3933	0.012143
60682	SMAP1	3.3929	0.012143
1605	DAG1	-3.3901	0.012148
8153	RND2	-3.3904	0.012148
79716	NPEPL1	-3.3909	0.012148
10250	SRRM1	3.3909	0.012148
1674	DES	3.3916	0.012148
373156	GSTK1	3.3899	0.012148
79004	CUEDC2	3.3885	0.012166
2901	GRIK5	-3.3886	0.012166
3725	JUN	-3.3869	0.012215
3371	TNC	3.3834	0.012288
9409	PEX16	3.3836	0.012288
5516	PPP2CB	-3.3835	0.012288
4627	MYH9	3.3837	0.012288
6738	TROVE2	-3.3823	0.012318
1609	DGKQ	3.3815	0.01233
79875	THSD4	-3.381	0.012333
2868	GRK4	3.3802	0.012345
7135	TNNI1	-3.379	0.01236
254102	EHBP1L1	3.3789	0.01236
55379	LRRC59	3.3761	0.012469
1890	TYMP	3.3755	0.012475
2070	EYA4	-3.3714	0.01264
23328	SASH1	-3.37	0.012659
56993	TOMM22	3.3704	0.012659
8818	DPM2	3.369	0.012683
54923	LIME1	3.3682	0.012702
79173	C19orf57	3.3665	0.012759
24147	FJX1	3.3656	0.012779
51318	MRPL35	3.3618	0.012931
4218	RAB8A	3.3609	0.012945
4222	MEOX1	3.3606	0.012945
8165	AKAP1	-3.3555	0.013149
22849	CPEB3	-3.3554	0.013149
79852	EPHX3	3.3517	0.013282

706	TSPO	3.3516	0.013282
10195	ALG3	3.3507	0.013308
81576	CCDC130	3.3498	0.013329
79778	MICALL2	3.3476	0.013368
7504	XK	-3.3479	0.013368
54896	PQLC2	3.3476	0.013368
7102	TSPAN7	-3.342	0.013616
10021	HCN4	-3.3383	0.013774
3099	HK2	3.3371	0.013791
22898	DENND3	3.3372	0.013791
2677	GGCX	3.335	0.013874
4053	LTBP2	3.3334	0.013919
22920	KIFAP3	3.3332	0.013919
5585	PKN1	3.3298	0.014064
7286	TUFT1	-3.327	0.014184
22992	KDM2A	-3.3264	0.014193
55827	DCAF6	-3.3254	0.01422
25855	BRMS1	3.3249	0.014222
23480	SEC61G	3.3208	0.014407
10094	ARPC3	3.3201	0.014423
23122	CLASP2	-3.3185	0.01448
60559	SPCS3	3.3168	0.014545
3310	HSPA6	3.3151	0.014611
5049	PAFAH1B2	3.3144	0.014624
4255	MGMT	3.3098	0.014794
2596	GAP43	3.3098	0.014794
27068	PPA2	-3.3106	0.014794
79754	ASB13	3.3093	0.014797
1725	DHPS	3.3087	0.014808
4688	NCF2	3.308	0.014824
6915	TBXA2R	3.304	0.014964
81552	VOPP1	3.304	0.014964
1601	DAB2	-3.3047	0.014964
5999	RGS4	3.2996	0.015178
1004	CDH6	3.2965	0.015319
79080	CCDC86	3.296	0.015325
3250	HPR	-3.2951	0.015348
5927	KDM5A	-3.2928	0.015376
1439	CSF2RB	3.2939	0.015376
9447	AIM2	3.2933	0.015376
26051	PPP1R16B	3.2932	0.015376
9844	ELMO1	3.2921	0.015393
157567	ANKRD46	-3.2888	0.015541
395	ARHGAP6	3.2885	0.015541
9590	AKAP12	-3.2857	0.015672
7559	ZNF12	-3.2838	0.015752
57820	CCNB1IP1	3.2794	0.015977
130814	PQLC3	3.2781	0.016028
10766	TOB2	-3.2768	0.016078
5003	SLC22A18AS	3.2734	0.016226
7416	VDAC1	3.2733	0.016226
2997	GYS1	3.2702	0.016328
9852	EPM2AIP1	-3.2704	0.016328
51015	ISOC1	-3.2703	0.016328
5295	PIK3R1	-3.2692	0.016347
165	AEBP1	3.269	0.016347
9266	CYTH2	3.2676	0.016406
1499	CTNNB1	3.2658	0.016471
55974	SLC50A1	3.2644	0.016471
23647	ARFIP2	3.2643	0.016471
29015	SLC43A3	3.2651	0.016471
51021	MRPS16	3.2656	0.016471
1353	COX11	-3.2638	0.016474
5379	PMS2P1	3.2622	0.016544
58986	TMEM8A	3.2613	0.01657
3652	IPP	-3.2594	0.016658
374291	NDUFS7	3.2584	0.016667

7264	TSTA3	3.2584	0.016667
5947	RBP1	-3.2569	0.016731
593	BCKDHA	3.2563	0.016736
53405	CLIC5	-3.2543	0.016736
25813	SAMM50	3.2554	0.016736
3654	IRAK1	3.2546	0.016736
23300	ATMIN	-3.2558	0.016736
23261	CAMTA1	3.2543	0.016736
3931	LCAT	3.2535	0.016758
51154	MRTO4	3.2522	0.01681
2788	GNG7	3.2509	0.016863
657	BMPR1A	-3.2471	0.017012
80895	ILKAP	3.2472	0.017012
11329	STK38	3.2477	0.017012
10607	TBL3	3.2455	0.017081
6654	SOS1	-3.244	0.017146
527	ATP6V0C	3.2427	0.017199
6283	S100A12	3.2414	0.017211
55140	ELP3	3.2417	0.017211
138046	RALYL	-3.2413	0.017211
333	APLP1	3.2404	0.017241
9180	OSMR	3.24	0.017241
23743	BHMT2	-3.2378	0.017345
10090	UST	-3.2358	0.017444
64100	ELSPBP1	3.2349	0.017471
54584	GNB1L	3.2341	0.017495
8832	CD84	3.2328	0.017528
1428	CRYM	3.2329	0.017528
91369	ANKRD40	3.2322	0.01754
2534	FYN	-3.2299	0.017629
1019	CDK4	3.23	0.017629
4878	NPPA	3.2291	0.017654
2118	ETV4	3.2286	0.017656
9619	ABCG1	3.2267	0.01775
1063	CENPF	3.2256	0.017784
1066	CES1	-3.2254	0.017784
81619	TSPAN14	3.2213	0.018014
64210	MMS19	3.2206	0.018029
2987	GUK1	3.2178	0.018184
790	CAD	3.2161	0.018216
5216	PFN1	3.2163	0.018216
63875	MRPL17	3.2166	0.018216
10953	TOMM34	3.2154	0.01823
55030	FBXO34	-3.2137	0.018289
10095	ARPC1B	3.2137	0.018289
23125	CAMTA2	3.2128	0.01832
58477	SRPRB	3.2119	0.018353
7070	THY1	3.2111	0.018377
911	CD1C	3.2098	0.018433
10477	UBE2E3	-3.2081	0.018466
1409	CRYAA	3.2088	0.018466
362	AQP5	3.2082	0.018466
10825	NEU3	3.2074	0.018486
92856	IMP4	3.2061	0.018545
8745	ADAM23	-3.2045	0.01862
2210	FCGR1B	3.2037	0.018647
3162	HMOX1	3.2028	0.018655
204851	HIPK1	-3.2029	0.018655
4684	NCAM1	-3.201	0.01872
60528	ELAC2	-3.201	0.01872
55317	AP5S1	3.1952	0.019075
55000	TUG1	-3.1942	0.019116
4318	MMP9	3.1933	0.01915
2120	ETV6	3.1894	0.019352
63906	GPATCH3	3.1896	0.019352
3909	LAMA3	3.1886	0.019353
10345	TRDN	-3.1887	0.019353

23118	TAB2	-3.1873	0.019409
168544	ZNF467	3.187	0.019409
3044	HBBP1	3.1851	0.019497
26253	CLEC4E	3.1847	0.019497
84900	RNFT2	3.1845	0.019497
23411	SIRT1	-3.1825	0.019603
9616	RNF7	3.1808	0.019696
730092	RRN3P1	-3.1798	0.019738
80306	MED28	-3.1789	0.01977
51162	EGFL7	3.1785	0.019771
3055	HCK	3.1753	0.019964
3708	ITPR1	-3.1728	0.020082
23632	CA14	-3.1731	0.020082
4046	LSP1	3.1714	0.020151
7226	TRPM2	3.1698	0.020211
57228	SMAGP	3.1701	0.020211
6867	TACC1	3.1689	0.020246
146712	B3GNTL1	3.1682	0.020271
10988	METAP2	-3.1667	0.020318
4329	ALDH6A1	3.1668	0.020318
8786	RGS11	3.1644	0.020356
10799	RPP40	3.1657	0.020356
80219	COQ10B	3.1646	0.020356
7852	CXCR4	3.1654	0.020356
4701	NDUFA7	3.1635	0.020356
4330	MN1	-3.1637	0.020356
51311	TLR8	3.1636	0.020356
3632	INPP5A	3.1612	0.020492
56616	DIABLO	3.1588	0.020631
55954	ZMAT5	3.158	0.020657
8175	SF3A2	3.1577	0.020657
9851	KIAA0753	3.1568	0.020665
29116	MYLIP	-3.1569	0.020665
8602	NOP14	3.1549	0.020771
55743	CHFR	3.1538	0.020828
4983	OPHN1	3.1532	0.020844
7716	VEZF1	-3.1488	0.021132
283871	PGP	3.147	0.02123
10230	NBR2	-3.146	0.021253
10865	ARID5A	3.1461	0.021253
54664	TMEM106B	-3.1444	0.021338
56882	CDC42SE1	3.144	0.021341
866	SERPINA6	3.1427	0.021408
56924	PAK6	-3.1418	0.021418
84617	TUBB6	3.1419	0.021418
3625	INHBB	3.141	0.021451
9188	DDX21	3.139	0.021566
8204	NRIP1	-3.1376	0.021638
1634	DCN	-3.1369	0.021638
2275	FHL3	3.137	0.021638
10987	COPS5	-3.1365	0.021642
7108	TM7SF2	-3.1355	0.021688
9201	DCLK1	-3.1345	0.021737
7355	SLC35A2	3.1332	0.021804
1175	AP2S1	3.1328	0.021809
9645	MICAL2	3.1313	0.021869
4924	NUCB1	3.1312	0.021869
6605	SMARCE1	-3.1296	0.021933
54840	APTX	3.1298	0.021933
4888	NPY6R	3.1284	0.021993
54583	EGLN1	3.1278	0.022016
6853	SYN1	3.1271	0.022037
5932	RBBP8	3.1247	0.022144
2778	GNAS	-3.1246	0.022144
65980	BRD9	3.125	0.022144
9258	MFHAS1	-3.1226	0.022263
6809	STX3	3.1194	0.022446

80152	CENPT	3.1191	0.022446
23092	ARHGAP26	-3.1192	0.022446
3956	LGALS1	3.1179	0.022511
8490	RGS5	3.116	0.022607
8643	PTCH2	3.1159	0.022607
2745	GLRX	3.1147	0.022674
29098	RANGRF	3.1139	0.022709
729	C6	-3.1121	0.022817
5270	SERPINE2	3.1107	0.0229
55096	EBLN2	3.11	0.022925
5833	PCYT2	3.1092	0.022959
23549	DNPEP	3.1068	0.023117
26037	SIPA1L1	3.1056	0.023178
84818	IL17RC	3.1042	0.023261
29883	CNOT7	-3.101	0.023486
8412	BCAR3	-3.0983	0.023644
5016	OVGP1	3.0984	0.023644
8115	TCL1A	3.0972	0.023702
7157	TP53	3.0947	0.02385
54477	PLEKHA5	-3.0946	0.02385
3759	KCNJ2	3.0928	0.02387
2528	FUT6	3.0927	0.02387
1387	CREBBP	-3.0923	0.02387
79573	TTC13	3.0922	0.02387
6748	SSR4	3.0935	0.02387
9919	SEC16A	3.0933	0.02387
2034	EPAS1	-3.0897	0.024048
54829	ASPN	3.0877	0.024175
308	ANXA5	3.087	0.024203
23112	TNRC6B	-3.0866	0.024207
2771	GNAI2	3.0857	0.024256
5347	PLK1	3.0846	0.024312
10577	NPC2	3.0826	0.024423
6183	MRPS12	3.0827	0.024423
23099	ZBTB43	-3.0818	0.024456
26472	PPP1R14B	3.0813	0.024468
54555	DDX49	3.0799	0.024555
55559	HAUS7	3.0794	0.024566
642	BLMH	3.0756	0.024762
3339	HSPG2	3.0763	0.024762
5238	PGM3	3.0757	0.024762
9230	RAB11B	3.0756	0.024762
8228	PNPLA4	-3.0744	0.02483
2181	ACSL3	-3.0736	0.02487
51188	SS18L2	3.0733	0.024871
116832	RPL39L	3.0696	0.025147
6588	SLN	-3.0691	0.025159
3547	IGSF1	-3.068	0.025227
51635	DHRS7	-3.0668	0.02529
23190	UBXN4	-3.0655	0.025341
27102	EIF2AK1	3.0657	0.025341
241	ALOX5AP	3.0644	0.025345
7533	YWHAH	3.064	0.025345
2242	FES	3.0638	0.025345
51073	MRPL4	3.0641	0.025345
64174	DPEP2	3.0649	0.025345
9918	NCAPD2	3.0628	0.025395
23396	PIP5K1C	3.0606	0.02555
26953	RANBP6	-3.0602	0.02556
2027	ENO3	3.0573	0.025774
64975	MRPL41	3.0554	0.025913
5934	RBL2	-3.054	0.025998
22904	SBNO2	3.0527	0.026083
23264	ZC3H7B	-3.0513	0.026177
4200	ME2	-3.0502	0.026237
79882	ZC3H14	-3.0496	0.026247
11141	IL1RAPL1	-3.0494	0.026247

26574	AATF	3.0485	0.026303
7048	TGFBR2	-3.0479	0.026323
5479	PPIB	3.0473	0.026346
7410	VAV2	3.046	0.02643
55127	HEATR1	-3.0445	0.026526
23036	ZNF292	-3.0441	0.026534
6385	SDC4	3.0437	0.026543
22872	SEC31A	3.0427	0.026601
81614	NIPA2	3.0418	0.026642
8991	SELENBP1	-3.0385	0.026892
27344	PCSK1N	3.0377	0.026892
4811	NID1	3.0382	0.026892
30827	CXXC1	3.0378	0.026892
55466	DNAJA4	3.0354	0.027036
4722	NDUFS3	3.0354	0.027036
3074	HEXB	3.0344	0.027091
1964	EIF1AX	-3.0308	0.027384
23639	LRRC6	3.0275	0.027657
55788	LMBRD1	-3.0249	0.027867
2099	ESR1	-3.0222	0.02808
9527	GOSR1	-3.0204	0.02814
553	AVPR1B	3.0202	0.02814
54517	PUS7	3.0208	0.02814
2648	KAT2A	3.0207	0.02814
2041	EPHA1	3.0188	0.028238
2131	EXT1	3.0181	0.028275
80205	CHD9	-3.0171	0.028337
1046	CDX4	3.0155	0.028423
2954	GSTZ1	3.0157	0.028423
5770	PTPN1	3.0134	0.028588
22902	RUFY3	-3.012	0.028653
23463	ICMT	3.0121	0.028653
4097	MAFG	3.0111	0.028675
26137	ZBTB20	-3.0113	0.028675
687	KLF9	-3.0093	0.02877
10817	FRS3	3.0091	0.02877
7791	ZYX	3.0093	0.02877
56255	TMX4	-3.0037	0.029087
3073	HEXA	-3.0048	0.029087
9903	KLHL21	-3.0037	0.029087
9695	EDEM1	3.0035	0.029087
50624	CUZD1	3.0031	0.029087
81792	ADAMTS12	3.0037	0.029087
6868	ADAM17	3.0033	0.029087
81553	FAM49A	3.0042	0.029087
29888	STRN4	2.9996	0.029354
51647	FAM96B	2.9998	0.029354
7127	TNFAIP2	2.9982	0.029462
57178	ZMIZ1	-2.9978	0.02947
7375	USP4	2.9951	0.029667
6241	RRM2	2.9953	0.029667
4820	NKTR	-2.9938	0.029759
10629	TAF6L	2.9922	0.029825
26509	MYOF	2.9927	0.029825
1104	RCC1	2.9921	0.029825
79719	AAGAB	2.9915	0.029855
80204	FBXO11	-2.9908	0.029893
55186	SLC25A36	-2.9872	0.030179
93664	CADPS2	-2.9873	0.030179
6455	SH3GL1	2.9863	0.030238
57333	RCN3	2.9849	0.030344
51330	TNFRSF12A	2.9839	0.03041
5108	PCM1	-2.9831	0.030458
55660	PRPF40A	-2.9812	0.030605
8850	KAT2B	-2.981	0.030605
9491	PSMF1	2.9806	0.030607
7076	TIMP1	2.9795	0.030687

221078	NSUN6	-2.9791	0.030692
54662	TBC1D13	2.9784	0.030728
54968	TMEM70	-2.9754	0.030994
5425	POLD2	2.9738	0.031125
65055	REEP1	-2.9724	0.031207
1909	EDNRA	-2.9725	0.031207
9450	LY86	2.971	0.031309
79628	SH3TC2	-2.9705	0.031332
641	BLM	-2.9682	0.03153
9040	UBE2M	2.966	0.031687
2669	GEM	2.9663	0.031687
22974	TPX2	2.9632	0.031942
10816	SPINT3	2.9626	0.031974
949	SCARB1	2.9608	0.032045
4664	NAB1	-2.9606	0.032045
10686	CLDN16	2.9612	0.032045
4306	NR3C2	-2.9611	0.032045
6402	SELL	2.9601	0.032068
58189	WFDC1	2.9595	0.032093
287	ANK2	-2.9571	0.032244
3581	IL9R	2.9574	0.032244
1072	CFL1	2.9571	0.032244
373	TRIM23	-2.9555	0.03238
6617	SNAPC1	-2.9531	0.032531
3718	JAK3	2.9531	0.032531
65244	SPATS2	2.9534	0.032531
10616	RBCK1	2.9522	0.032587
6601	SMARCC2	-2.9503	0.032746
4791	NFKB2	2.9501	0.032746
51411	BIN2	2.9475	0.032823
8500	PPFIA1	2.9475	0.032823
4140	MARK3	-2.9481	0.032823
435	ASL	2.9479	0.032823
728392	LOC728392	2.9485	0.032823
83449	PMFBP1	2.9482	0.032823
5789	PTPRD	-2.9468	0.032862
3417	IDH1	-2.9461	0.032904
54472	TOLLIP	2.9445	0.033028
8355	HIST1H3G	2.9443	0.033028
11240	PADI2	2.9433	0.033099
2908	NR3C1	-2.9408	0.033262
2135	EXTL2	-2.9408	0.033262
23604	DAPK2	-2.941	0.033262
9535	GMFG	2.9396	0.03336
2246	FGF1	2.9379	0.033456
6746	SSR2	2.9377	0.033456
81539	SLC38A1	-2.938	0.033456
1859	DYRK1A	-2.9342	0.033803
3594	IL12RB1	2.9335	0.033846
23428	SLC7A8	2.933	0.033864
55658	RNF126	2.9325	0.033883
28511	NKIRAS2	2.9312	0.033998
3663	IRF5	2.9298	0.034117
6357	CCL13	2.9278	0.034294
10842	PPP1R17	2.9274	0.034312
3291	HSD11B2	2.9267	0.034341
55651	NHP2	2.9262	0.034341
23034	SAMD4A	2.9262	0.034341
4643	MYO1E	2.9255	0.034349
4635	MYL4	-2.9257	0.034349
1234	CCR5	2.9248	0.034383
23473	CAPN7	-2.9245	0.034389
811	CALR	-2.9232	0.034457
51167	CYB5R4	2.9235	0.034457
2794	GNL1	-2.922	0.034565
134	ADORA1	-2.9212	0.034612
23129	PLXND1	2.9191	0.034709

6281	S100A10	-2.9193	0.034709
6320	CLEC11A	2.9196	0.034709
54796	BNC2	-2.9198	0.034709
84830	ADTRP	2.9178	0.034824
30835	CD209	2.9151	0.035084
2580	GAK	2.9145	0.035126
57326	PBXIP1	-2.914	0.035141
6595	SMARCA2	-2.9128	0.035239
1823	DSC1	-2.911	0.035373
7098	TLR3	-2.9111	0.035373
2065	ERBB3	-2.9097	0.035444
9516	LITAF	2.9082	0.035444
6850	SYK	2.9083	0.035444
9710	KIAA0355	2.9079	0.035444
9459	ARHGEF6	2.9098	0.035444
22993	HMGXB3	2.9088	0.035444
8847	DLEU2	-2.908	0.035444
24141	LAMP5	2.9085	0.035444
3383	ICAM1	2.9066	0.035558
80310	PDGFD	2.9059	0.035603
1611	DAP	2.905	0.035668
10553	HTATIP2	2.9042	0.035735
4199	ME1	-2.9027	0.035865
8209	C21orf33	2.9023	0.035872
9975	NR1D2	-2.9004	0.036057
25844	YIPF3	2.8991	0.036177
2021	ENDOG	2.898	0.036263
22930	RAB3GAP1	-2.8959	0.036367
80741	LY6G5C	2.8968	0.036367
55315	SLC29A3	2.8959	0.036367
26279	PLA2G2D	2.8962	0.036367
8527	DGKD	2.8953	0.036399
441024	MTHFD2L	2.8942	0.036497
1848	DUSP6	2.8933	0.036567
10129	FRY	-2.8927	0.036577
54927	CHCHD3	2.8926	0.036577
6509	SLC1A4	2.8918	0.036603
10288	LILRB2	2.892	0.036603
2958	GTF2A2	-2.8914	0.036615
51340	CRNKL1	-2.8903	0.0367
29928	TIMM22	2.8896	0.036748
54704	PDP1	-2.8873	0.036867
30819	KCNIP2	-2.8879	0.036867
2941	GSTA4	-2.8871	0.036867
55702	CCDC94	2.8873	0.036867
23533	PIK3R5	2.8878	0.036867
8498	RANBP3	-2.8848	0.037065
8464	SUPT3H	2.8851	0.037065
2634	GBP2	-2.8842	0.037095
54910	SEMA4C	2.884	0.037095
23545	ATP6V0A2	2.8835	0.037114
353	APRT	2.8827	0.037167
2060	EPS15	-2.8813	0.037303
23507	LRRC8B	2.8797	0.037319
25841	ABTB2	2.8802	0.037319
8675	STX16	-2.8797	0.037319
5266	PI3	2.8805	0.037319
3385	ICAM3	2.8806	0.037319
51117	COQ4	2.8789	0.037381
90956	ADCK2	2.8778	0.03747
4149	MAX	-2.8767	0.037512
79096	C11orf49	2.8767	0.037512
11000	SLC27A3	2.8766	0.037512
83604	TMEM47	-2.8754	0.037615
3579	CXCR2	2.8738	0.037732
10494	STK25	2.8735	0.037732
56950	SMYD2	2.874	0.037732

867	CBL	2.8728	0.037786
9857	CEP350	-2.8694	0.03816
92346	C1orf105	-2.8686	0.038215
9179	AP4M1	2.866	0.038503
11140	CDC37	2.8656	0.038511
4689	NCF4	2.8653	0.038514
22881	ANKRD6	-2.8645	0.038531
51094	ADIPOR1	2.8648	0.038531
359	AQP2	2.8634	0.0386
23536	ADAT1	2.8637	0.0386
10884	MRPS30	-2.8625	0.038668
84549	MAK16	2.8616	0.038746
314	AOC2	2.8599	0.038885
64897	C12orf43	2.86	0.038885
7360	UGP2	-2.8584	0.039026
2245	FGD1	2.8578	0.039066
6294	SAFB	-2.8525	0.039314
4234	METTL1	2.8509	0.039314
23089	PEG10	-2.8509	0.039314
6530	SLC6A2	2.8508	0.039314
79159	NOL12	2.8549	0.039314
397	ARHGDIB	2.8532	0.039314
23363	OBSL1	-2.852	0.039314
29992	PILRA	2.8536	0.039314
6678	SPARC	2.8503	0.039314
5300	PIN1	2.8547	0.039314
80742	PRR3	2.8523	0.039314
5393	EXOSC9	2.8528	0.039314
5531	PPP4C	2.8545	0.039314
2874	GPS2	2.8513	0.039314
26608	TBL2	2.8506	0.039314
164656	TMPRSS6	2.8523	0.039314
55222	LRRC20	-2.8545	0.039314
79772	MCTP1	2.8527	0.039314
126321	MFSD12	2.8542	0.039314
10101	NUBP2	2.8498	0.039336
3456	IFNB1	2.8492	0.03934
8736	MYOM1	2.8495	0.03934
64755	C16orf58	2.8484	0.039404
25825	BACE2	2.8481	0.039408
25842	ASF1A	-2.8473	0.039472
7298	TYMS	2.8467	0.039512
1025	CDK9	2.8458	0.039549
402	ARL2	2.8458	0.039549
7072	TIA1	-2.845	0.039611
56937	PMEPA1	2.8448	0.039611
841	CASP8	2.8437	0.039703
51070	NOSIP	2.843	0.039756
2215	FCGR3B	2.8419	0.039822
23047	PDS5B	-2.8419	0.039822
475	ATOX1	2.841	0.039861
23417	MLYCD	2.841	0.039861
4929	NR4A2	-2.84	0.039959
100129271	C1orf68	2.8396	0.039973
23164	MPRIP	2.8392	0.039984
9079	LDB2	-2.8388	0.039998
7297	TYK2	2.8382	0.040033
54541	DDIT4	2.8375	0.040088
332	BIRC5	2.8352	0.040186
51442	VGLL1	2.8355	0.040186
25829	TMEM184B	2.8348	0.040186
6901	TAZ	2.8354	0.040186
3104	ZBTB48	2.835	0.040186
7991	TUSC3	-2.8351	0.040186
8992	ATP6V0E1	-2.8351	0.040186
5048	PAFAH1B1	-2.8344	0.04019
1639	DCTN1	2.8314	0.040504

9957	HS3ST1	2.8316	0.040504
2316	FLNA	2.831	0.040517
5351	PLOD1	2.8296	0.040658
84223	IQCG	2.8278	0.040823
25789	TMEM59L	2.8278	0.040823
654	BMP6	2.8271	0.040852
55256	ADI1	-2.827	0.040852
9312	KCNB2	2.8257	0.04098
3416	IDE	-2.8254	0.040984
441263	)TX2P1-UPK3BP1-PMS2P1	2.8243	0.04109
26057	ANKRD17	-2.8211	0.04146
55048	VPS37C	2.8205	0.04151
55602	CDKN2AIP	-2.8199	0.041545
10150	MBNL2	-2.817	0.041884
8893	EIF2B5	2.8165	0.041912
8898	MTMR2	2.8156	0.041929
6881	TAF10	2.8157	0.041929
5817	PVR	2.8157	0.041929
6649	SOD3	2.8128	0.042213
3858	KRT10	2.8128	0.042213
4668	NAGA	2.8122	0.04226
3434	IFIT1	-2.8119	0.042261
1048	CEACAM5	2.8103	0.042434
3177	SLC29A2	-2.8097	0.042443
2824	GPM6B	-2.8098	0.042443
54828	BCAS3	2.8067	0.042801
23077	MYCBP2	-2.805	0.042989
11314	CD300A	2.8036	0.043139
57863	CADM3	-2.8033	0.04315
27094	KCNMB3	2.8027	0.04319
2664	GDI1	2.8014	0.04332
7270	TTF1	-2.7999	0.043486
1831	TSC22D3	2.7994	0.043516
2023	ENO1	2.7975	0.043741
22913	RALY	2.795	0.044012
29978	UBQLN2	-2.7949	0.044012
7528	YY1	-2.7931	0.044225
9644	SH3PXD2A	-2.7914	0.044418
7381	UQCRB	-2.7908	0.044464
5768	QSOX1	-2.7896	0.044546
4066	LYL1	2.7895	0.044546
51657	STYXL1	2.7894	0.044546
51025	PAM16	2.7888	0.044589
1047	CLGN	-2.7885	0.044592
8554	PIAS1	-2.7879	0.044637
29035	C16orf72	2.786	0.044861
10907	TXNL4A	2.784	0.045061
3954	LETM1	2.784	0.045061
1002	CDH4	2.7834	0.045073
22978	NT5C2	2.7834	0.045073
126298	IRGQ	2.78	0.04551
90	ACVR1	-2.7779	0.045727
79722	ANKRD55	2.7781	0.045727
2495	FTH1	2.7771	0.045739
11267	SNF8	2.777	0.045739
78994	PRR14	2.7772	0.045739
10512	SEMA3C	-2.7748	0.045986
3384	ICAM2	2.7747	0.045986
7134	TNNC1	2.7739	0.046057
5158	PDE6B	2.772	0.046241
55758	RCOR3	-2.7721	0.046241
23295	MGRN1	2.7718	0.046241
3045	HBD	2.7713	0.046278
55105	GPATCH2	2.7704	0.046355
4286	MITF	-2.7699	0.046392
3761	KCNJ4	2.7694	0.046434
2197	FAU	2.7684	0.046529

84525	HOPX	-2.7645	0.047051
7574	ZNF26	2.7632	0.047204
5799	PTPRN2	-2.7625	0.047265
54811	ZNF562	-2.7604	0.047534
9686	VGLL4	-2.7586	0.047638
1509	CTSD	2.759	0.047638
1351	COX8A	2.7586	0.047638
10451	VAV3	2.7586	0.047638
51449	PCYOX1	-2.7565	0.047878
868	CBLB	-2.7564	0.047878
1434	CSE1L	-2.7557	0.047939
131578	LRRC15	2.7552	0.047951
24	ABCA4	2.7551	0.047951
55884	WSB2	2.7531	0.048133
27303	RBMS3	-2.7528	0.048133
3646	EIF3E	-2.7533	0.048133
51428	DDX41	2.7528	0.048133
4591	TRIM37	2.7504	0.048414
784	CACNB3	2.7503	0.048414
10683	DLL3	2.7495	0.048453
2272	FHIT	2.7495	0.048453
79145	CHCHD7	2.7493	0.048453
23303	KIF13B	2.7482	0.048566
23301	EHBP1	-2.748	0.048566
79866	BORA	2.7455	0.04881
3661	IRF3	2.745	0.04881
10300	KATNB1	2.7458	0.04881
2013	EMP2	-2.7451	0.04881
6584	SLC22A5	-2.7451	0.04881
9545	RAB3D	2.7444	0.048859
79574	EPS8L3	2.7441	0.048866
2537	IFI6	2.7437	0.048883
4899	NRF1	-2.7422	0.048905
8648	NCOA1	-2.7425	0.048905
1488	CTBP2	-2.7429	0.048905
1452	CSNK1A1	-2.7425	0.048905
23400	ATP13A2	2.743	0.048905
3217	HOXB7	2.7406	0.049103
5604	MAP2K1	2.7399	0.049167
4999	ORC2	-2.7394	0.049209
6218	RPS17	2.7387	0.049266
6556	SLC11A1	2.7354	0.049734

Supplement File 1 Quality assessment of the microarray of AF downloaded from the Gene Expression Omnibus

arrayQualityMetrics report for raw_data GSE2240

- **Section 1: Between array comparison**
  - Distances between arrays
  - Principal Component Analysis
- **Section 2: Array intensity distributions**
  - Boxplots
  - Density plots
- **Section 3: Variance mean dependence**
  - Standard deviation versus rank of the mean
- **Section 4: Affymetrix specific plots**
  - Relative Log Expression (RLE)
  - Normalized Unscaled Standard Error (NUSE)
  - RNA digestion plot
  - Perfect matches and mismatches
- **Section 5: Individual array quality**
  - MA plots
  - Spatial distribution of M

- Array metadata and outlier detection overview

	array	sampleNames	*1	*2	*3	*4	*5	*6	Source.Name	Factor.Value..disease.	ScanDate
	1	GSM40990.CEL							GSE2240GSM40990	normal	08/28/02 11:06:45
	2	GSM40999.CEL	x	x					GSE2240GSM40999	normal	08/28/02 12:53:00
	3	GSM41000.CEL							GSE2240GSM41000	normal	08/28/02 13:03:51
	4	GSM41001.CEL							GSE2240GSM41001	normal	09/10/02 11:48:16
	5	GSM41002.CEL							GSE2240GSM41002	normal	08/28/02 14:29:16
	6	GSM41003.CEL							GSE2240GSM41003	normal	08/28/02 14:40:16
	7	GSM41004.CEL							GSE2240GSM41004	normal	08/28/02 14:51:19
	8	GSM41005.CEL							GSE2240GSM41005	normal	08/28/02 15:06:15
	9	GSM41006.CEL			x				GSE2240GSM41006	normal	09/10/02 11:24:43
	10	GSM41007.CEL							GSE2240GSM41007	normal	08/28/02 15:20:55
	11	GSM41008.CEL							GSE2240GSM41008	normal	08/28/02 15:48:29
	12	GSM40991.CEL							GSE2240GSM40991	normal	08/28/02 11:17:22
	13	GSM41009.CEL							GSE2240GSM41009	normal	08/28/02 13:14:29
	14	GSM40992.CEL							GSE2240GSM40992	normal	08/28/02 11:29:12
	15	GSM40993.CEL							GSE2240GSM40993	normal	08/28/02 11:42:02
	16	GSM40994.CEL							GSE2240GSM40994	normal	08/28/02 11:53:37
	17	GSM40995.CEL							GSE2240GSM40995	normal	08/28/02 12:07:29
	18	GSM40996.CEL							GSE2240GSM40996	normal	08/28/02 12:18:06
	19	GSM40997.CEL							GSE2240GSM40997	normal	08/28/02 12:31:15
	20	GSM40998.CEL							GSE2240GSM40998	normal	08/28/02 12:42:23
	21	GSM40980.CEL							GSE2240GSM40980	permanent atrial fibrillation	08/28/02 14:07:05
	22	GSM40989.CEL							GSE2240GSM40989	permanent atrial fibrillation	08/29/02 12:10:51
	23	GSM40981.CEL							GSE2240GSM40981	permanent atrial fibrillation	08/28/02 14:18:15
	24	GSM40982.CEL							GSE2240GSM40982	permanent atrial fibrillation	09/10/02 12:09:48
	25	GSM40983.CEL							GSE2240GSM40983	permanent atrial fibrillation	08/28/02 15:59:50
	26	GSM40984.CEL							GSE2240GSM40984	permanent atrial fibrillation	08/28/02 16:19:30
	27	GSM40985.CEL	x						GSE2240GSM40985	permanent atrial fibrillation	08/29/02 10:24:42
	28	GSM40986.CEL							GSE2240GSM40986	permanent atrial fibrillation	08/29/02 10:46:31
	29	GSM40987.CEL						x	GSE2240GSM40987	permanent atrial fibrillation	08/29/02 11:07:21
	30	GSM40988.CEL							GSE2240GSM40988	permanent atrial fibrillation	08/29/02 11:32:27

The columns named *1, *2, ... indicate the calls from the different outlier detection methods:

1. outlier detection by Distances between arrays
2. outlier detection by Boxplots
3. outlier detection by Relative Log Expression (RLE)
4. outlier detection by Normalized Unscaled Standard Error (NUSE)
5. outlier detection by MA plots
6. outlier detection by Spatial distribution of M

The outlier detection criteria are explained below in the respective sections. Arrays that were called outliers by at least one criterion are marked by checkbox selection in this table, and are indicated by highlighted lines or points in some of the plots below. By clicking the

checkboxes in the table, or on the corresponding points/lines in the plots, you can modify the selection. To reset the selection, reload the HTML page in your browser.

At the scope covered by this software, outlier detection is a poorly defined question, and there is no 'right' or 'wrong' answer. These are hints which are intended to be followed up manually. If you want to automate outlier detection, you need to limit the scope to a particular platform and experimental design, and then choose and calibrate the metrics used.

Section 1: Between array comparison

- Figure 1: Distances between arrays.

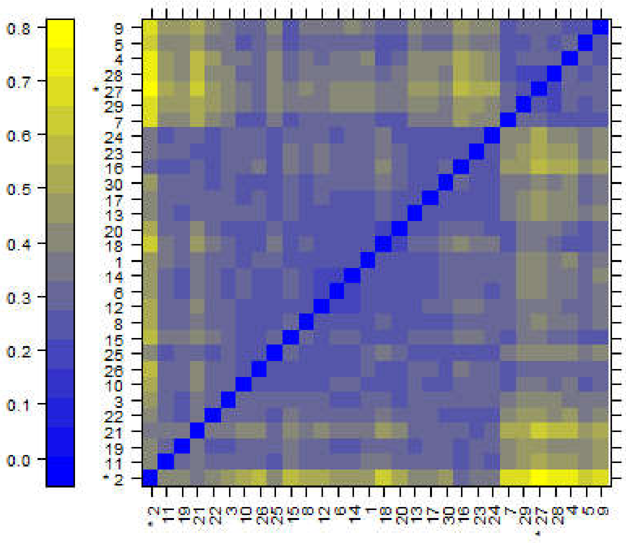

Figure 1 (PDF file) shows a false color heatmap of the distances between arrays. The color scale is chosen to cover the range of distances encountered in the dataset. Patterns in this plot can indicate clustering of the arrays either because of intended biological or unintended experimental factors (batch effects). The distance d_{ab} between two arrays a and b is computed as the mean absolute difference (L_1-distance) between the data of the arrays (using the data from all probes without filtering). In formula, d_{ab} = mean | M_{ai} - M_{bi} |, where M_{ai} is the value of the i-th probe on the a-th array. Outlier detection was performed by looking for arrays for which the sum of the distances to all other arrays, $S_a = \Sigma_b d_{ab}$ was exceptionally large. 2 such arrays were detected, and they are marked by an asterisk, *.

- Figure 2: Outlier detection for Distances between arrays.

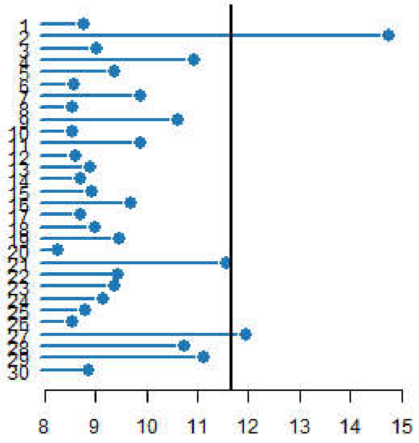

Figure 2 (PDF file) shows a bar chart of the sum of distances to other arrays S_a, the outlier detection criterion from the previous figure. The bars are shown in the original order of the arrays. Based on the distribution of the values across all arrays, a threshold of 11.7 was determined, which is indicated by the vertical line. 2 arrays exceeded the threshold and were considered outliers.

- Figure 3: Principal Component Analysis.

array
sampleNames
Source.Name
Factor.Value..disease.
ScanDate

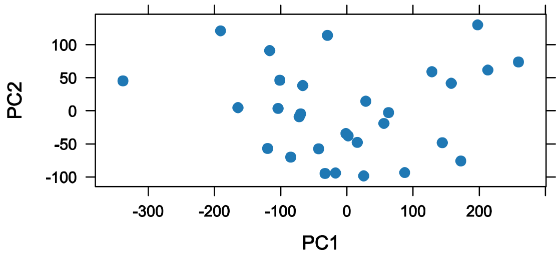

Figure 3 (PDF file) shows a scatterplot of the arrays along the first two principal components. You can use this plot to explore if the arrays cluster, and whether this is according to an intended experimental factor (you can indicate such a factor by color using the 'intgroup' argument), or according to unintended causes such as batch effects. Move the mouse over the points to see the sample names.
Principal component analysis is a dimension reduction and visualisation technique that is here used to project the multivariate data vector of each array into a two-dimensional plot, such that the spatial arrangement of the points in the plot reflects the overall data (dis)similarity between the arrays.

Section 2: Array intensity distributions

- Figure 4: Boxplots.

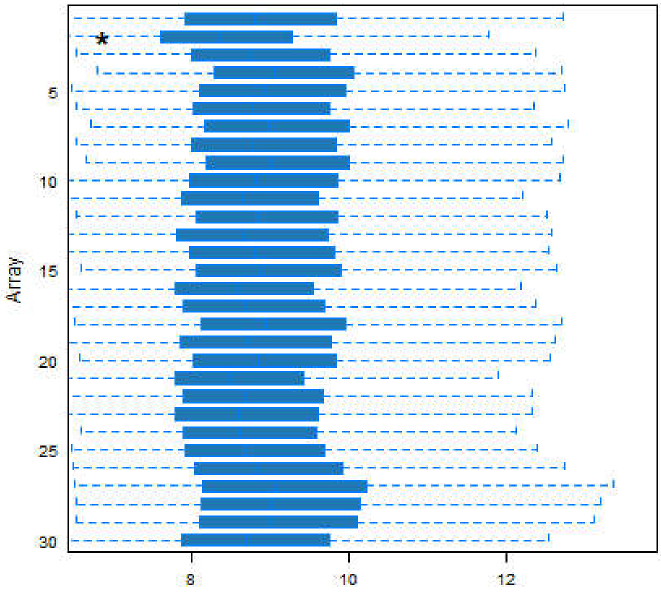

Figure 4 (PDF file) shows boxplots representing summaries of the signal intensity distributions of the arrays. Each box corresponds to one array. Typically, one expects the boxes to have similar positions and widths. If the distribution of an array is very different from the others, this may indicate an experimental problem. Outlier detection was performed by computing the Kolmogorov-Smirnov statistic K_a between each array's distribution and the distribution of the pooled data.

- Figure 5: Outlier detection for Boxplots.

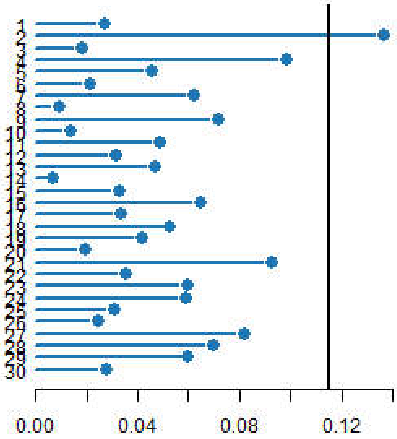

Figure 5 [(PDF file)] shows a bar chart of the Kolmogorov-Smirnov statistic K_a, the outlier detection criterion from the previous figure. The bars are shown in the original order of the arrays. Based on the distribution of the values across all arrays, a threshold of 0.115 was determined, which is indicated by the vertical line. One array exceeded the threshold and was considered an outlier.

- Figure 6: Density plots.

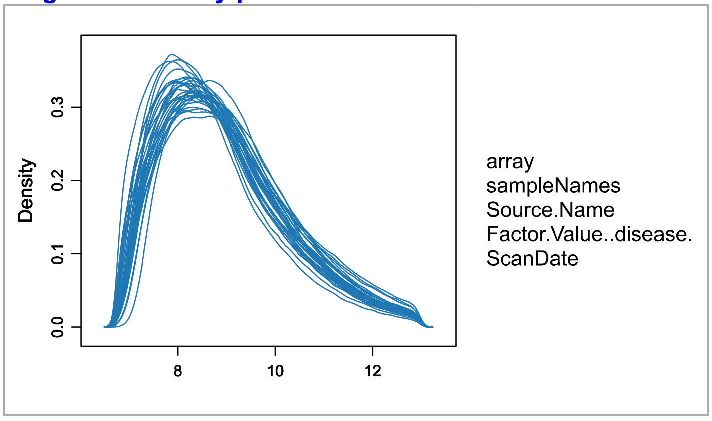

Figure 6 [(PDF file)] shows density estimates (smoothed histograms) of the data. Typically, the distributions of the arrays should have similar shapes and ranges. Arrays whose distributions are very different from the others should be considered for possible problems. Various features of the distributions can be indicative of quality related phenomena. For instance, high levels of background will shift an array's distribution to the right. Lack of signal diminishes its right right tail. A bulge at the upper end of the intensity range often indicates signal saturation.

Section 3: Variance mean dependence

- Figure 7: Standard deviation versus rank of the mean.

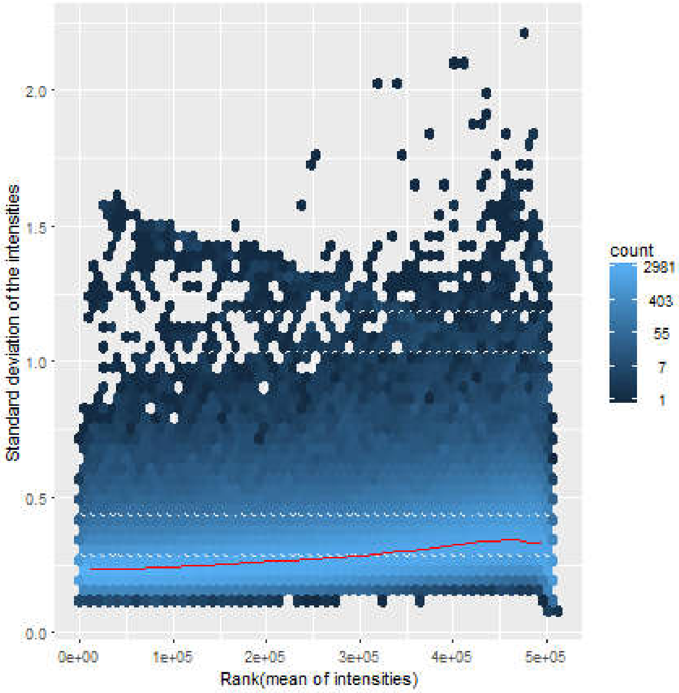

Figure 7 [(PDF file)] shows a density plot of the standard deviation of the intensities across arrays on the *y*-axis versus the rank of their mean

on the *x*-axis. The red dots, connected by lines, show the running median of the standard deviation. After normalisation and transformation to a logarithm(-like) scale, one typically expects the red line to be approximately horizontal, that is, show no substantial trend. In some cases, a hump on the right hand of the x-axis can be observed and is symptomatic of a saturation of the intensities.

Section 4: Affymetrix specific plots

- Figure 8: Relative Log Expression (RLE).

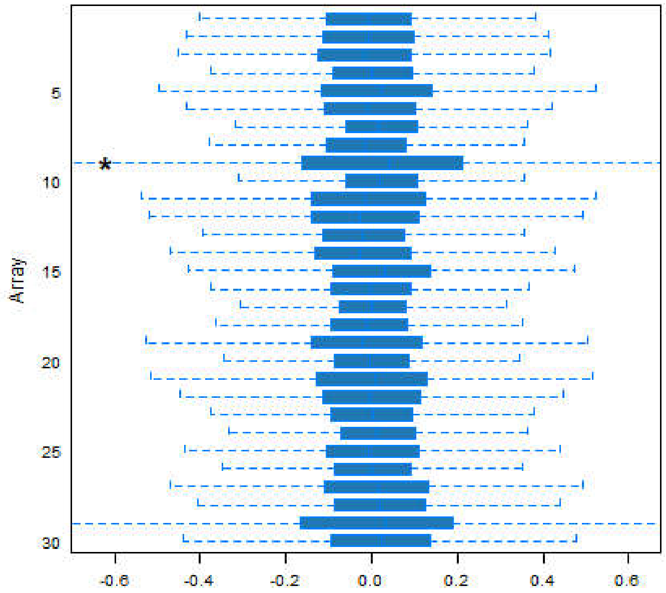

Figure 8 (PDF file) shows the *Relative Log Expression (RLE)* plot. Arrays whose boxes are centered away from 0 and/or are more spread out are potentially problematic. Outlier detection was performed by computing the Kolmogorov-Smirnov statistic R_a between each array's RLE values and the pooled, overall distribution of RLE values.

- Figure 9: Outlier detection for Relative Log Expression (RLE).

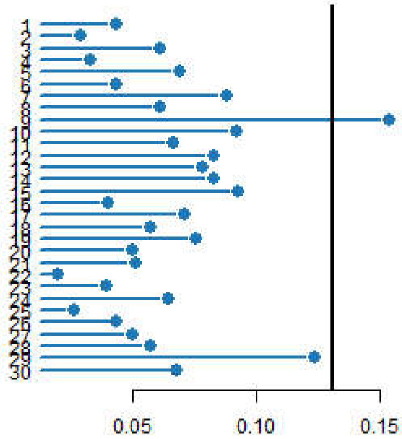

Figure 9 (PDF file) shows a bar chart of the Kolmogorov-Smirnov statistic R_a of the RLE values, the outlier detection criterion from the previous figure. The bars are shown in the original order of the arrays. Based on the distribution of the values across all arrays, a threshold of 0.131 was determined, which is indicated by the vertical line. One array exceeded the threshold and was considered an outlier.

- Figure 10: Normalized Unscaled Standard Error (NUSE).

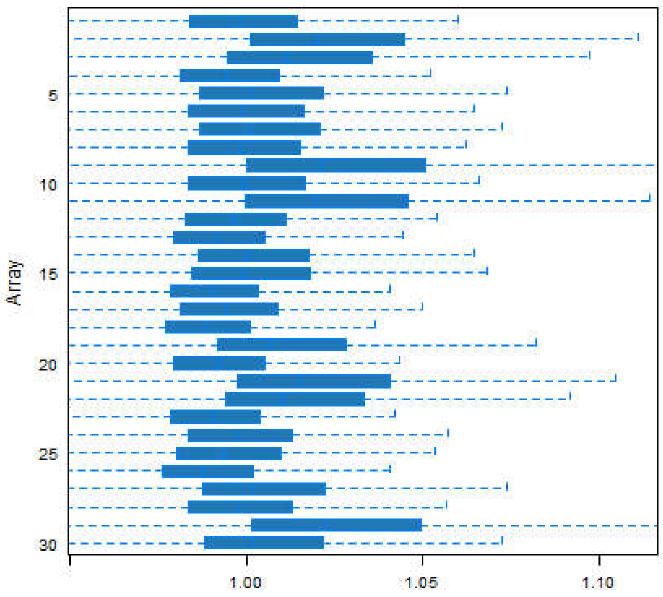

Figure 10 [(PDF file)](#) shows the *Normalized Unscaled Standard Error (NUSE)* plot. For each array, the boxes should be centered around 1. An array were the values are elevated relative to the other arrays is typically of lower quality. Outlier detection was performed by computing the 75% quantile N_a of each array's NUSE values and looking for arrays with large N_a.

- Figure 11: Outlier detection for Normalized Unscaled Standard Error (NUSE).

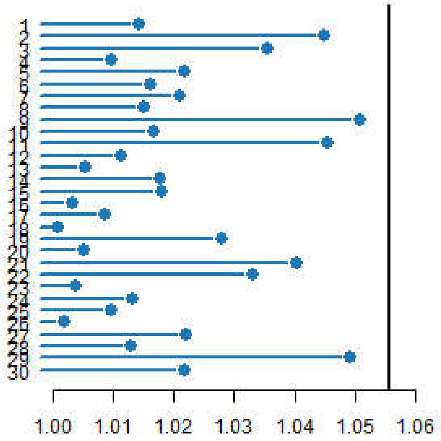

Figure 11 [(PDF file)](#) shows a bar chart of the N_a, the outlier detection criterion from the previous figure. The bars are shown in the original order of the arrays. Based on the distribution of the values across all arrays, a threshold of 1.06 was determined, which is indicated by the vertical line. None of the arrays exceeded the threshold and was considered an outlier.

- Figure 12: RNA digestion plot.

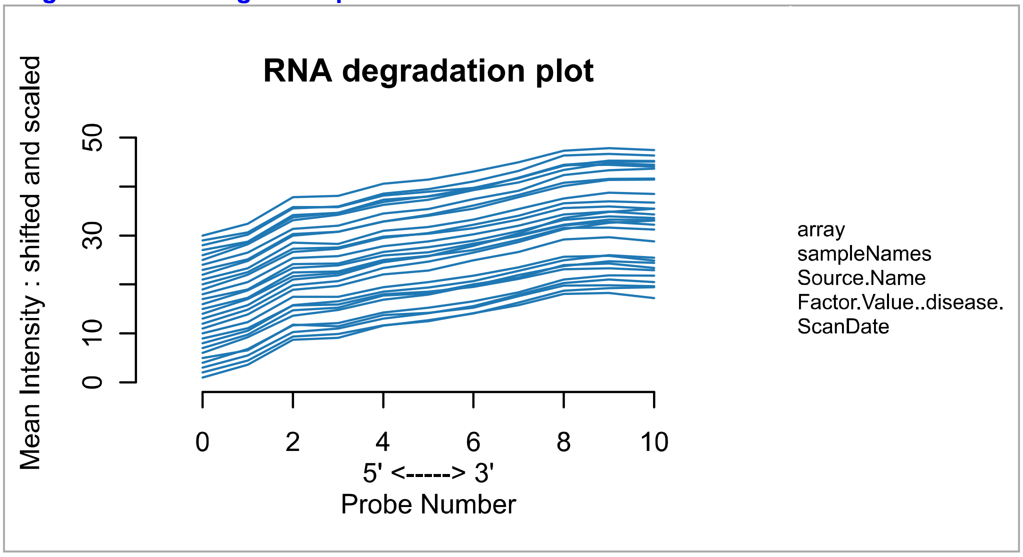

Figure 12 [(PDF file)](#) shows the *RNA digestion* plot. The shown values are computed from the preprocessed data (after background correction and quantile normalisation). Each array is represented by a single line; move the mouse over the lines to see their corresponding sample names. The plot can be used to identify array(s) that have a slope very different from the others. This could indicate that the RNA used for that array has been handled differently from what was done for the other arrays.

- Figure 13: Perfect matches and mismatches.

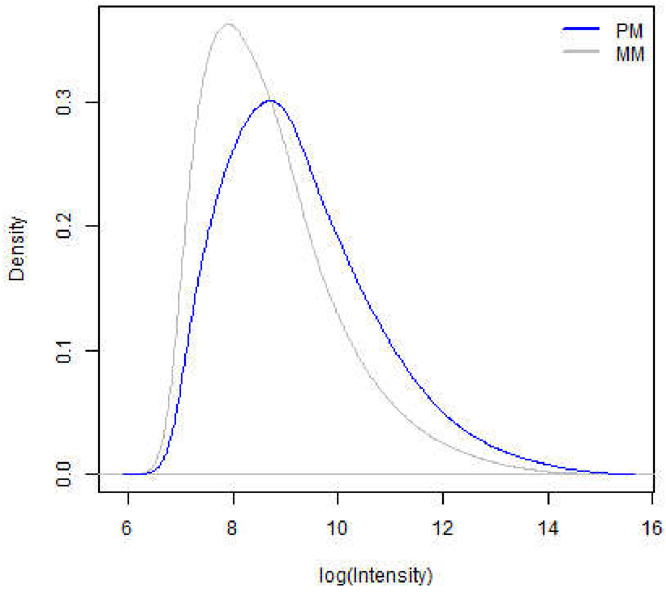

Figure shows the density distributions of the $\log_2$ intensities grouped by the matching type of the probes. The blue line shows a density estimate (smoothed histogram) from intensities of perfect match probes (PM), the grey line, one from the mismatch probes (MM). We expect that MM probes have poorer hybridization than PM probes, and thus that the PM curve be to the right of the MM curve.

Section 5: Individual array quality

- Figure 14: MA plots.

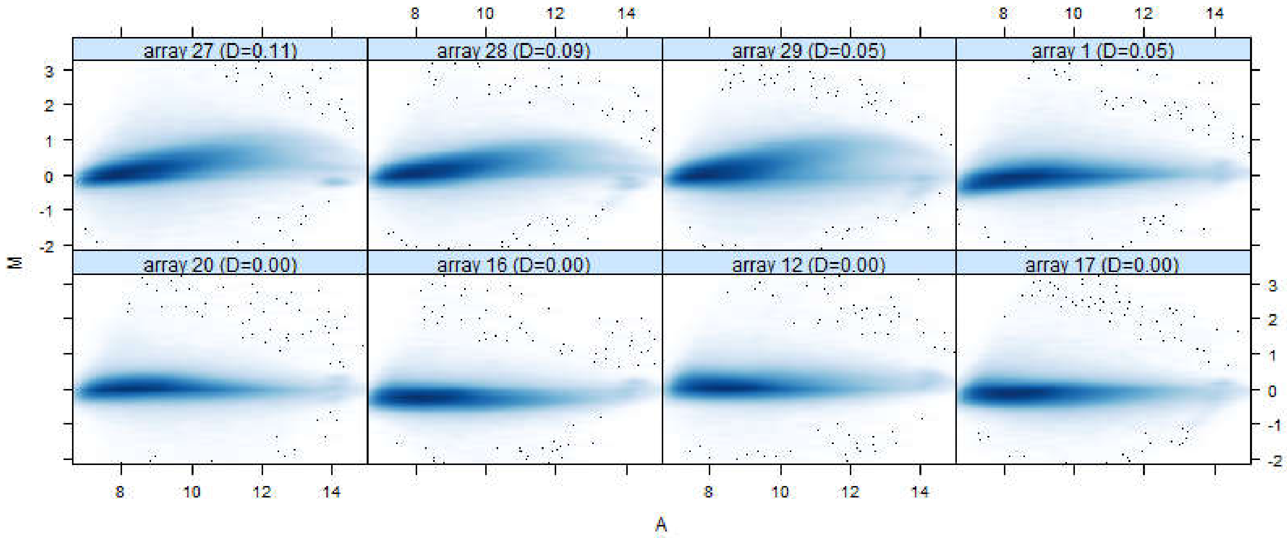

Figure 14 (PDF file) shows MA plots. M and A are defined as:
$M = \log_2(I_1) - \log_2(I_2)$
$A = 1/2 (\log_2(I_1)+\log_2(I_2))$,
where I_1 is the intensity of the array studied,and I_2 is the intensity of a "pseudo"-array that consists of the median across arrays. Typically, we expect the mass of the distribution in an MA plot to be concentrated along the M = 0 axis, and there should be no trend in M as a function of A. If there is a trend in the lower range of A, this often indicates that the arrays have different background intensities; this may be addressed by background correction. A trend in the upper range of A can indicate saturation of the measurements; in mild cases, this may be addressed by non-linear normalisation (e.g. quantile normalisation).
Outlier detection was performed by computing Hoeffding's statistic D_a on the joint distribution of A and M for each array. Shown are first the 4 arrays with the highest values of D_a, then the 4 arrays with the lowest values. The value of D_a is shown in the panel headings. 0 arrays had D_a>0.15 and were marked as outliers. For more information on Hoeffing's *D*-statistic, please see the manual page of the function `hoeffd` in the `Hmisc` package.

- Figure 15: Outlier detection for MA plots.

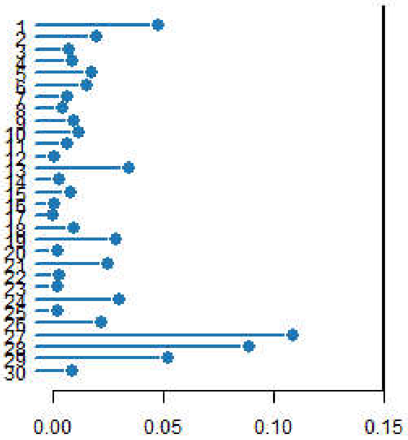

Figure 15 [(PDF file)](#) shows a bar chart of the D_a, the outlier detection criterion from the previous figure. The bars are shown in the original order of the arrays. A threshold of 0.15 was used, which is indicated by the vertical line. None of the arrays exceeded the threshold and was considered an outlier.

- Figure 16: Spatial distribution of M.

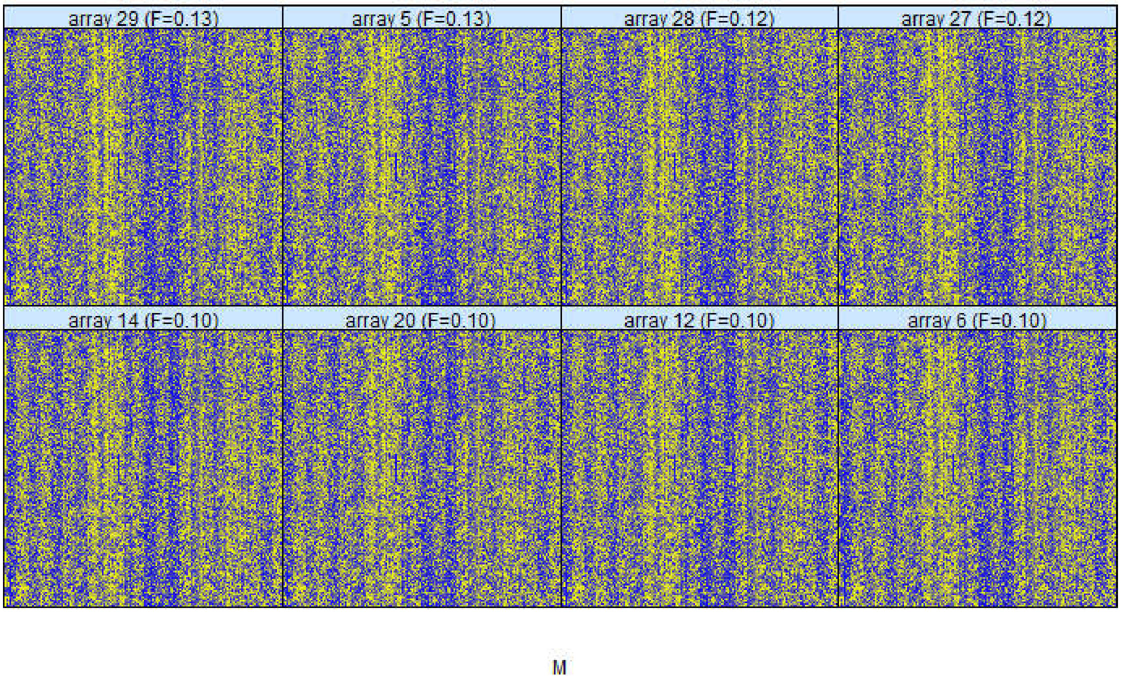

Figure 16 [(PDF file)](#) shows false color representations of the arrays' spatial distributions of feature intensities (M). Normally, when the features are distributed randomly on the arrays, one expects to see a uniform distribution; control features with particularly high or low intensities may stand out. The color scale is proportional to the ranks of the probe intensities . Note that the rank scale has the potential to amplify patterns that are small in amplitudebut systematic within an array. It is possible to switch off the rank scaling by modifying the argument`scale` in the call of the `aqm.spatial` function.
Outlier detection was performed by computing F_a , the sum of the absolutes value of low frequency Fourier coefficients, as a measure of large scale spatial structures. Shown are first the 4 arrays with the highest values of F_a, then the 4 arrays with the lowest values. The value of F_a is shown in the panel headings.

- Figure 17: Outlier detection for Spatial distribution of M.

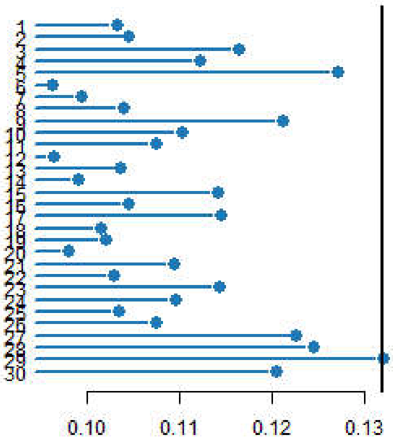

Figure 17 (PDF file) shows a bar chart of the F_a, the outlier detection criterion from the previous figure. The bars are shown in the original order of the arrays. Based on the distribution of the values across all arrays, a threshold of 0.132 was determined, which is indicated by the vertical line. One array exceeded the threshold and was considered an outlier.

This report has been created with arrayQualityMetrics 3.34.0 under R version 3.4.1 (2017-06-30).

(Page generated on Thu Apr 26 17:25:06 2018 by hwriter)

arrayQualityMetrics report for raw_data GSE14975

- **Section 1: Between array comparison**
  - Distances between arrays
  - Principal Component Analysis
- **Section 2: Array intensity distributions**
  - Boxplots
  - Density plots
- **Section 3: Variance mean dependence**
  - Standard deviation versus rank of the mean
- **Section 4: Affymetrix specific plots**
  - Relative Log Expression (RLE)
  - Normalized Unscaled Standard Error (NUSE)
  - RNA digestion plot
  - Perfect matches and mismatches
- **Section 5: Individual array quality**
  - MA plots
  - Spatial distribution of M

- Array metadata and outlier detection overview

	array	sampleNames	*1	*2	*3	*4	*5	*6	Source.Name	Disease	ScanDate
	1	GSM373963.CEL							GSM373963 1	control	06/22/06 11:27:59
	2	GSM373965.CEL							GSM373965 1	control	06/16/06 11:58:50
	3	GSM373964.CEL							GSM373964 1	control	06/16/06 12:57:01
	4	GSM373962.CEL							GSM373962 1	control	06/22/06 11:14:51
	5	GSM373961.CEL							GSM373961 1	control	06/16/06 11:31:32
	6	GSM373959.CEL							GSM373959 1	atrial fibrillation patient	06/16/06 11:44:47
	7	GSM373957.CEL							GSM373957 1	atrial fibrillation patient	06/16/06 11:17:30
	8	GSM373956.CEL							GSM373956 1	atrial fibrillation patient	06/22/06 10:46:02
	9	GSM373960.CEL	x	x		x			GSM373960 1	atrial fibrillation patient	06/22/06 11:00:23
	10	GSM373958.CEL							GSM373958 1	atrial fibrillation patient	06/16/06 12:44:01

The columns named *1, *2, ... indicate the calls from the different outlier detection methods:

1. outlier detection by Distances between arrays
2. outlier detection by Boxplots
3. outlier detection by Relative Log Expression (RLE)
4. outlier detection by Normalized Unscaled Standard Error (NUSE)
5. outlier detection by MA plots
6. outlier detection by Spatial distribution of M

The outlier detection criteria are explained below in the respective sections. Arrays that were called outliers by at least one criterion are marked by checkbox selection in this table, and are indicated by highlighted lines or points in some of the plots below. By clicking the checkboxes in the table, or on the corresponding points/lines in the plots, you can modify the selection. To reset the selection, reload the HTML page in your browser.

At the scope covered by this software, outlier detection is a poorly defined question, and there is no 'right' or 'wrong' answer. These are hints which are intended to be followed up manually. If you want to automate outlier detection, you need to limit the scope to a particular platform and experimental design, and then choose and calibrate the metrics used.

Section 1: Between array comparison

- Figure 1: Distances between arrays.

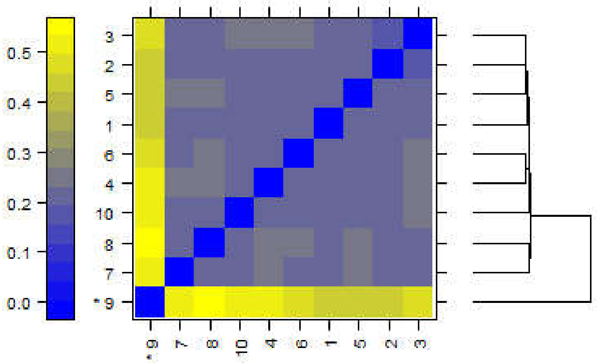

Figure 1 [(PDF file)](#) shows a false color heatmap of the distances between arrays. The color scale is chosen to cover the range of distances encountered in the dataset. Patterns in this plot can indicate clustering of the arrays either because of intended biological or unintended experimental factors (batch effects). The distance d_{ab} between two arrays a and b is computed as the mean absolute difference (L_1-distance) between the data of the arrays (using the data from all probes without filtering). In formula, d_{ab} = mean | M_{ai} - M_{bi} |, where M_{ai} is the value of the i-th probe on the a-th array. Outlier detection was performed by looking for arrays for which the sum of the distances to all other arrays, $S_a = \Sigma_b d_{ab}$ was exceptionally large. One such array was detected, and it is marked by an asterisk, *.

- Figure 2: Outlier detection for Distances between arrays.

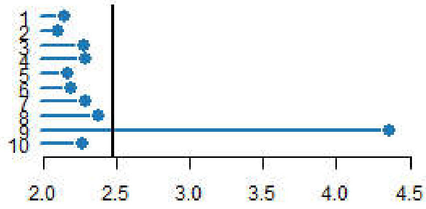

Figure 2 [(PDF file)](#) shows a bar chart of the sum of distances to other arrays S_a, the outlier detection criterion from the previous figure. The bars are shown in the original order of the arrays. Based on the distribution of the values across all arrays, a threshold of 2.47 was determined, which is indicated by the vertical line. One array exceeded the threshold and was considered an outlier.

- Figure 3: Principal Component Analysis.

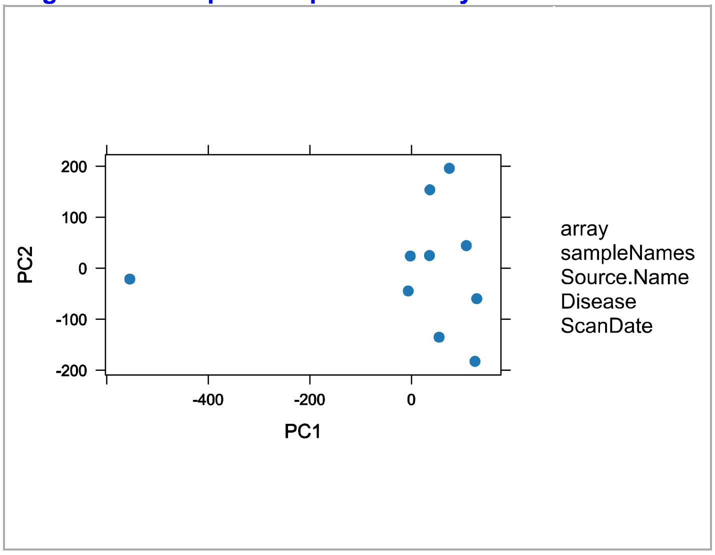

Figure 3 [(PDF file)](#) shows a scatterplot of the arrays along the first two principal components. You can use this plot to explore if the arrays cluster, and whether this is according to an intended experimental factor (you can indicate such a factor by color using the 'intgroup' argument), or according to unintended causes such as batch effects. Move the mouse over the points to see the sample names.
Principal component analysis is a dimension reduction and visualisation technique that is here used to project the multivariate data vector of each array into a two-dimensional plot, such that the spatial arrangement of the points in the plot reflects the overall data (dis)similarity between the arrays.

Section 2: Array intensity distributions

- Figure 4: Boxplots.

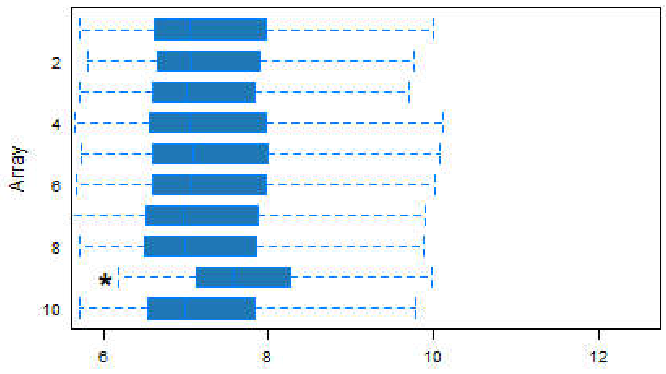

Figure 4 [(PDF file)](#) shows boxplots representing summaries of the signal intensity distributions of the arrays. Each box corresponds to one array. Typically, one expects the boxes to have similar positions and widths. If the distribution of an array is very different from the others, this may indicate an experimental problem. Outlier detection was performed by computing the Kolmogorov-Smirnov statistic K_a between each array's distribution and the distribution of the pooled data.

- Figure 5: Outlier detection for Boxplots.

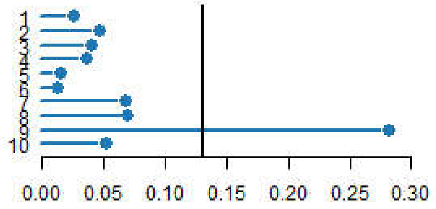

Figure 5 [(PDF file)](#) shows a bar chart of the Kolmogorov-Smirnov statistic K_a, the outlier detection criterion from the previous figure. The bars are shown in the original order of the arrays. Based on the distribution of the values across all arrays, a threshold of 0.13 was determined, which is indicated by the vertical line. One array exceeded the threshold and was considered an outlier.

- Figure 6: Density plots.

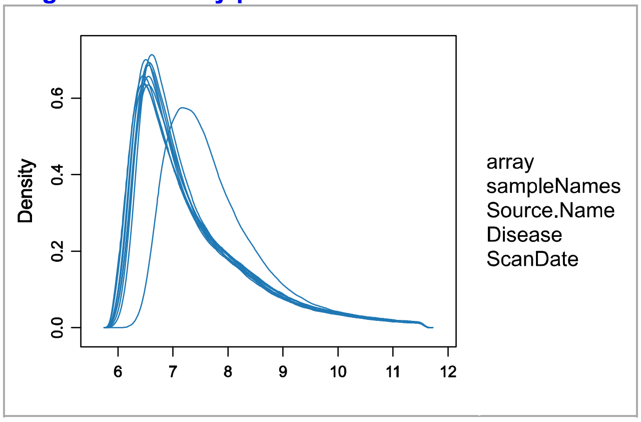

Figure 6 [(PDF file)](#) shows density estimates (smoothed histograms) of the data. Typically, the distributions of the arrays should have similar shapes and ranges. Arrays whose distributions are very different from the others should be considered for possible problems. Various features of the distributions can be indicative of quality related phenomena. For instance, high levels of background will shift an array's distribution to the right. Lack of signal diminishes its right right tail. A bulge at the upper end of the intensity range often indicates signal saturation.

Section 3: Variance mean dependence

- Figure 7: Standard deviation versus rank of the mean.

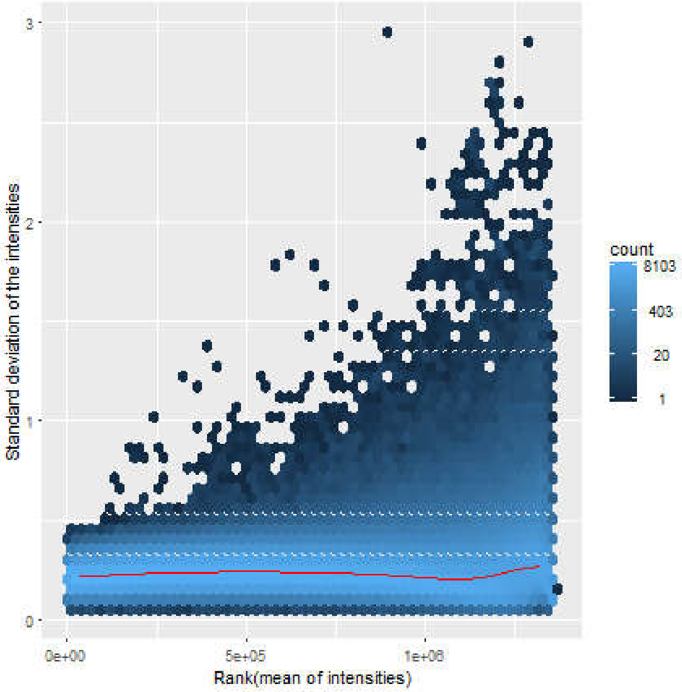

Figure 7 (PDF file) shows a density plot of the standard deviation of the intensities across arrays on the *y*-axis versus the rank of their mean on the *x*-axis. The red dots, connected by lines, show the running median of the standard deviation. After normalisation and transformation to a logarithm(-like) scale, one typically expects the red line to be approximately horizontal, that is, show no substantial trend. In some cases, a hump on the right hand of the x-axis can be observed and is symptomatic of a saturation of the intensities.

Section 4: Affymetrix specific plots

- Figure 8: Relative Log Expression (RLE).

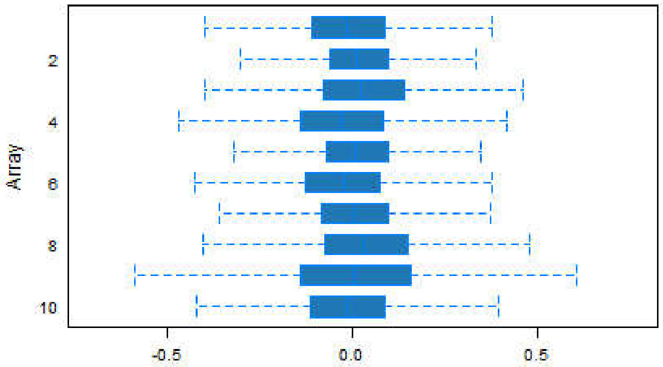

Figure 8 (PDF file) shows the *Relative Log Expression (RLE)* plot. Arrays whose boxes are centered away from 0 and/or are more spread out are potentially problematic. Outlier detection was performed by computing the Kolmogorov-Smirnov statistic R_a between each array's RLE values and the pooled, overall distribution of RLE values.

- Figure 9: Outlier detection for Relative Log Expression (RLE).

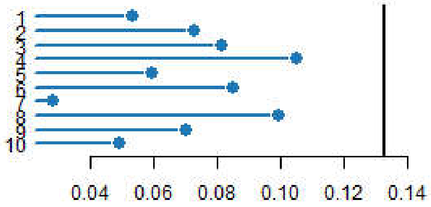

Figure 9 (PDF file) shows a bar chart of the Kolmogorov-Smirnov statistic R_a of the RLE values, the outlier detection criterion from the previous figure. The bars are shown in the original order of the arrays. Based on the distribution of the values across all arrays, a threshold of 0.133 was determined, which is indicated by the vertical line. None of the arrays exceeded the threshold and was considered an outlier.

- Figure 10: Normalized Unscaled Standard Error (NUSE).

arrayQualityMetrics report for raw_data GSE41177

- **Section 1: Between array comparison**
  - Distances between arrays
  - Principal Component Analysis
- **Section 2: Array intensity distributions**
  - Boxplots
  - Density plots
- **Section 3: Variance mean dependence**
  - Standard deviation versus rank of the mean
- **Section 4: Affymetrix specific plots**
  - Relative Log Expression (RLE)
  - Normalized Unscaled Standard Error (NUSE)
  - RNA digestion plot
  - Perfect matches and mismatches
- **Section 5: Individual array quality**
  - MA plots
  - Spatial distribution of M

- Array metadata and outlier detection overview

	array	sampleNames	*1	*2	*3	*4	*5	*6	Source.Name	Disease	ScanDate
☐	1	GSM1006253_20110223_plus2_0354_YYH_51_L__HG_U133_Plus_2_.CEL							GSM1006253 1	Atrial fibrillation	2011-02-23T03:38:21Z
☐	2	GSM1006251_20090828_U133_plus_2_0040_YYH__HG_U133_Plus_2_.CEL							GSM1006251 1	Atrial fibrillation	2009-08-28T04:42:49Z
☐	3	GSM1006249_20090910_U133_plus_2_0047_YYH__HG_U133_Plus_2_.CEL	x	x	x	x	x		GSM1006249 1	Atrial fibrillation	2009-09-10T04:16:42Z
☐	4	GSM1006247_20090910_U133_plus_2_0046_YYH__HG_U133_Plus_2_.CEL	x	x		x			GSM1006247 1	Atrial fibrillation	2009-09-10T05:08:18Z
☐	5	GSM1006245_20110223_plus2_0359_YYH_69_L__HG_U133_Plus_2_.CEL							GSM1006245 1	Atrial fibrillation	2011-02-23T04:32:38Z
☐	6	GSM1005444_20110315_plus2_0366_YYH_53_L__HG_U133_Plus_2_.CEL			x				GSM1005444 1	Atrial fibrillation	2011-03-15T03:59:40Z
☐	7	GSM1005442_20110315_plus2_0364_YYH_46_L__HG_U133_Plus_2_.CEL							GSM1005442 1	Atrial fibrillation	2011-03-15T03:37:57Z
☐	8	GSM1005440_20110315_plus2_0362_YYH_64_L__HG_U133_Plus_2_.CEL							GSM1005440 1	Atrial fibrillation	2011-03-15T03:16:09Z
☐	9	GSM1005438_20110324_plus2_0376_YYH_26_L__HG_U133_Plus_2_.CEL							GSM1005438 1	Atrial fibrillation	2011-03-24T04:18:25Z
☐	10	GSM1005436_20110324_plus2_0374_YYH_25_L__HG_U133_Plus_2_.CEL							GSM1005436 1	Atrial fibrillation	2011-03-24T03:56:20Z
☐	11	GSM1005434_20110324_plus2_0372_YYH_24_L__HG_U133_Plus_2_.CEL							GSM1005434 1	Atrial fibrillation	2011-03-24T03:34:27Z
☐	12	GSM1005432_20110324_plus2_0370_YYH_19_L__HG_U133_Plus_2_.CEL							GSM1005432 1	Atrial fibrillation	2011-03-24T03:12:41Z
☐	13	GSM1005430_20110414_plus2_0384_YYH_27L__HG_U133_Plus_2_.CEL							GSM1005430 1	Atrial fibrillation	2011-04-14T03:58:01Z
☐	14	GSM1005428_20110414_plus2_0382_YYH_17L__HG_U133_Plus_2_.CEL							GSM1005428 1	Atrial fibrillation	2011-04-14T03:36:17Z
☐	15	GSM1005426_20110414_plus2_0380_YYH_54L__HG_U133_Plus_2_.CEL							GSM1005426 1	Atrial fibrillation	2011-04-14T03:14:36Z
☐	16	GSM1005424_20110506_plus2_0400_YYH_36L__HG_U133_Plus_2_.CEL							GSM1005424 1	Atrial fibrillation	2011-05-06T03:21:42Z
☐	17	GSM1005422_20120801_plus2_0612_YYH_81L__HG_U133_Plus_2_.CEL							GSM1005422 1	Sinus rhythm	2012-08-01T03:38:42Z
☐	18	GSM1005420_20120815_plus2_0624_YYH_78L__HG_U133_Plus_2_.CEL	x		x	x			GSM1005420 1	Sinus rhythm	2012-08-15T03:59:42Z
☐	19	GSM1005418_20120815_plus2_0622_YYH_76L__HG_U133_Plus_2_.CEL					x		GSM1005418 1	Sinus rhythm	2012-08-15T04:37:35Z

The columns named *1, *2, ... indicate the calls from the different outlier detection methods:

1. outlier detection by Distances between arrays
2. outlier detection by Boxplots
3. outlier detection by Relative Log Expression (RLE)
4. outlier detection by Normalized Unscaled Standard Error (NUSE)
5. outlier detection by MA plots
6. outlier detection by Spatial distribution of M

The outlier detection criteria are explained below in the respective sections. Arrays that were called outliers by at least one criterion are marked by checkbox selection in this table, and are indicated by highlighted lines or points in some of the plots below. By clicking the checkboxes in the table, or on the corresponding points/lines in the plots, you can modify the selection. To reset the selection, reload the

HTML page in your browser.

At the scope covered by this software, outlier detection is a poorly defined question, and there is no 'right' or 'wrong' answer. These are hints which are intended to be followed up manually. If you want to automate outlier detection, you need to limit the scope to a particular platform and experimental design, and then choose and calibrate the metrics used.

Section 1: Between array comparison

- Figure 1: Distances between arrays.

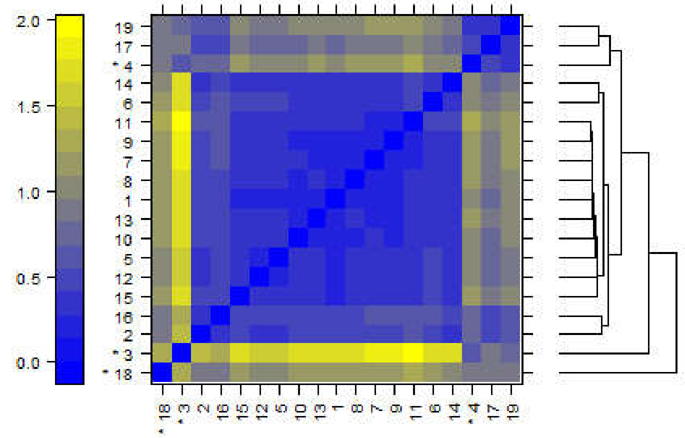

Figure 1 (PDF file) shows a false color heatmap of the distances between arrays. The color scale is chosen to cover the range of distances encountered in the dataset. Patterns in this plot can indicate clustering of the arrays either because of intended biological or unintended experimental factors (batch effects). The distance d_{ab} between two arrays *a* and *b* is computed as the mean absolute difference (L_1-distance) between the data of the arrays (using the data from all probes without filtering). In formula, d_{ab} = mean | M_{ai} - M_{bi} |, where M_{ai} is the value of the *i*-th probe on the *a*-th array. Outlier detection was performed by looking for arrays for which the sum of the distances to all other arrays, $S_a = \Sigma_b\, d_{ab}$ was exceptionally large. 3 such arrays were detected, and they are marked by an asterisk, *.

- Figure 2: Outlier detection for Distances between arrays.

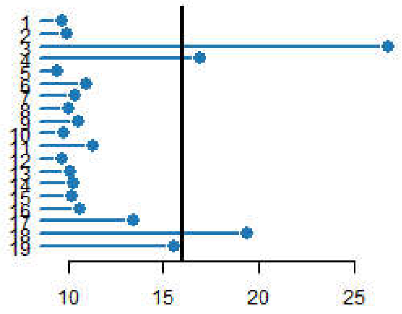

Figure 2 (PDF file) shows a bar chart of the sum of distances to other arrays S_a, the outlier detection criterion from the previous figure. The bars are shown in the original order of the arrays. Based on the distribution of the values across all arrays, a threshold of 16 was determined, which is indicated by the vertical line. 3 arrays exceeded the threshold and were considered outliers.

- Figure 3: Principal Component Analysis.

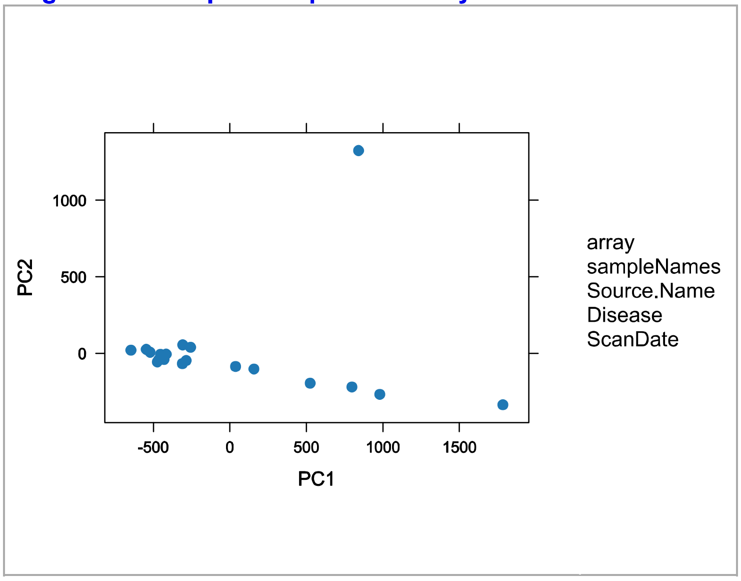

Figure 3 (PDF file) shows a scatterplot of the arrays along the first two principal components. You can use this plot to explore if the arrays cluster, and whether this is according to an intended experimental factor (you can indicate such a factor by color using the 'intgroup' argument), or according to unintended causes such as batch effects. Move the mouse over the points to see the sample names.
Principal component analysis is a dimension reduction and visualisation technique that is here used to project the multivariate data vector of each array into a two-dimensional plot, such that the spatial arrangement of the points in the plot reflects the overall data (dis)similarity between the arrays.

Section 2: Array intensity distributions

- Figure 4: Boxplots.

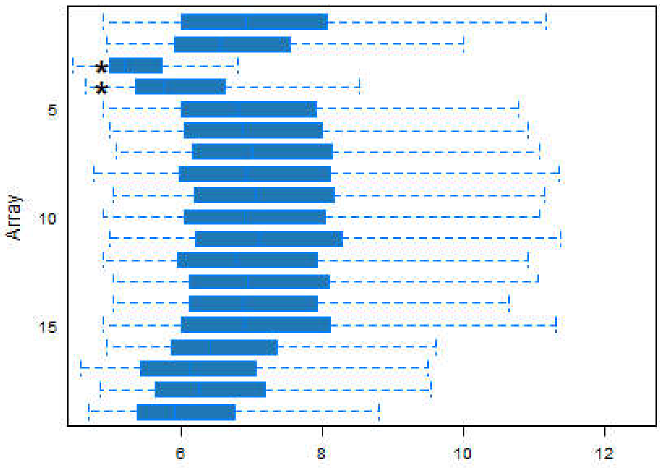

Figure 4 (PDF file) shows boxplots representing summaries of the signal intensity distributions of the arrays. Each box corresponds to one array. Typically, one expects the boxes to have similar positions and widths. If the distribution of an array is very different from the others, this may indicate an experimental problem. Outlier detection was performed by computing the Kolmogorov-Smirnov statistic K_a between each array's distribution and the distribution of the pooled data.

- Figure 5: Outlier detection for Boxplots.

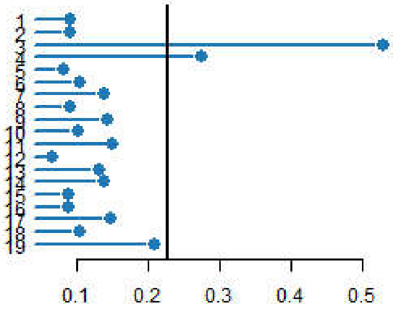

Figure 5 (PDF file) shows a bar chart of the Kolmogorov-Smirnov statistic K_a, the outlier detection criterion from the previous figure. The bars are shown in the original order of the arrays. Based on the distribution of the values across all arrays, a threshold of 0.227 was determined, which is indicated by the vertical line. 2 arrays exceeded the threshold and were considered outliers.

- Figure 6: Density plots.

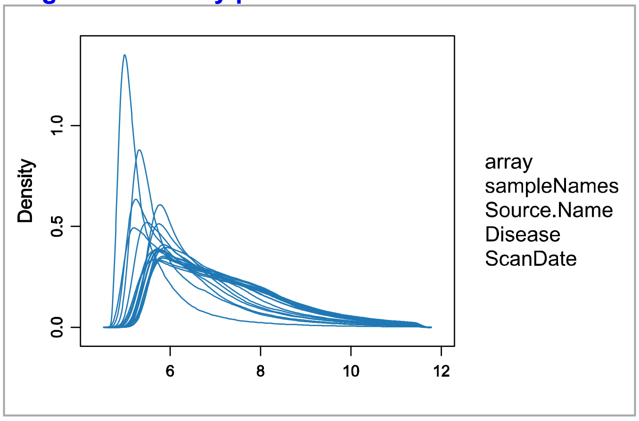

Figure 6 (PDF file) shows density estimates (smoothed histograms) of the data. Typically, the distributions of the arrays should have similar shapes and ranges. Arrays whose distributions are very different from the others should be considered for possible problems. Various features of the distributions can be indicative of quality related phenomena. For instance, high levels of background will shift an array's distribution to the right. Lack of signal diminishes its right right tail. A bulge at the upper end of the intensity range often indicates signal

saturation.

Section 3: Variance mean dependence

- Figure 7: Standard deviation versus rank of the mean.

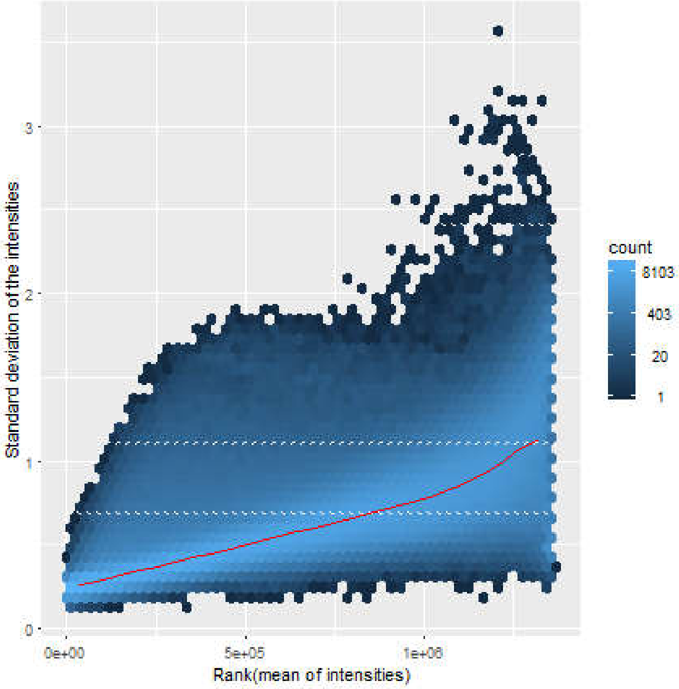

Figure 7 (PDF file) shows a density plot of the standard deviation of the intensities across arrays on the *y*-axis versus the rank of their mean on the *x*-axis. The red dots, connected by lines, show the running median of the standard deviation. After normalisation and transformation to a logarithm(-like) scale, one typically expects the red line to be approximately horizontal, that is, show no substantial trend. In some cases, a hump on the right hand of the x-axis can be observed and is symptomatic of a saturation of the intensities.

Section 4: Affymetrix specific plots

- Figure 8: Relative Log Expression (RLE).

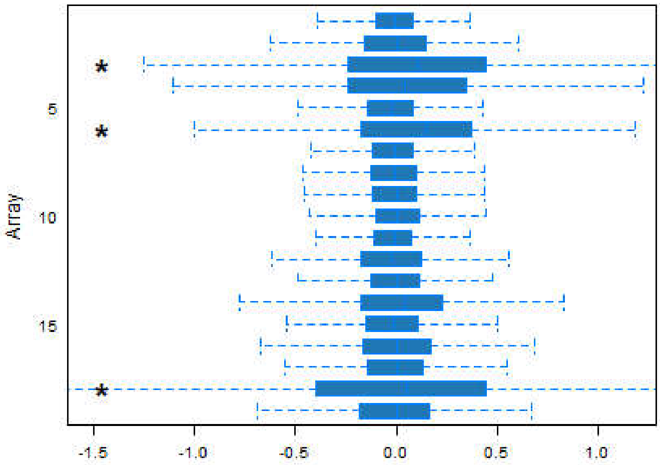

Figure 8 (PDF file) shows the *Relative Log Expression (RLE)* plot. Arrays whose boxes are centered away from 0 and/or are more spread out are potentially problematic. Outlier detection was performed by computing the Kolmogorov-Smirnov statistic R_a between each array's RLE values and the pooled, overall distribution of RLE values.

+ Figure 9: Outlier detection for Relative Log Expression (RLE).

- Figure 10: Normalized Unscaled Standard Error (NUSE).

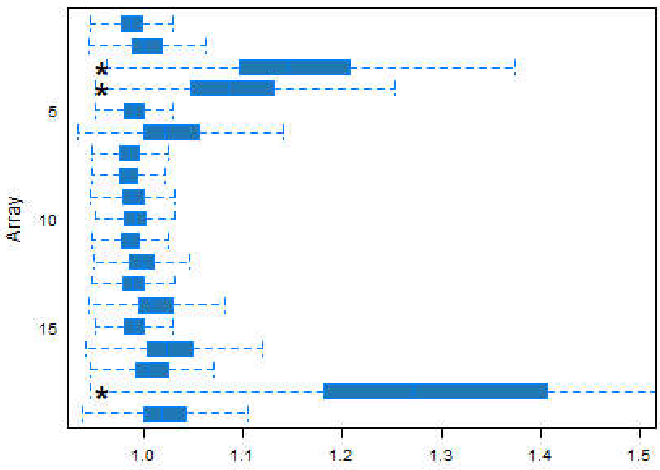

Figure 10 [(PDF file)]() shows the *Normalized Unscaled Standard Error (NUSE)* plot. For each array, the boxes should be centered around 1. An array were the values are elevated relative to the other arrays is typically of lower quality. Outlier detection was performed by computing the 75% quantile N_a of each array's NUSE values and looking for arrays with large N_a.

- Figure 11: Outlier detection for Normalized Unscaled Standard Error (NUSE).

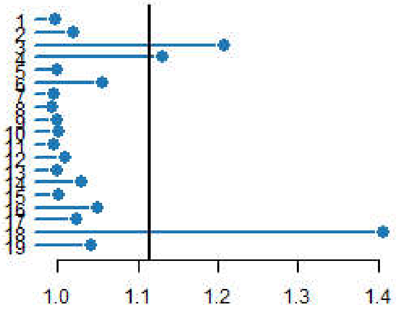

Figure 11 (PDF file) shows a bar chart of the N_a, the outlier detection criterion from the previous figure. The bars are shown in the original order of the arrays. Based on the distribution of the values across all arrays, a threshold of 1.11 was determined, which is indicated by the vertical line. 3 arrays exceeded the threshold and were considered outliers.

- Figure 12: RNA digestion plot.

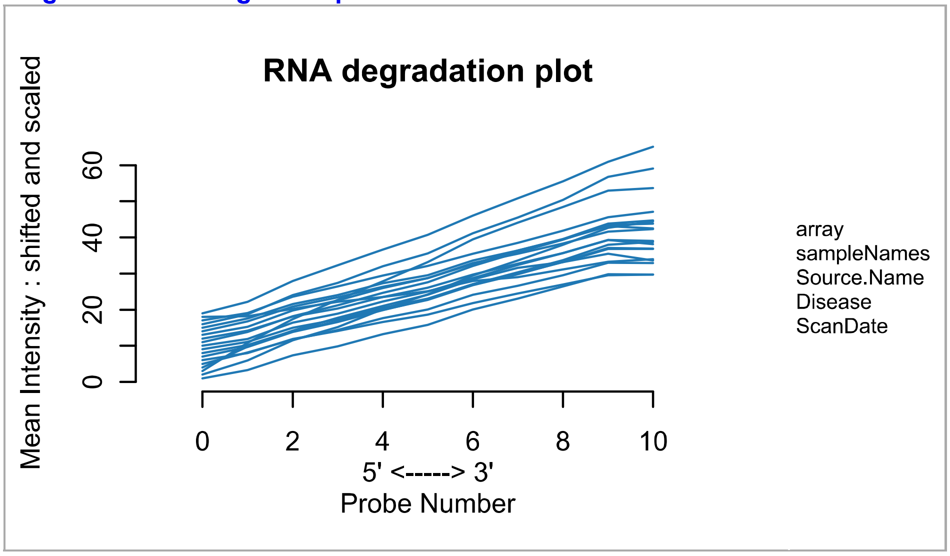

Figure 12 (PDF file) shows the *RNA digestion* plot. The shown values are computed from the preprocessed data (after background correction and quantile normalisation). Each array is represented by a single line; move the mouse over the lines to see their corresponding sample names. The plot can be used to identify array(s) that have a slope very different from the others. This could indicate that the RNA used for that array has been handled differently from what was done for the other arrays.

- Figure 13: Perfect matches and mismatches.

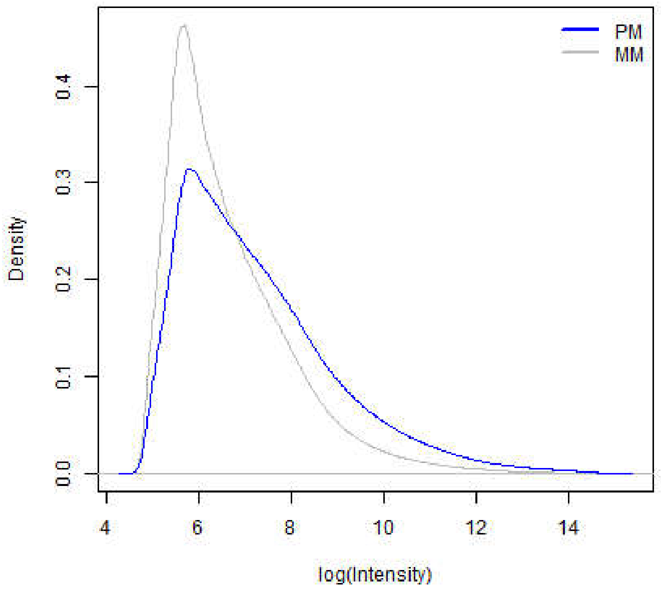

Figure shows the density distributions of the $\log_2$ intensities grouped by the matching type of the probes. The blue line shows a density estimate (smoothed histogram) from intensities of perfect match probes (PM), the grey line, one from the mismatch probes (MM). We expect that MM probes have poorer hybridization than PM probes, and thus that the PM curve be to the right of the MM curve.

Section 5: Individual array quality

- Figure 14: MA plots.

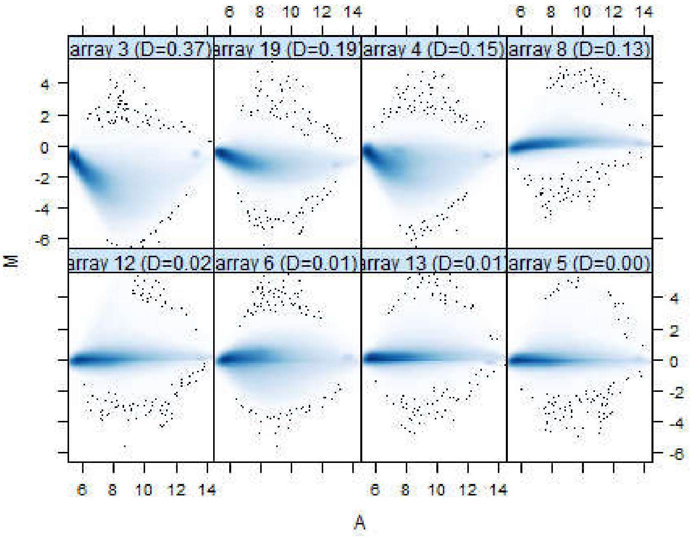

Figure 14 (PDF file) shows MA plots. M and A are defined as:
$M = \log_2(I_1) - \log_2(I_2)$
$A = 1/2 (\log_2(I_1)+\log_2(I_2))$,
where I_1 is the intensity of the array studied,and I_2 is the intensity of a "pseudo"-array that consists of the median across arrays. Typically, we expect the mass of the distribution in an MA plot to be concentrated along the M = 0 axis, and there should be no trend in M as a function of A. If there is a trend in the lower range of A, this often indicates that the arrays have different background intensities; this may be addressed by background correction. A trend in the upper range of A can indicate saturation of the measurements; in mild cases, this may be addressed by non-linear normalisation (e.g. quantile normalisation).
Outlier detection was performed by computing Hoeffding's statistic D_a on the joint distribution of A and M for each array. Shown are first the 4 arrays with the highest values of D_a, then the 4 arrays with the lowest values. The value of D_a is shown in the panel headings. 2 arrays had D_a>0.15 and were marked as outliers. For more information on Hoeffing's *D*-statistic, please see the manual page of the function `hoeffd` in the `Hmisc` package.

- Figure 15: Outlier detection for MA plots.

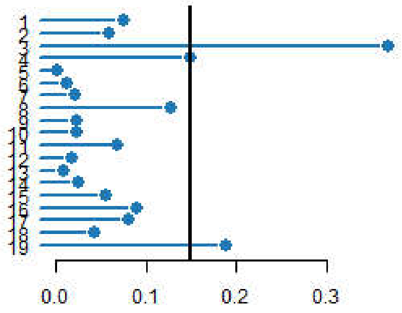

Figure 15 (PDF file) shows a bar chart of the D_a, the outlier detection criterion from the previous figure. The bars are shown in the original order of the arrays. A threshold of 0.15 was used, which is indicated by the vertical line. 2 arrays exceeded the threshold and were considered outliers.

- Figure 16: Spatial distribution of M.

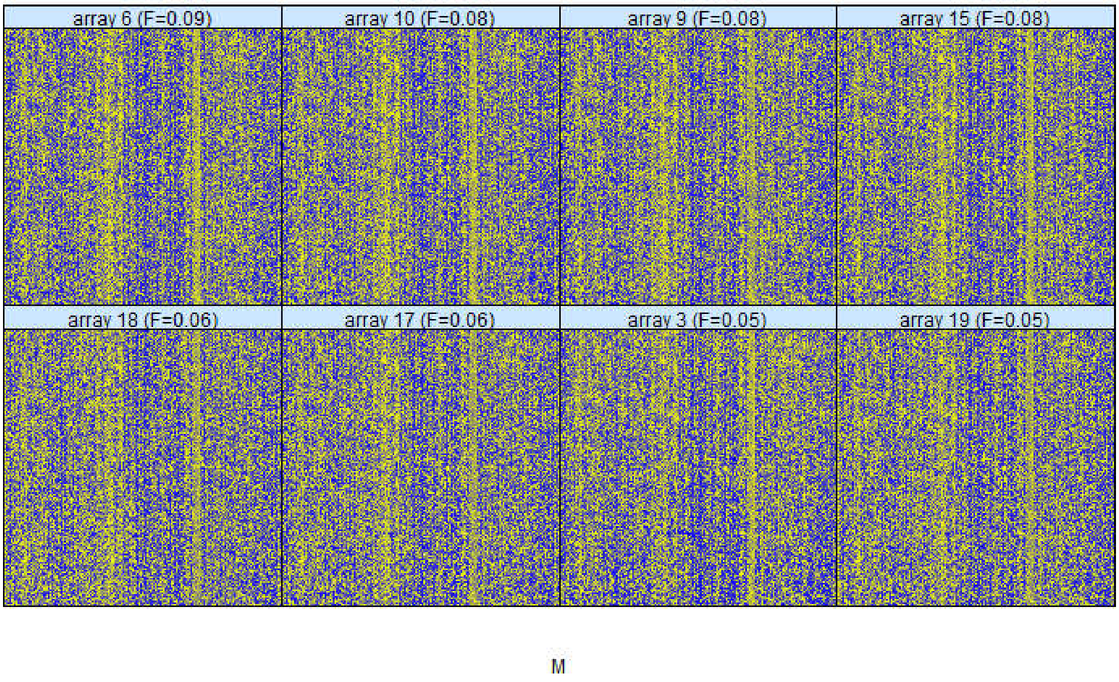

Figure 16 (PDF file) shows false color representations of the arrays' spatial distributions of feature intensities (M). Normally, when the features are distributed randomly on the arrays, one expects to see a uniform distribution; control features with particularly high or low intensities may stand out. The color scale is proportional to the ranks of the probe intensities . Note that the rank scale has the potential to amplify patterns that are small in amplitudebut systematic within an array. It is possible to switch off the rank scaling by modifying the argument`scale` in the call of the `aqm.spatial` function.
Outlier detection was performed by computing F_a , the sum of the absolutes value of low frequency Fourier coefficients, as a measure of large scale spatial structures. Shown are first the 4 arrays with the highest values of F_a, then the 4 arrays with the lowest values. The value of F_a is shown in the panel headings.

- Figure 17: Outlier detection for Spatial distribution of M.

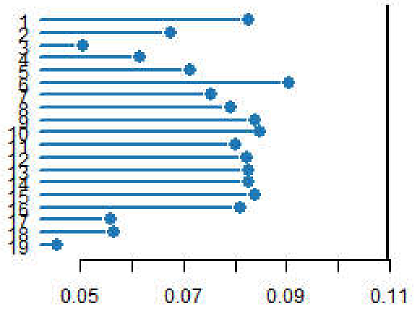

Figure 17 (PDF file) shows a bar chart of the F_a, the outlier detection criterion from the previous figure. The bars are shown in the original order of the arrays. Based on the distribution of the values across all arrays, a threshold of 0.11 was determined, which is indicated by the vertical line. None of the arrays exceeded the threshold and was considered an outlier.

This report has been created with arrayQualityMetrics 3.34.0 under R version 3.4.1 (2017-06-30).

(Page generated on Fri Apr 27 11:48:25 2018 by hwriter)

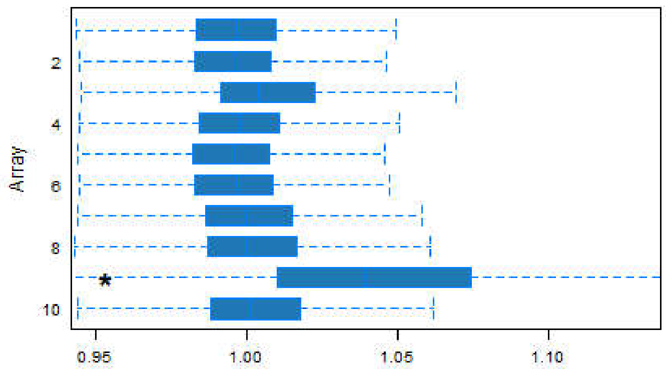

Figure 10 (PDF file) shows the *Normalized Unscaled Standard Error (NUSE)* plot. For each array, the boxes should be centered around 1. An array were the values are elevated relative to the other arrays is typically of lower quality. Outlier detection was performed by computing the 75% quantile N_a of each array's NUSE values and looking for arrays with large N_a.

- Figure 11: Outlier detection for Normalized Unscaled Standard Error (NUSE).

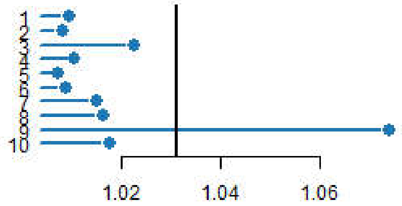

Figure 11 (PDF file) shows a bar chart of the N_a, the outlier detection criterion from the previous figure. The bars are shown in the original order of the arrays. Based on the distribution of the values across all arrays, a threshold of 1.03 was determined, which is indicated by the vertical line. One array exceeded the threshold and was considered an outlier.

- Figure 12: RNA digestion plot.

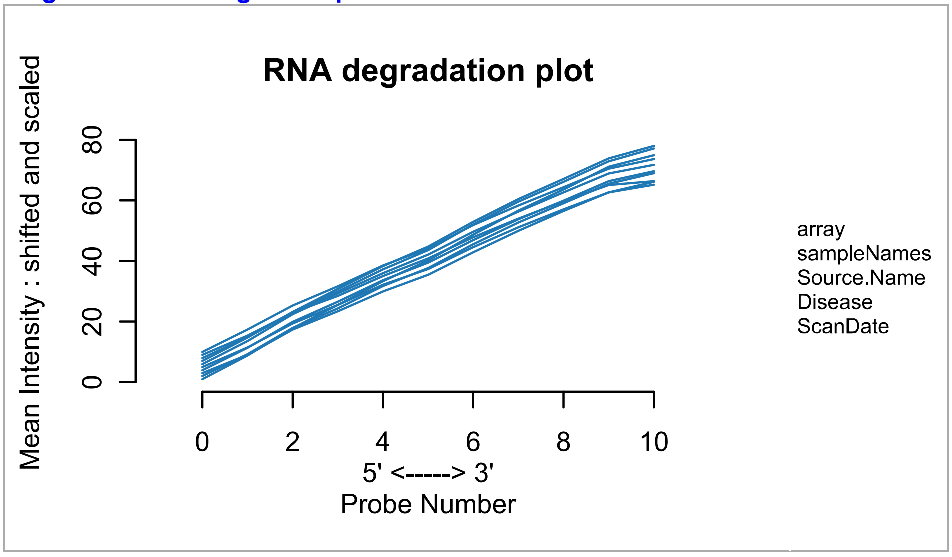

Figure 12 (PDF file) shows the *RNA digestion* plot. The shown values are computed from the preprocessed data (after background correction and quantile normalisation). Each array is represented by a single line; move the mouse over the lines to see their corresponding sample names. The plot can be used to identify array(s) that have a slope very different from the others. This could indicate that the RNA used for that array has been handled differently from what was done for the other arrays.

- Figure 13: Perfect matches and mismatches.

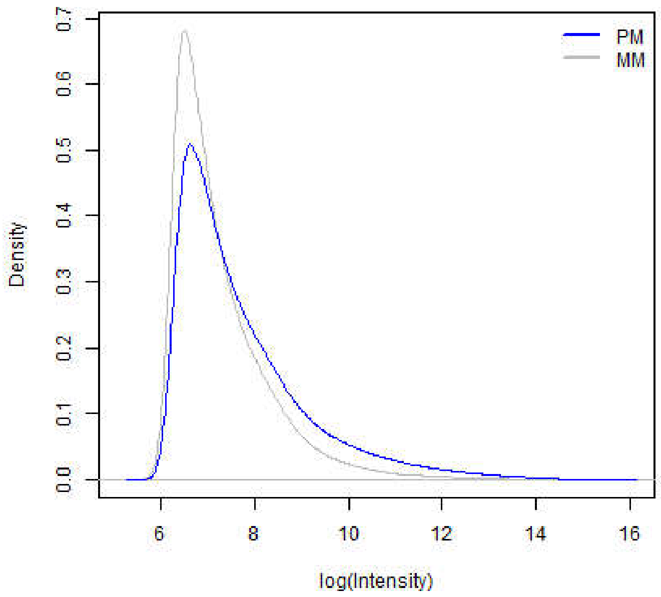

Figure shows the density distributions of the log_2 intensities grouped by the matching type of the probes. The blue line shows a density estimate (smoothed histogram) from intensities of perfect match probes (PM), the grey line, one from the mismatch probes (MM). We expect that MM probes have poorer hybridization than PM probes, and thus that the PM curve be to the right of the MM curve.

Section 5: Individual array quality

- Figure 14: MA plots.

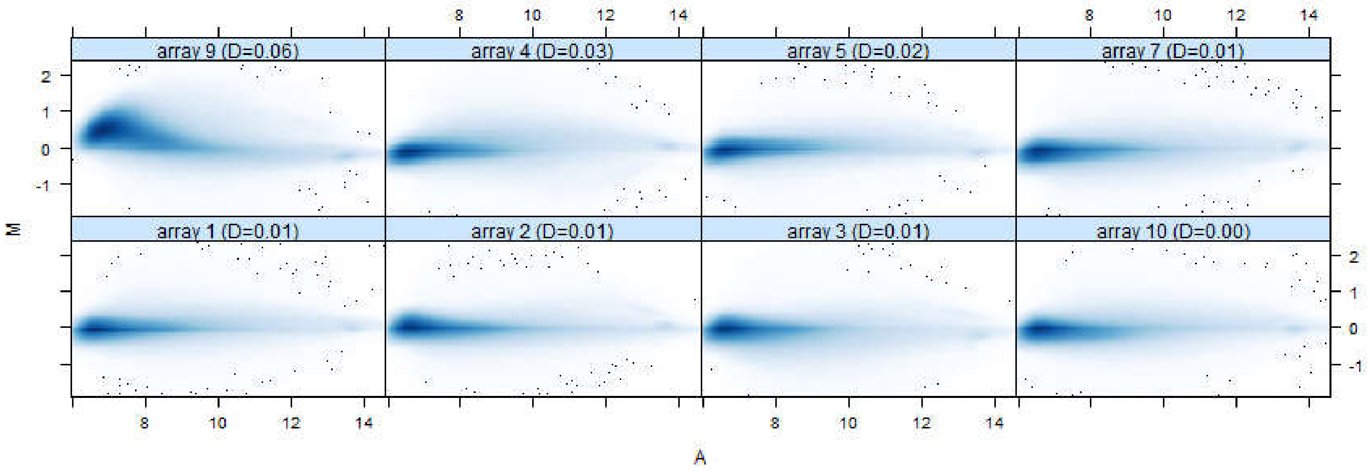

Figure 14 (PDF file) shows MA plots. M and A are defined as:
M = $log_2(I_1)$ - $log_2(I_2)$
A = 1/2 ($log_2(I_1)+log_2(I_2)$),
where I_1 is the intensity of the array studied,and I_2 is the intensity of a "pseudo"-array that consists of the median across arrays. Typically, we expect the mass of the distribution in an MA plot to be concentrated along the M = 0 axis, and there should be no trend in M as a function of A. If there is a trend in the lower range of A, this often indicates that the arrays have different background intensities; this may be addressed by background correction. A trend in the upper range of A can indicate saturation of the measurements; in mild cases, this may be addressed by non-linear normalisation (e.g. quantile normalisation).
Outlier detection was performed by computing Hoeffding's statistic D_a on the joint distribution of A and M for each array. Shown are first the 4 arrays with the highest values of D_a, then the 4 arrays with the lowest values. The value of D_a is shown in the panel headings. 0 arrays had D_a>0.15 and were marked as outliers. For more information on Hoeffing's *D*-statistic, please see the manual page of the function `hoeffd` in the `Hmisc` package.

- Figure 15: Outlier detection for MA plots.

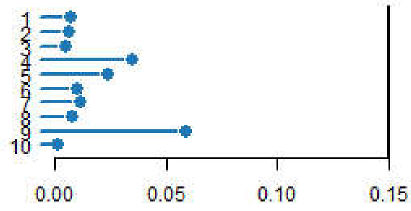

Figure 15 (PDF file) shows a bar chart of the D_a, the outlier detection criterion from the previous figure. The bars are shown in the original order of the arrays. A threshold of 0.15 was used, which is indicated by the vertical line. None of the arrays exceeded the threshold and was considered an outlier.

- Figure 16: Spatial distribution of M.

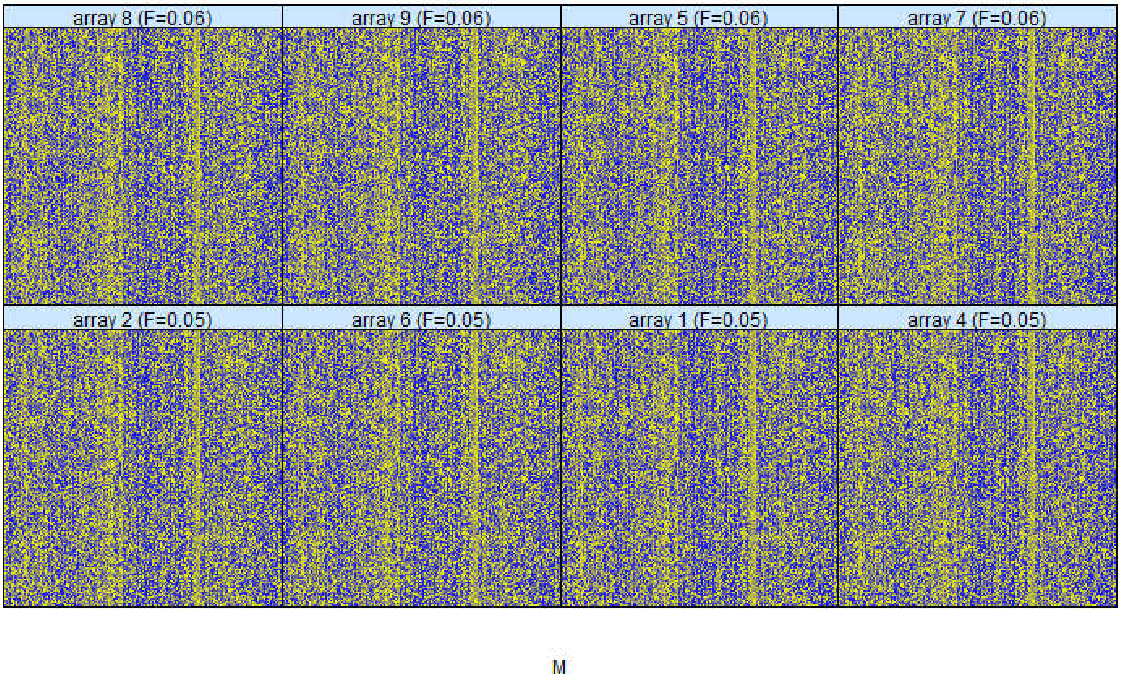

Figure 16 (PDF file) shows false color representations of the arrays' spatial distributions of feature intensities (M). Normally, when the features are distributed randomly on the arrays, one expects to see a uniform distribution; control features with particularly high or low intensities may stand out. The color scale is proportional to the ranks of the probe intensities . Note that the rank scale has the potential to amplify patterns that are small in amplitudebut systematic within an array. It is possible to switch off the rank scaling by modifying the argument`scale` in the call of the `aqm.spatial` function.
Outlier detection was performed by computing F_a , the sum of the absolutes value of low frequency Fourier coefficients, as a measure of large scale spatial structures. Shown are first the 4 arrays with the highest values of F_a, then the 4 arrays with the lowest values. The value of F_a is shown in the panel headings.

- Figure 17: Outlier detection for Spatial distribution of M.

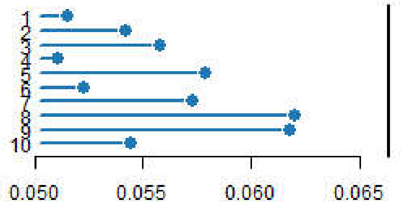

Figure 17 (PDF file) shows a bar chart of the F_a, the outlier detection criterion from the previous figure. The bars are shown in the original order of the arrays. Based on the distribution of the values across all arrays, a threshold of 0.0664 was determined, which is indicated by the vertical line. None of the arrays exceeded the threshold and was considered an outlier.

This report has been created with arrayQualityMetrics 3.34.0 under R version 3.4.1 (2017-06-30).

(Page generated on Fri Apr 27 12:52:12 2018 by hwriter)
